# Supplementary material for: mHealth guideline training for non-communicable diseases in primary care facilities in Nigeria: a mixed methods pilot study
Source: BMJ Open. 2022 Aug 26;12(8):e060304. doi: 10.1136/bmjopen-2021-060304 (PMC9422821; doi:10.1136/bmjopen-2021-060304)
Supplement: Supplementary data [file bmjopen-2021-060304supp001.pdf]

# Tackling non-communicable diseases in primary care facilities Nigeria through clinical guidelines and m-Health training strategies.

## FOCUS GROUP DISCUSSION GUIDE

1. Perception of the use of the m-Health strategy- mode of delivery & content of training resources, challenges they have encountered as well as recommendations on the way forward.
- Satisfaction ( or not) with the skills and knowledge from the training modules
- Were the modules sufficient enough to prepare you to carry out these services (NCD management)

2. Implementation issues;
   - Integrating NCD management into routine activities
   - Role of additional resources in program strengthening- clinical guidelines/ desk guides,
   - treatment cards, basic diagnostic equipment, monthly support for telecommunications

? – how did the project impact/ change your
-workload
-Income
-working relationship with colleagues
- relationship with community

3. Adequacy of Supervision:
   - Clinical supervisory visits ( onsite and remote)
   - Monitoring
4. Moving forward, do you think your facility can continue providing these services even after the project ends?
5. Lessons learned
