# Supplementary material for: mHealth guideline training for non-communicable diseases in primary care facilities in Nigeria: a mixed methods pilot study
Source: BMJ Open. 2022 Aug 26;12(8):e060304. doi: 10.1136/bmjopen-2021-060304 (PMC9422821; doi:10.1136/bmjopen-2021-060304)
Supplement: Author's manuscript [file bmjopen-2021-060304.draft_revisions.pdf]

BMJ Open is committed to open peer review. As part of this commitment we make the peer review history of every article we publish publicly available.

When an article is published we post the peer reviewers' comments and the authors' responses online. We also post the versions of the paper that were used during peer review. These are the versions that the peer review comments apply to.

The versions of the paper that follow are the versions that were submitted during the peer review process. They are not the versions of record or the final published versions. They should not be cited or distributed as the published version of this manuscript.

BMJ Open is an open access journal and the full, final, typeset and author-corrected version of record of the manuscript is available on our site with no access controls, subscription charges or pay-per-view fees (http://bmjopen.bmj.com).

If you have any questions on BMJ Open's open peer review process please email info.bmjopen@bmj.com

BMJ Open

mHealth guideline training for non-communicable diseases in primary care facilities in Nigeria: a pilot study

Journal:	*BMJ Open*
Manuscript ID	bmjopen-2021-060304
Article Type:	Original research
Date Submitted by the Author:	19-Dec-2021
Complete List of Authors:	Otu, Akaninyene; University of Calabar, Internal Medicine<br>Effa, Emmanuel; University of Calabar, Renal Unit, Department of Internal Medicine; University of Cape Town, Division of Nephrology and Hypertension<br>Onwusaka, Obiageli; University of Calabar<br>Omoyele, Chiamaka; Federal Ministry of Health<br>Arakelyan, Stella; Queen Margaret University Institute for International Health and Development<br>Okuzu, Okey; Instrat Global Health Solutions<br>Walley, John; University of Leeds, Nuffield Centre for International Health and Development, Leeds Institute of Health Sciences
Keywords:	PUBLIC HEALTH, Health informatics < BIOTECHNOLOGY & BIOINFORMATICS, EDUCATION & TRAINING (see Medical Education & Training)

SCHOLARONE™
Manuscripts

I, the Submitting Author has the right to grant and does grant on behalf of all authors of the Work (as defined in the below author licence), an exclusive licence and/or a non-exclusive licence for contributions from authors who are: i) UK Crown employees; ii) where BMJ has agreed a CC-BY licence shall apply, and/or iii) in accordance with the terms applicable for US Federal Government officers or employees acting as part of their official duties; on a worldwide, perpetual, irrevocable, royalty-free basis to BMJ Publishing Group Ltd ("BMJ") its licensees and where the relevant Journal is co-owned by BMJ to the co-owners of the Journal, to publish the Work in this journal and any other BMJ products and to exploit all rights, as set out in our licence.

The Submitting Author accepts and understands that any supply made under these terms is made by BMJ to the Submitting Author unless you are acting as an employee on behalf of your employer or a postgraduate student of an affiliated institution which is paying any applicable article publishing charge ("APC") for Open Access articles. Where the Submitting Author wishes to make the Work available on an Open Access basis (and intends to pay the relevant APC), the terms of reuse of such Open Access shall be governed by a Creative Commons licence – details of these licences and which Creative Commons licence will apply to this Work are set out in our licence referred to above.

Other than as permitted in any relevant BMJ Author's Self Archiving Policies, I confirm this Work has not been accepted for publication elsewhere, is not being considered for publication elsewhere and does not duplicate material already published. I confirm all authors consent to publication of this Work and authorise the granting of this licence.

For peer review only

Title:

mHealth guideline training for non-communicable diseases in primary care facilities in Nigeria: a pilot study

Akaninyene Otu[1,2], Emmanuel Effa[1,2], Obiageli Onwusaka[2,3], Chiamaka Omoyele[4], Stella Arakelyan[5], Okey Okuzu[6], John Walley[7]

1. Department of Internal Medicine, College of Medical Sciences, University of Calabar, Calabar, Cross River State, Nigeria
2. Foundation for Healthcare Innovation and Development, Calabar, Cross River State, Nigeria.
3. Department of Public Health Faculty of Allied Medical Sciences, College of Medical Sciences, University of Calabar, Calabar, Nigeria.
4. Monitoring & Evaluation Branch, Non-Communicable Diseases Division, Federal Ministry of Health, Abuja, Nigeria
5. NIHR Research Unit on Health in Fragility, Institute for Global Health and Development, Queen Margaret University, Edinburgh, Scotland
6. InStrat LLC, Montclair, NJ USA
7. Nuffield Centre for International Health and Development, Leeds Institute of Health Sciences, University of Leeds, Leeds, UK

Corresponding Author:

Dr Akaninyene Otu

Department of Internal Medicine, College of Medical Sciences

University of Calabar, Calabar, Cross River State, Nigeria

E mail: akanotu@yahoo.com

For peer review only

Abstract

Design: We report a mixed methods pilot of an innovative mobile health (mHealth) training on NCD care for nurses at PHC facilities in South Eastern Nigeria. We provide a descriptive analysis of mHealth training test scores, with trend analysis of blood pressure control using paired t-test for quantitative data and thematic analysis for qualitative data.

Participants: This pilot involved 23 nurses from 19 PHC facilities in rural and urban communities in Cross River State.

Intervention: We provided mHealth training on an NCD package adapted from the World Health Organization PEN guidelines in collaboration with Nigeria's Federal Ministry of Health. The self-paced training was delivered via android tablet devices with pre- and post-course evaluation, remote and onsite clinical supervision, structured observations and focus group discussions. The overall assessment was the degree to which mHealth training and NCD delivery within primary care was feasible, sustainable, replicable and effective.

Results: Health workers who received mHealth training recorded a statistically significant difference (p=0.000) in average pre-and post-test training scores of 65.2 (±12.2) and 86.5(±7.9) respectively. Recordings on treatment cards indicated appropriate diagnosis and follow-up of hypertensive patients with significant improvements in systolic blood pressure (t=5.09, p<0.001) and diastolic blood pressure (t=5.07, p<0.001). The mHealth training was perceived as a valuable experience and obviated the need for face-to-face training. Increased workload, non-availability of medications, facility-level conflicts, and poor task shifting were identified challenges.

Conclusions: This initiative provides evidence of feasibility of implementing an NCD care package supported by mHealth training for health workers in PHCs, and the strong possibility of successful scale-up nationally.

Article Summary

Strengths and limitations of this study

- Pragmatic implementation of an mHealth training model based on an adapted NCD package of care.
- Process evaluation of feasibility, implementation experience, challenges and patient satisfaction using quantitative and qualitative methods.
- Limitation in patient numbers attending facilities imposed by the COVID-19 pandemic and shortened pilot period leading to reduction in numbers screened, diagnosed, started on treatment and evaluated.
- Perception of the initiative as largely as a non-governmental organisation (NGO) pilot rather than an obligatory health systems strengthening initiative driven by the relevant authorities (State and Federal Ministries of Health)

For peer review only - http://bmjopen.bmj.com/site/about/guidelines.xhtml

Introduction

Non-Communicable Diseases (NCDs) are responsible for the deaths of 41 million people annually with an estimated 15 million of these deaths occurring in people between 30 and 69 years of age.[1] Among NCDs, the four top conditions that together account for more than 80% of all these premature deaths include cardiovascular diseases (17.9 million deaths annually), cancers (9 million), respiratory diseases (3.9 million), and diabetes (1.6 million). Almost three quarters of NCD linked deaths occur in low-and middle-income countries (LMICs). Fragile health systems experience challenges in controlling both NCDs and infectious diseases such as HIV/AIDS and tuberculosis (TB). The rapid global epidemiologic transition and rising rates of NCDs is also having negative effects on the health of Nigerians.

In Nigeria, NCDs are estimated to account for 29% of all deaths[2] with the probability of dying prematurely from NCDs being put at 20% by the World Health Organization (WHO).[3] Community-based surveys involving adults in Cross River Sate Nigeria have identified that approximately 7% of residents in Calabar had undiagnosed diabetes mellitus[4], with reported prevalence of hypertension and obesity being 42% and 18% respectively.[5] It is projected that by 2030, NCDs will be the leading cause of morbidity and mortality in Nigeria.[6,7] This rise in NCDs has been attributed to rapid population growth and urbanisation, limited access to healthy and nutritious food and physical inactivity.[8,9]

The fragile Nigerian public health system is not well equipped to effectively manage and control NCDs.[10] Prior to now, NCD care was not prioritised by policy makers. For example, only 3.3% of the national health budget is voted to mental health care with fewer than 150 mental health specialists serving Nigeria's estimated 206 million population.[11] In 2019, Nigeria's Federal Ministry of Health launched the National Multisectoral Action Plan for the Control and Prevention of NCDs[12], prioritising health systems strengthening at all levels of care towards universal health coverage (UHC). A major thrust of the plan was the integration of NCDs management into primary healthcare (PHC) services. Despite this, the recently launched National task-shifting and task-sharing policy does not cover NCD prevention and control.[13] NCDs are not prioritised in health worker basic or post basic training, as in many other countries. So far, the PHC facilities in Nigeria have not been involved in programmatic/systematic care for chronic diseases.

The Communicable Disease Health Service Delivery (COMDIS-HSD) programme has researched and developed care guideline processes to identify, diagnose and treat NCDs. These packages of care have been adapted and used at provincial scale in Pakistan, Swaziland[14] and elsewhere with higher rates of diagnosis and NCD control in the primary care facilities.[15-20] This NCD package has been scaled-up across the outpatient departments of all public hospitals and many health centres in the Punjab Province, resulting in the diagnosis and treatment of 540,000 people since 2017.[16-20] Similarly, COMDIS-HSD has researched, developed, trialled, and scaled to 71 rural health facilities in China covering 2.5 million people.[21]

For peer review only - http://bmjopen.bmj.com/site/about/guidelines.xhtml

The COMDIS-HSD NCD package has been adapted and adopted in Sierra Leone.[22] It has been also adapted by the Nigerian Federal Ministry of Health (FMoH) NCD technical working group with technical support from the Nuffield Leeds, United Kingdom (UK). The package is technically compatible with the WHO Package for Essential Noncommunicable (PEN) disease interventions and provides steps of medication and patient education (https://comdis-hsd.leeds.ac.uk/wp-content/uploads/sites/50/2018/08/Diagnosis-treatment-desk-guide.pdf). The PEN/COMDIS-HSD package has been piloted in its hard copy residential course format by the FMoH/NCD division in Abuja Federal Capital Territory. However, there is no funding for scaling -up these traditional in-class service courses for health workers at a national level.

By leveraging the high mobile phone penetration in Nigeria, we aimed to pilot the use of an innovative mobile health (mHealth) user-friendly view-through rate (VTR) mobile training application that can be used on any smart phone, tablet or computer, off-line or while connected to the internet. The guide and training modules were adapted for mHealth online delivery by Instrat Global Health Solutions (an indigenous technology company) with pre/ post tests and award of continuing professional development certificates. Our overarching goal was to develop a workable and sustainable approach to support health workers in managing NCDs at PHC level which can be scaled-up across Nigeria.

Methods

Design

In this pilot, we adopted a mixed-methods approach to:

(i) Evaluate changes in NCD related knowledge through pre-post training assessments included with the online training.

(ii) Understand service delivery and implementation contextual factors through structured observations and focus group discussions involving the health workers following training.

(iii) Assess outcomes through record review of treatment cards for appropriateness of screening, diagnosis and initiation of treatment and changes in blood pressure etc.

Given the complexity of the NCD package and the contextual factors that can affect the intervention, we have proposed a conceptual framework to explain our initiative (Figure 1). This conceptual framework is an adaptation of programme theory (excluding the impact as not applicable in a pilot). The community level is included, even though the intervention was mainly at the facility level.

Figure 1: Conceptual Framework for mHealth pilot study on non-communicable diseases in primary care facilities in Cross River State Nigeria

For peer review only - http://bmjopen.bmj.com/site/about/guidelines.xhtml

The implementation of this pilot was guided by the proposed theory of change that recognises the various inputs and activities expected to generate patient related outcomes and health systems impact driven by policy change. The theory is underpinned by contextual factors such as the current organisation and resourcing of the three-tiered health system, current limitation placed on scope of tasks performed by health workers at PHCs, availability of funding to train health workers and the opportunity to leverage mobile phone penetration. It was not possible to involve patients or the public in the design, or conduct, or reporting, or dissemination plans of our research

Setting and participants

This pilot was carried out in a total of 19 PHC facilities in Cross River State, South-Eastern Nigeria over 12 months between February 2020 and January 2021. It involved 23 nurses drawn from selected sites in rural areas and peri-urban areas in Cross River State.

Process

A one-day face-to-face orientation was organised in Calabar, the capital of Cross River State, in February 2020. This introduced the 23 health workers to the NCD training package which included a desk guide, job aide, treatment cards and access and use of the mHealth training modules. The mHealth modules were developed using the *MediXcel Lite* health technology platform which has been previously described.[23,24] The training modules consisting of video and text-based learning materials were created in English which was understood by all health workers. Several facilitation techniques were used by the trainers such as practical sessions, group discussions and case studies. All health workers were then expected to complete the eight NCD modules over a two-week period by accessing the NCD materials on their mobile phones and tablets. User logins were created for the health workers by the staff of InStrat to enable them download the App onto their android mobile phones. This App also tracked their progress with the NCD modules automatically.

Following this orientation, the health workers were expected to utilise the information contained in the modules to screen and identify patients presenting with NCDs to their various facilities. Treatment cards were distributed, and participants were instructed to upload monthly updates on the number of NCD cases seen in the various facilities via an electronic *Comm Care* App portal on the mobile electronic devices. Relevant clinical materials such sphygmomanometers, glucometers and test strips were provided for the facilities to facilitate the clinical assessment of clients with NCDs.

The health workers were provided with funds monthly to cover telecommunications fees. A community of practice (CoP) WhatsApp group was created for the health workers to facilitate peer-to-peer interactions, and this was supported by the clinical research team. The WhatsApp group also served as an avenue to provide non-financial incentives such as recognition on the platform. The health workers who uploaded their facility data onto the

For peer review only - http://bmjopen.bmj.com/site/about/guidelines.xhtml

Comm Care App on time were openly commended and this appeared to motivate others to do the same.

Supportive supervision was provided to the health workers in the form of weekly conference telephone calls and quarterly facility visits. The facility visits involved first-hand assessments of the service delivery contextual factors. During these trips, clinical mentoring was provided, and the health workers were observed providing care to patients using the clinical desk guide and treatment cards. Feedback was obtained from the health workers during these sessions. Instrat staff also had bi-weekly follow-up calls with the health workers to address any information technology (IT) issues and encourage them to upload data on their facilities.

A new module focusing on integrating NCD management into daily activities of PHC facilities was developed and deployed on the training App.

Focus group discussions

In the 5th month of implementation, focus group discussions (FGDs) were conducted with the health workers. Shorts message service invitation were sent to 24 participants who completed the intervention to share their perceptions of the digital training and experiences in focus group discussions. Focus groups[25] were the chosen method as they are useful for exploring perspectives and encouraging reflections on experiences. Twenty-three participants accepted the invitation. We conducted four focus group sessions over 10 days, with an average of 6 participants in each group (FG1 n=8, FG2 n=4, FG3 n=5, FG4 n=6). There were only two male participants, and they were part of the fourth group.

Each FGD was facilitated by 2 researchers experienced in conducting FGDs and an assistant. The research assistant coordinated the session schedules, recording and served as the official note-taker. Sessions were 1 – 2 hours in length and conducted face-to-face in the place convenient for participants. Open-ended questions, formulated by the team who have formal training in qualitative research were used to gain the best perspective and scope of individual responses on (1) perceptions of the use of m-Health training strategy, (2) experiences with the implementation of clinical guidelines (3) reflections on the adequacy of received supervisions, (4) reflections on potential scale-up of the intervention. Facilitator notes ensured uniformity among FGDs and kept each discussion on target. At each FDG, an opportunity was given to withdraw consent to participate and recording. Responses were recorded, and participants were assured their comments would be kept confidential and anonymous.

The COVID-19 pandemic had a negative impact on the number of clients presenting for care in the pilot health facilities. Community mobilization was conducted to help overcome some social barriers to accessing care that came in the wake of the COVID-19 pandemic. Some of the health facilities were vandalised during the nationwide protests that engulfed Nigeria in October 2020, and this also had a negative impact on the NCD care services.

For peer review only - http://bmjopen.bmj.com/site/about/guidelines.xhtml

No formal sample size was calculated for this pilot study.

Data analysis

Quantitative data

The screening, diagnosis, treatment and follow-up care is specified in the NCD guide and this information is recorded on the treatment card. The record review was of all patients identified and initiated on care. The assessment was of quality care according to these guidelines and the completeness of recording of key clinical measurements of blood pressure (BP), body mass index (BMI), fasting blood sugar (FBS) and urine analysis (as per guideline for that disease) were metrics used to access the effectiveness of the pilot project. We assessed the effect of the NCD mHealth training through the pre/post test results and assessed operational issues that may have contributed to the results. The overall assessment was the degree to which the NCD mHealth training and care was feasible, sustainable, replicable, and treatment initiation quality was (as per guideline).

Data were imported and analysed in SPSS v23.[26] Descriptive analysis of quantitative data was carried out using means, standard deviations, frequencies, and percentages. Group differences were established using chi-square analysis. Descriptive trend analysis for the follow up rates in the systolic blood pressure and diastolic blood pressure changes in the hypertensive patients was conducted on the monthly basis. The paired t-test was used to compare the average change in systolic blood pressure and diastolic blood pressure levels at baseline and three-month follow up among the hypertensive patients. Hypothesis testing was two-tailed, at the 5% level.

Qualitative data

We used qualitative thematic analysis to identify prominent themes and patterns in the data and included a combination of deductive and emergent strategies. The facilitators debriefed immediately after each FGD. Two researchers (AO and SA) independently reviewed the data and came together to discuss emerging ideas. After the discussion, the researchers returned to the data to further review and confirm coding. This iterative process resulted in grouping the codes into three major themes: (1) perceptions with the mHealth trainings, (2) experiences with the implementation of NCD care packages, (3) potential issues with the intervention scale-up. Qualitative data were analysed using Dedoose software.[27]

The development of the research question and outcome measures in this initiative were directly informed by Nigerian patients' priorities, experience, and preferences. These priorities were factored into the design of this study. Patients were not directly involved in the recruitment to and conduct of the study but were beneficiaries of the NCD package of care we provided in the PHCs. We aim to disseminate the results of this initiative by conducting interactive sessions with the patients who continue to receive care for their NCDs in the PHCs in Cross River State.

For peer review only - http://bmjopen.bmj.com/site/about/guidelines.xhtml

Results

A total of 23 health workers from 19 primary health centres (PHCs) across the state were trained using the App. The average pre-test and post-test scores were 65(±12) and 87(±8) respectively with a statistically significant difference between these scores following a two-tailed t-test (p=0.000).

Since the project initiation, 561 (71%) community members served by 19 targeted centres were diagnosed with NCDs and 569 patients were served in total. Average age of NCD diagnosis for males was 53 years (SD= 15) and for females 49 years (SD=14). Over three-quarters (n=432, 76%) of all cases and over half (n=315, 55%) of newly diagnosed cases were hypertensive patients (newly diagnosed:110 male, 205 female, average age 52, SD=13) and 14% were diabetics (15 male, 35 female, average age 49, SD=14) (Table 1).

Fasting blood sugar checks were conducted on 215 of the community members with 47 (22%) of them falling within the prediabetes range of 5.6-6.9mmol/l and 82 (38%) falling above the diabetes threshold of >7.0mmol/l (Table 1). Proteinuria and/or glycosuria was identified in 14 of the 113 persons who had a urine analysis. In accordance with the NCD guideline, fasting blood glucose was only done if the person was overweight or had related symptoms, and urine analysis was only done as indicated.

Table 1. Characteristics of NCDs patients served by targeted PHC clinics n=569

**Characteristics**	**Male (n=206/36%)**	**Female (n=363/64%)**	**Total (n=569)**
Age (µ ± SD)	53.5±15.2	49.75±14.2	51.8 ±14.2
**NCD patients who attended facilities**			
Hypertension	155 (75.2%)	277 (76.3%)	432 (75.9%)
Diabetes	23 (11.2%)	47 (13.0%)	70 (12.3%)
Asthma/COPD	10 (4.9%)	17 (4.7%)	27 (4.7%)
Depression	1 (0.5%)	8 (2.2%)	9 (1.6%)
Sickle cell anaemia	6 (3.0%)	1(0.3%)	7 (1.2%)
Epilepsy	3 (1.5%)	3 (0.8%)	6 (1.1%)
Other NCD	8 (3.9%)	10 (2.8%)	18 (3.2%)
Total	206 (100%)	363 (100%)	569 (100%)
**New diagnosis of NCDs**			
Yes	145 (70.4%)	269 (74.2%)	414 (72.7%)

For peer review only - http://bmjopen.bmj.com/site/about/guidelines.xhtml

No	61 (29.6%)	94 (25.8%)	155 (27.3%)
Total	206 (100%)	363 (100%)	569 (100%)
**Check-up items**			
Height (μ ± SD)	1.56±0.14	1.56±0.11	1.56±0.12
Weight (μ ± SD)	67.4±12.6	68.5±12.4	68.1±12.5
**BMI**			
Underweight	9 (4.4%)	3 (8%)	12 (2.1%)
Normal 18.5-24	57 (27.7%)	114 (31.4%)	171 (30.0%)
Overweight 25-30	78 (37.9%)	124 (34.1%)	202 (35.5%)
Obese ≥30	62 (30.1%)	122 (33.6%)	184 (32.3%)
Total	206 (100%)	363 (100%)	569 (100%)
**Blood pressure (BP)**			
Normal BP 90/60mmHg-120/80mmHg	52 (25.2%)	73 (20.1%)	125 (22.7%)
High normal 130/85mmHg - 139/89mm Hg	21 (10.2%)	38 (10.5%)	59(10.4%)
High BP ≥140/90mmHg	133 (64.6%)	252(69.4%)	385 (67.7)
Total	206 (100%)	363 (100%)	569 (100%)
**Diagnostic tests**			
**Fasting blood sugar (FBS)**			
Normal reading <5.6mmol/l	30 (40.0%)	56 (40.0%)	86 (40.0%)
Prediabetes 5.6-6.9mmol/l	15 (20.0%)	32 (23%)	47 (21.9%)
Diabetes reading >7.0mmol/l	30 (40.0%)	52 (37.1%)	82 (38.1%)
Total	75(100%)	140 (100%)	215 (100%)
**Urine analysis**			
Normal	32(69.6%)	57(65.5%)	89 (66.9%)
Proteinuria and/or glycosuria[§]	14 (30.4%)	30(34.5%)	44 (33.1%)
Total recorded	46(100%)	87(100%)	133 (100%)

For peer review only - http://bmjopen.bmj.com/site/about/guidelines.xhtml

§Proteinuria- proteins in urine, glycosuria- sugar in urine. According to the NCD guideline, fasting blood glucose taken if overweight or have related symptoms, and urine analysis as indicated.

Gender distribution of newly diagnosed NCD cases (Figure 2) was not statistically different $\chi2$ (6) =8.4, p=0.2. On average, 16 hypertensive and 3 diabetic patients were identified per facility (range 3-38 for hypertensive and 0-12 for diabetic patients).

Figure 2. Gender distribution of newly diagnosed NCD cases

All NCD patients (n=569) registered at targeted facilities received height (μ=1.56, SD=0.12) and weight measurements (μ=68.1, SD=12.47) at the first appointment. BMI was calculated and recorded for all of these patients (μ=28.1, SD=5.85). Over forty percent of NCD patients (n=234, 41%) received an appointment reminder following the screening appointment.

Healthy lifestyle advice

Health workers briefly discussed with patients key messages related to lifestyle change, disease education, adherence to clinical appointments and medication. For these a motivational counselling strategy was used. The most common lifestyle messages were on healthy eating, including reduction in the salt intake; increase in physical activity and regular exercising.

Primary outcome

Recordings on treatment cards indicated a positive significant improvement in blood pressure of hypertensive patient from visit 1 through to visit 3 (Figure 3). The systolic blood pressure for hypertensive patients steadily decreased from the screening (baseline) average of 160 to a visit 3 average of 135 mmHg. (Table 2). The average systolic blood pressure decreased 23 mmHg between the screening to follow-up visit 3 (p<0.001). A similar trend was registered in average decrease of 12mmHg in diastolic blood pressure from screening (baseline) of 96mmHg to follow-up visit 3 average of 79mmHg (p<0.001).

Figure 3: Trends in blood pressure change among hypertensive patients (n=428)

Table 2: Trend on blood pressure from visit 1 to visit 3 based on average of two measurements

Parameter	Value	Visit 1	Visit 2	Visit 3
Systolic BP (mmHg)	Valid values	416	227	96
	Mean (±SD)	159.9 (23.3)	143.3 (20.8)	139.5 (19.2)
	Range	90-270	99-218	80-180
	Values >140 (%)	60.7%	20.8%	7.5%
	Values >180 (%)	14.5%	2.1%	--

For peer review only - http://bmjopen.bmj.com/site/about/guidelines.xhtml

Diastolic BP (mmHg)	Valid values	416	227	96
	Mean (±SD)	95.9 (13.9)	85.1 (13.3)	81.6 (11.2)
	Range	50-152	80-130	50-120
	Values >90 (%)	49.1%	9.8%	2.8%
	Values >110 (%)	10%	2.1%	0.2%

Compliance with medication

The hypertensive patients were prescribed antihypertensive drugs from the following classes: (1) Angiotensin-converting enzyme inhibitors (lisinopril, captopril), (2) Calcium channel blockers (nifedipine, amlodipine), (3) Diuretics (furosemide, hydrochlorothiazide) which were all in line with recommendations provided in the NCD deskguide. Where hydrochlorothiazide was unavailable, normoretic (i.e. amiloride hydrochloride 5mg and hydrochlorothiazide 50mg) was prescribed. The top three most often prescribed antihypertensive drugs were normoretic (50%), nifedipine (44%) and amlodipine (33%). Diabetic patients were mostly prescribed metformin, glibenclamide, or insulin injections.

Focus group discussions

Participants

Focus groups included 21 women and 2 men, between 31 to 60 years of age. All participants had post-secondary training in nursing. Some of the participants had prior experience of attending digital training. However, NCDs had not been prioritised in the health worker basic or post basic training the participant had previously received.

Perceptions of m-health training

Content

The mHealth training was stated to be a valuable experience. Participants were very appreciative of the knowledge they acquired about prevention and control of NCDs. They talked about improved confidence and an increased sense of empowerment in their ability to provide counselling on lifestyle behaviours and medication adherence. This positively influenced their internal sense of importance, consistent with improved self-efficacy.

> *"Some of these cases … before now I didn't know how to handle them but now I am very confident in handling the issues. The way and manner I am able to regulate clients' blood pressure are making me feel important" [FG1].*

> *"I will begin by appreciating the NCD project and people. Before now, we actually didn't know how to handle these cases but now we are very confident. We know the particular drugs to prescribe and the dosage". [FG2]*

For peer review only - http://bmjopen.bmj.com/site/about/guidelines.xhtml

Functionality

Participants perceive the functionality of m-health technology and ease of use as important. Multiple benefits of online learning were noted including the ability to learn at your own pace, the opportunity to return to the material when needed, using smart phones to access on the VTR the NCD deskguide and modules.

> *"We are upgraded. It makes the provider more educated, because you read at your pace, you don't stress yourself, anytime you come back you read and understand. So.. I think it was great."[FG1].*

> *"It was a nice one. Almost all the trainings we have attended have not been this type. It has made us to know how to use our phone in learning online and also searching for other things you would not have ordinarily done because of the workload where we find ourselves. It is a good one. Many times, we tell ourselves let's go online something is happening "[FG4].*

Experiences with the implementation of NCD care package

Positive impact

Participants talked highly about positive changes the integrated care delivery brought to the communities they served. Provision of BP and blood glucose diagnostic equipment meant targeted diagnostic and curative services delivery at a point of access.

> *"The NCDP [the project] has made us have the necessary equipment to carry out blood pressure, diabetes diagnosis in my facility. This is a great achievement really. People come in now to say I want to check my blood pressure and sugar." [FG4]*

> *When people come to our ANC, we have some of the test kits to use and we started testing. We admitted some people. When people come to visit them, they go back to the community to tell others. Presently, we have increased client flow. It is helping us to detect these NCDs". [FG4]*

The provision of PHC facility services dramatically improved relationships with communities and encouraged follow-up visits. During outreaches, health workers were able to use the donated sphygmomanometers to measure blood pressure within the communities and refer in people to the PHC facility.

> *"The relationship with the community as a result of the NCD is marvellous wherever we meet with these clients they greet one with respect and are open to me" [FG1]*

For peer review only - http://bmjopen.bmj.com/site/about/guidelines.xhtml

> *"Giving the test that was not existing in the community before really help to mobilize the people. They feel more relaxed and confident to come to the facility".[FD4]*

Participants described a variety of community education strategies to raise awareness of the availability of NCD services. Some strategies involved monetary reward for spreading the word about services by the town crier, and also use of local worship places and/or community organisation to engage with communities.

> *"…I have a relationship with the town announcer. Although when people come and take the test, we collect a token from them, and I use that to pay the town announcer. I type what I want him to announce. He reads through it and announces it to the people. From there people started coming". [FG4]*

> *"This project has impacted on our knowledge, and we can confidently do health talk and create awareness about blood pressure, and other NCDs efficiently. My wish is.., I hope this project stays, honestly." [FG1]*

Challenges

Participants, however, also experienced, or perceived challenges to the implementation of care packages as extending across clinical and organisational levels.

Challenges at the clinical level

Factors identified as challenges at the clinical level included increased workload associated with (a) ensuring the continuity of NCD care delivery, and (2) filling in the treatment cards. Participants noted the NCD initiatives' success meant the increased flow of patients to facilities for check-ups and follow-up consultations. Although this has generally been perceived as a positive dynamic, the increase in workload was mentioned.

> *"NCDs increased our work. Before now, we see them and they go away, we don't bother to follow them up or they don't come back to us to maintain their BP. We need to maintain them to get their drugs or make sure their blood sugar level got controlled. Some of us, our facilities are easy to get to, so they can easily come. Even if you are resting, they want you to come and check them. When they heard that someone in the community died due to high blood pressure, that period a lot of people came for checking [FG4].*

Some participants also noted that filling the treatment cards and registers was time-consuming and often discouraged their use. Content reduction was a proposed solution to address this.

For peer review only - http://bmjopen.bmj.com/site/about/guidelines.xhtml

> *"Ordinarily we have few questions to capture from clients but the NCD has widened a lot of information that needs to be captured, looking at this with other registers in the facility too has made the workload high, and again there are series of tests that also needs to be run on an individual basis." [FG1].*

Technical glitches in the application and internet connection were also noted resulting in issues with uploading data into the central system. This resulted in double entry of data which was perceived as a double burden on staff. Some participants also recognised that supervision and monitoring provided by staff of the initiative helped them navigate many challenges along the way.

Challenges at the organisational level

The presence of professional support or opposition was highlighted as a considerable factor for the successful integration of the care packages into daily routine. Participants talked in length about conflict of interest between the focal person responsible for NCD care and PHC facility in-charge. Examples of interference from *person-in-charge* into modes of care delivery, equipment storage and use as well as generated income distribution were noted.

> *"The thing is really disturbing because the in-charge kept telling you that the equipment is not for you and should be kept in the laboratory, giving unnecessary orders before you can make use of it. It pissed me off a lot." [FD1].*

Some participants, on the other hand, talked positively about PHC facility in-charge and the support they received. They also acknowledged how some managers actively "champion" the awareness-raising activities amongst communities, and how this considerably increased the flow of patients to facilities.

> *"The new in charge has leadership skills. He went to the community heads and introduced himself, he went to churches. He will encourage sick people to always come and test. A lady died and it was discovered her blood pressure was high. So, they announced in all the churches that people should go and check their blood pressure and sugar level. This really helped us. [FG4]*

Although many participants talked in terms of conflict, in general, negotiation, avoiding confrontations and/ or for managers undergoing training on NCD care alongside junior staff were preferred approaches to resolving the problems.

> *"I believe they should train them. Since we have this knowledge, they know some of the drugs, but they don't know the right time to administer. If they are exposed to this training and they are told the stages and when to prescribe, they will know what*

For peer review only - http://bmjopen.bmj.com/site/about/guidelines.xhtml

to do. For example, in my facility my in-charge prescribed stage II drugs instead of stage I, I had to correct him. If they know, they will do better." [FG4]

Participants also noted negative changes in the spirit of collegiality, suggesting that having a certificate of completion would help to increase credibility among the colleagues and ensuring that *"the focal persons"* have the expertise to manage NCD cases.

Potential issues with the intervention scale-up

Participants talked about pressing issues in a healthcare system that potentially can have a negative impact on the scale-up of the intervention. The most often mentioned challenges identified were health personnel shortage (especially nurses), lack of knowledge on NCD management and control, lack of essential diagnostic equipment and medication necessary for effective NCD care delivery. The intervention included to train one nurse (a 3-year trained registered nurse).

Potential key solutions for ensuring the sustainability and potential scale-up of care packages at a national level were (a) targeted training of other staff on NCD prevention and management, and (b) task-shifting activities for NCD care. Participants also noted that staff at primary care level lacked basic knowledge on NCD care and if left at their own disposal many essential diagnostic tests will be missed. Therefore, they will be willing to provide training to junior staff to ensure effective delivery of services.

"Yes…, because some things, if left for the facility to carry on, will not be effective, but with a focal person she will ensure things are in order, since they know they will be held responsible" [FG1].

"Yes, like in my facility I am the only staff I will appreciate if another staff is sent here so that we can have more hands, and I will give the person on the job training or some kind of step-down training to enable whoever is coming to render the services. It will be bad if this NCDP [the NCD project] stops, before now we have had cases where somebody will just fall while walking. The next thing you hear is people will be saying he is a ghost, not knowing is the effect of high blood pressure. So,…. I really don't want this NCD thing to just fade like that, I don't know how to put it really" [FGD2].

Discussion

Main findings

In this mixed-methods pragmatic implementation of health worker training using an mHealth platform complemented by clinical supervision visits and a community of practice

For peer review only - http://bmjopen.bmj.com/site/about/guidelines.xhtml

forum, we have demonstrated the feasibility of delivering training remotely leading to improvement in knowledge and skills in NCD screening, diagnosis, and treatment. In addition, we have documented nurses' experiences, perspectives, and challenges as well as satisfaction of clients with the care provided which involved shifting of tasks to nurses. This cohort of patients was found to have improved trends on average control of blood pressure. There was a statistically significant reduction on both systolic and diastolic blood pressures between the first and third visits among hypertensive patients. Diuretics and calcium channel blockers (nifedipine and amlodipine) were the most commonly prescribed antihypertensives with metformin and glibenclamide being the most commonly prescribed therapies for diabetes mellitus. These prescribing practices were all in line with the recommendations within the NCD guide.

Health education/counselling was given to the large majority of patients on all visits. The process indicators demonstrate the ability of nurses in PHC clinics to successfully deliver the key components of NCD care. The NCD guide was designed for qualified staff at the level of a 3 year trained registered nurse. The deployment of an mHealth platform rather than a face-to-face classroom style model to deliver the training has been shown to be feasible and effective . This is seen in the improved knowledge scores upon completion of the modules and assessment of skills seen in ability to initiate care for patients with NCDs. The mHealth NCD training was perceived as a valuable experience with the health workers identifying multiple benefits of online learning and the integrated NCD care delivery package.

Challenges to the smooth rollout of this NCD package were the increased workload it generated, the technical problems with internet connection and difficultly in securing the support of health centre in-charges for the project. Remote and periodic onsite supervision by local doctors was an additional strategy adopted to support this initiative.

While the pilot appears to be successful, the sustainability and replicability of this model of training and care initiation will depend on addressing several health system barriers such as appropriate staffing (not all facilities have a nurse), provision of medicines and diagnostic equipment, improving on the governance system at facility level and formally task shifting to nurses the NCD care and prescribing. In addition, the reference to the initiative as a 'project' by nurses implies a risk to sustainability, if the NCD initiative is not wholly adopted by the state health management. Despite its implementation through the routine system by a local medical NGO, the perception of it as a 'project' may undermine the long-term sustainability at facility level.

This mixed methods research adds to the evidence base of similar NCD interventions in resource-limited settings. Using a similar shared care guide for management of diabetes and hypertension care in Eswatini (southern Africa), nurses were able to successfully deliver diabetes and hypertension care with improvements in physiological parameters, though continued training and supervision were required to consolidate change.[14] A similar NCD guideline and training COMDIS-HSD partner initiative in Pakistan was found to be effective

For peer review only - http://bmjopen.bmj.com/site/about/guidelines.xhtml

in trials and was scaled across the Punjab Province of 110 million population.[16-20] This Nigeria pilot adds evidence for the potential in a West African setting and for mHealth as an alternative to expensive residential courses, which is a major constraint to NCD care scale-up.

Limitations

The observational nature of our study precludes from drawing decisive conclusions as to whether care under the new NCD care model is better or worse than the alternative conventional residential training practiced.

The emergence of COVID-19 and imposed public health restrictions substantially limited the number of individuals attending the PHCs to be screened and diagnosed over the equally short period of ten months. In addition, the pragmatic nature of our initiative precluded the provision of medicines, which may have led to an increase in NCD patients observed. Furthermore, given that many NGOs are operating in fragile health environments, pilot initiatives may not be subsequently implemented by ministries of health.

Conclusion

This pilot initiative provides evidence of feasibility of implementing an NCD care package supported by mHealth training for health workers in PHCs as indicated by improvement in BP measurements in follow-up visits. These results support the implementation of such a package of care at scale within the Nigerian context.

Acknowledgements: We appreciate the NCD division of the FMOH, Nigeria, Cross River State Ministry of Health and Primary Healthcare Development Agency as well as nurses and facility heads who participated in the pilot. We are grateful to all the patients who accepted to be managed using the NCD guides in the various health facilities in Cross River State Nigeria. We are thankful to Mr Tijesu Ojumu of the Nigeria Centre for Disease Control for designing the conceptual framework. Our sincere gratitude goes to the NIHR RUHF project of QMU Edinburgh for funding this initiative.

Contributorship statement: AO, EE, ObO and JW conceived the study. AO, EE, ObO, OO and CO were involved in the implementation of the programme. EE, ObO, OO and CO handled the data entry. SA, AO, ObO, JW and OO did the data cleaning and analysis. AO, SA, EE, JW and ObO wrote the first draft. All authors were involved in the reviewing, and editing of this article and gave final approval of the version to be published. AO is the guarantor of the article.

Competing interests: None declared.

For peer review only - http://bmjopen.bmj.com/site/about/guidelines.xhtml

Funding: This study/project is funded by the UK National Institute for Health Research (NIHR) [NIHR Global Health Research programme (project reference 16/136/100)/ NIHR Research Unit on Health in Situations of Fragility]. The views expressed are those of the author(s) and not necessarily those of the NIHR or the Department of Health and Social Care. The funder played no role in the study design, data collection, analysis, interpretation, or in writing the manuscript.

Data sharing statement: Data generated by your research that supports your article will be made openly and publicly available upon publication of your article.

Patient consent for publication: not required

Ethics approval: This study was performed in line with the principles of the Declaration of Helsinki. The study received the approval of the Health Research and Ethics Committee of the Cross River State Ministry of Health Nigeria (Number/ID: CRSMOH/RP/REC/2019/173). Participation was voluntary.

Data availability statement: No data are available.

Supplemental material: This content has been supplied by the authors.

Patient and Public Involvement: The development of the research question and outcome measures in this initiative were directly informed by Nigerian patients' priorities, experience, and preferences. These priorities were factored into the design of this study. Patients were not directly involved in the recruitment to and conduct of the study but were beneficiaries of the NCD package of care we provided in the PHCs. We aim to disseminate the results of this initiative by conducting interactive sessions with the patients who continue to receive care for their NCDs in the PHCs in Cross River State.

References

1. World Health Organization 2021. Noncommunicable diseases – key facts. Available: https://www.who.int/news-room/fact-sheets/detail/noncommunicable-diseases
2. World Health Organization - Noncommunicable Diseases (NCD) Country Profiles. Available: https://www.who.int/nmh/countries/nga_en.pdf?ua=1
3. World Health Organization [WHO]. Global health observatory data 2018: Premature NCD deaths. Available: https://www.who.int/gho/ncd/mortality_morbidity/ncd_premature_text/en/
4. Enang OE, Otu AA, Essien OE, *et al*. Prevalence of dysglycemia in Calabar: a cross-sectional observational study among residents of Calabar, Nigeria. *BMJ Open Diabetes Res Care* 2014; 2(1):e000032. doi: 10.1136/bmjdrc-2014-000032
5. Effa E, Onwusaka, O, Ekrikpo U, *et al*. Community based screening for risk factors of chronic kidney disease in Cross River State. *Kidney International Reports* 2020; 5(3):S58-S59.

For peer review only - http://bmjopen.bmj.com/site/about/guidelines.xhtml

6. Maiyaki M, Garbati M. The burden of non-communicable diseases in Nigeria; In the context of globalization. *Ann Afr Med* 2014;13:1–10.
7. World Health Organization. Noncommunicable diseases: the slow motion disaster. Geneva: WHO; 2017.
8. Crush J, Frayne B, McLachlan M. “Rapid Urbanization and the Nutrition Transition in Southern African.” Urban Food Security Series No. 7. Queen’s University and AFSUN: Kingston and Cape Town. 2011.
9. Maher D, Sekajugo J. Research on health transition in Africa: time for action. *Health Res Policy Syst* 2011; 9:1–4.
10. Green A. Country in focus: the growing burden of NCDs in Nigeria. *The Lancet Diabetes and Endocrinology* 2016; 4(1): 23-24
11. Ajayi T. What is Mental Health to an African? Available: https://www.linkedin.com/pulse/what-mental-health-african-tomiwa-ajayi
12. National multi-sectoral action plan for the prevention and control of non-communicable diseases (2019 – 2025). Available: https://www.iccp-portal.org/system/files/plans/NCDs_Multisectoral_Action_Plan.pdf
13. Federal Ministry of Health Nigeria 2014. Task-shifting and task-sharing policy for essential health care services in Nigeria. Available: file:///C:/Users/akaninyene.otu/Downloads/TSTS.pdf
14. Sharp A, Riches N, Mims A, *et al*. Decentralising NCD management in rural southern Africa: evaluation of a pilot implementation study. *BMC Public Health* 2020*;* 20:44
15. Walley J, Khan MA, Witter S, et al. Embedded health service development and research: why and how to do it (a ten-stage guide). *H Research Policy* & *Systems*. 2018; D-17-*00256R1*
16. Khan MA, Khan N, Walley JD, *et al*. Enhanced hypertension care through private clinics in Pakistan: a cluster randomised trial. *BJGP Open*. 2019; 3(1):bjgpopen18X101617.
17. Khan MA, Walley JD, Khan N, *et al*. Effectiveness of an integrated diabetes care package at primary healthcare facilities: a cluster randomised trial in Pakistan. *BJGP Open.* 2018;2(4):bjgpopen18X101618. doi:10.3399/bjgpopen18X101618
18. Khan N, Khan M, Walley J, et al. A process evaluation of integrated diabetes management at primary health care facilities in Pakistan. *BJGP Open*. 2018;2(4):bjgpopen18X101612
19. Khan MA, Khan N, Walley J, *et al*. How effective is it to deliver integrated COPD care at public healthcare facilities: a cluster randomised trial in Pakistan? BJGP Open 2019; 3 (1). bjgpopen18X101634. ISSN 2398-3795
20. Khan MA, Khan MA, Walley J, *et al*. Feasibility to deliver integrated COPD-Asthma care at primary and secondary level public healthcare facilities in Pakistan: a process evaluation. BJGP Open 2019; DOI: 10.3399/bjgpopen18X101632

For peer review only

21. Wei X, Walley JD, Zhang Z, et al. Implementation of a comprehensive intervention for patients at high risk of cardiovascular disease in rural China: A pragmatic cluster randomized controlled trial. *PloS One* 2017; 12 (8). e0183169.
22. Witter S, Zou G, Diaconu, K. et al. Opportunities and challenges for delivering non-communicable disease management and services in fragile and post-conflict settings: perceptions of policy-makers and health providers in Sierra Leone. *Confl Health* 2020; 14, 3. https://doi.org/10.1186/s13031-019-0248-3
23. Otu, A, Okuzu, O, Ebenso, B, et al. Introduction of mobile health tools to support COVID-19 training and surveillance in Ogun State Nigeria. *Front Sustain Cities* 2021; 3: 10.
24. Otu A, Okuzu O, Effa E, et al. Training health workers at scale in Nigeria to fight COVID-19 using the InStrat COVID-19 tutorial app: an e-health interventional study. *Therapeutic Advances in Infectious Disease* 2021. doi:10.1177/20499361211040704
25. Willis K, Green J, Daly J, *et al*. Perils and possibilities: achieving best evidence from focus groups in public health research. *Aust N Z J Public Health.* 2009;33(2):131–6
26. IBM Corp. Released 2015. IBM SPSS Statistics for Windows, Version 23.0. Armonk, NY: IBM Corp.
27. Dedoose Version 9.0.17, web application for managing, analyzing, and presenting qualitative and mixed method research data (2021). Los Angeles, CA: SocioCultural Research Consultants, LLC www.dedoose.com.

For peer review only

For peer review only - http://bmjopen.bmj.com/site/about/guidelines.xhtml

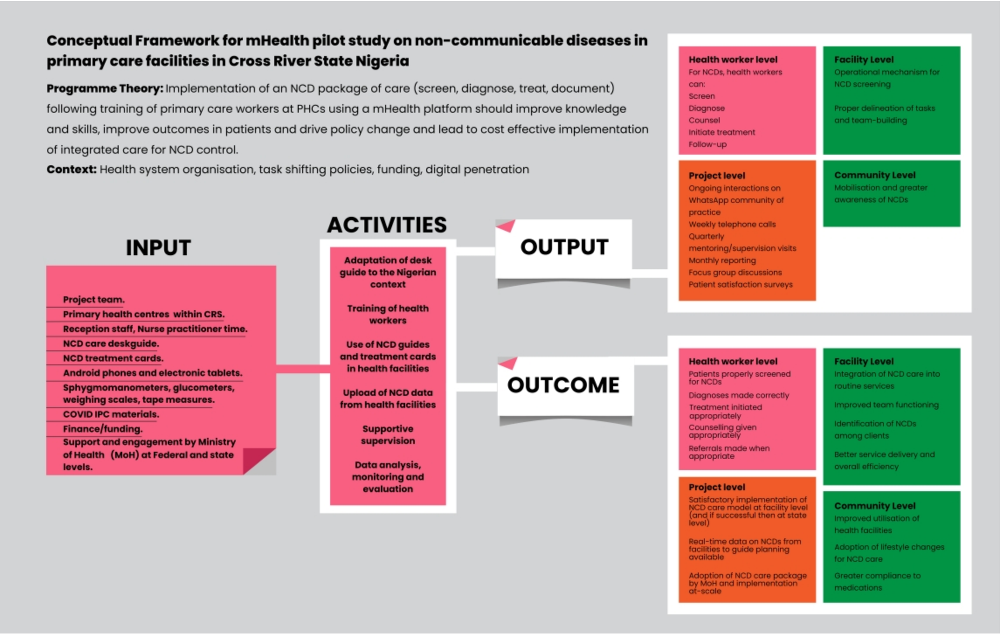

Figure 1: Conceptual Framework for mHealth pilot study on non-communicable diseases in primary care facilities in Cross River State Nigeria

2547x1612mm (72 x 72 DPI)

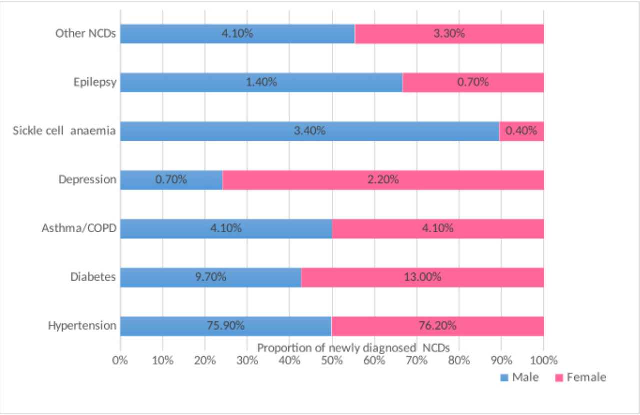

Note. COPD-chronic obstructive pulmonary disease

Figure 2. Gender distribution of newly diagnosed NCD cases

Figure 2. Gender distribution of newly diagnosed NCD cases

22961x32486mm (1 x 1 DPI)

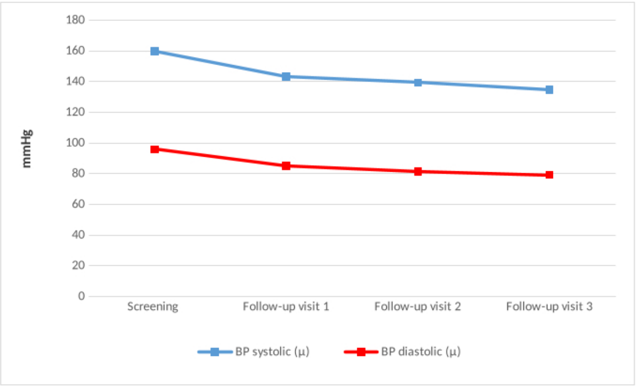

Figure 3. *Trends in blood pressure change among hypertensive patients (n=428)Note. The number of hypertensive patients in follow-up visit 1 (n=227), follow-up visit 2 (n=97), follow-up visit 3 (n=41)*

Figure 3. Trends in blood pressure change among hypertensive patients (n=428)

22961x32486mm (1 x 1 DPI)

Standards for Reporting Qualitative Research (SRQR)*

http://www.equator-network.org/reporting-guidelines/srqr/

Page/line no(s).

Title and abstract

**Title** - Concise description of the nature and topic of the study Identifying the study as qualitative or indicating the approach (e.g., ethnography, grounded theory) or data collection methods (e.g., interview, focus group) is recommended	Page 1, line 2-3
**Abstract** - Summary of key elements of the study using the abstract format of the intended publication; typically includes background, purpose, methods, results, and conclusions	Page 2, line 33-66

Introduction

**Problem formulation** - Description and significance of the problem/phenomenon studied; review of relevant theory and empirical work; problem statement	Page 4, line 113-133
**Purpose or research question** - Purpose of the study and specific objectives or questions	Page 4, line 213-130

Methods

**Qualitative approach and research paradigm** - Qualitative approach (e.g., ethnography, grounded theory, case study, phenomenology, narrative research) and guiding theory if appropriate; identifying the research paradigm (e.g., postpositivist, constructivist/ interpretivist) is also recommended; rationale**	Page 6 and 7, Line197-221
**Researcher characteristics and reflexivity** - Researchers' characteristics that may influence the research, including personal attributes, qualifications/experience, relationship with participants, assumptions, and/or presuppositions; potential or actual interaction between researchers' characteristics and the research questions, approach, methods, results, and/or transferability	Page 6, line 205 -211
**Context** - Setting/site and salient contextual factors; rationale**	Page 4, line 133-145
**Sampling strategy** - How and why research participants, documents, or events were selected; criteria for deciding when no further sampling was necessary (e.g., sampling saturation); rationale**	Page 7, line 245 - 252
**Ethical issues pertaining to human subjects** - Documentation of approval by an appropriate ethics review board and participant consent, or explanation for lack thereof; other confidentiality and data security issues	Page 20, line 656-659
**Data collection methods** - Types of data collected; details of data collection procedures including (as appropriate) start and stop dates of data collection and analysis, iterative process, triangulation of sources/methods, and modification of procedures in response to evolving study findings; rationale**	Page 7, line 245-252

For peer review only - http://bmjopen.bmj.com/site/about/guidelines.xhtml

**Data collection instruments and technologies** - Description of instruments (e.g., interview guides, questionnaires) and devices (e.g., audio recorders) used for data collection; if/how the instrument(s) changed over the course of the study	Page 7, line 245-252
**Units of study** - Number and relevant characteristics of participants, documents, or events included in the study; level of participation (could be reported in results)	Page 12, line 322-325
**Data processing** - Methods for processing data prior to and during analysis, including transcription, data entry, data management and security, verification of data integrity, data coding, and anonymization/de-identification of excerpts	Page 12, line 322-325
**Data analysis** - Process by which inferences, themes, etc., were identified and developed, including the researchers involved in data analysis; usually references a specific paradigm or approach; rationale**	Page 12, line 322-325
**Techniques to enhance trustworthiness** - Techniques to enhance trustworthiness and credibility of data analysis (e.g., member checking, audit trail, triangulation); rationale**	Page 12, line 322-325

Results/findings

**Synthesis and interpretation** - Main findings (e.g., interpretations, inferences, and themes); might include development of a theory or model, or integration with prior research or theory	Page 12-16
**Links to empirical data** - Evidence (e.g., quotes, field notes, text excerpts, photographs) to substantiate analytic findings	Page 17, line 540-579

Discussion

**Integration with prior work, implications, transferability, and contribution(s) to the field -** Short summary of main findings; explanation of how findings and conclusions connect to, support, elaborate on, or challenge conclusions of earlier scholarship; discussion of scope of application/generalizability; identification of unique contribution(s) to scholarship in a discipline or field	Page 3, line 62-72
**Limitations** - Trustworthiness and limitations of findings	Page 19, line 551-559

Other

**Conflicts of interest** - Potential sources of influence or perceived influence on study conduct and conclusions; how these were managed	Page 20, line 654
**Funding** - Sources of funding and other support; role of funders in data collection, interpretation, and reporting	Page 20, line 647-653

*The authors created the SRQR by searching the literature to identify guidelines, reporting standards, and critical appraisal criteria for qualitative research; reviewing the reference lists of retrieved sources; and contacting experts to gain feedback. The SRQR aims to improve the transparency of all aspects of qualitative research by providing clear standards for reporting qualitative research.

For peer review only - http://bmjopen.bmj.com/site/about/guidelines.xhtml

**The rationale should briefly discuss the justification for choosing that theory, approach, method, or technique rather than other options available, the assumptions and limitations implicit in those choices, and how those choices influence study conclusions and transferability. As appropriate, the rationale for several items might be discussed together.

Reference:

O'Brien BC, Harris IB, Beckman TJ, Reed DA, Cook DA. **Standards for reporting qualitative research: a synthesis of recommendations.** *Academic Medicine*, Vol. 89, No. 9 / Sept 2014 DOI: 10.1097/ACM.0000000000000388

For peer review only

For peer review only - http://bmjopen.bmj.com/site/about/guidelines.xhtml

BMJ Open

mHealth guideline training for non-communicable diseases in primary care facilities in Nigeria: a mixed methods pilot study

Journal:	*BMJ Open*
Manuscript ID	bmjopen-2021-060304.R1
Article Type:	Original research
Date Submitted by the Author:	12-Apr-2022
Complete List of Authors:	Otu, Akaninyene; University of Calabar, Internal Medicine<br>Effa, Emmanuel; University of Calabar, Renal Unit, Department of Internal Medicine; University of Cape Town, Division of Nephrology and Hypertension<br>Onwusaka, Obiageli; University of Calabar<br>Omoyele, Chiamaka; Federal Ministry of Health<br>Arakelyan, Stella; Queen Margaret University Institute for International Health and Development<br>Okuzu, Okey; Instrat Global Health Solutions<br>Walley, John; University of Leeds, Nuffield Centre for International Health and Development, Leeds Institute of Health Sciences
**Primary Subject Heading**:	Public health
Secondary Subject Heading:	Global health, Public health
Keywords:	PUBLIC HEALTH, Health informatics < BIOTECHNOLOGY & BIOINFORMATICS, EDUCATION & TRAINING (see Medical Education & Training)

SCHOLARONE™
Manuscripts

I, the Submitting Author has the right to grant and does grant on behalf of all authors of the Work (as defined in the below author licence), an exclusive licence and/or a non-exclusive licence for contributions from authors who are: i) UK Crown employees; ii) where BMJ has agreed a CC-BY licence shall apply, and/or iii) in accordance with the terms applicable for US Federal Government officers or employees acting as part of their official duties; on a worldwide, perpetual, irrevocable, royalty-free basis to BMJ Publishing Group Ltd ("BMJ") its licensees and where the relevant Journal is co-owned by BMJ to the co-owners of the Journal, to publish the Work in this journal and any other BMJ products and to exploit all rights, as set out in our licence.

The Submitting Author accepts and understands that any supply made under these terms is made by BMJ to the Submitting Author unless you are acting as an employee on behalf of your employer or a postgraduate student of an affiliated institution which is paying any applicable article publishing charge ("APC") for Open Access articles. Where the Submitting Author wishes to make the Work available on an Open Access basis (and intends to pay the relevant APC), the terms of reuse of such Open Access shall be governed by a Creative Commons licence – details of these licences and which Creative Commons licence will apply to this Work are set out in our licence referred to above.

Other than as permitted in any relevant BMJ Author's Self Archiving Policies, I confirm this Work has not been accepted for publication elsewhere, is not being considered for publication elsewhere and does not duplicate material already published. I confirm all authors consent to publication of this Work and authorise the granting of this licence.

For peer review only

Title:

mHealth guideline training for non-communicable diseases in primary care facilities in Nigeria: a mixed methods pilot study

Akaninyene Otu[1,2], Emmanuel Effa[1,2], Obiageli Onwusaka[2,3], Chiamaka Omoyele[4], Stella Arakelyan[5], Okey Okuzu[6], John Walley[7]

1. Department of Internal Medicine, College of Medical Sciences, University of Calabar, Calabar, Cross River State, Nigeria
2. Foundation for Healthcare Innovation and Development, Calabar, Cross River State, Nigeria.
3. Department of Public Health Faculty of Allied Medical Sciences, College of Medical Sciences, University of Calabar, Calabar, Nigeria.
4. Monitoring & Evaluation Branch, Non-Communicable Diseases Division, Federal Ministry of Health, Abuja, Nigeria
5. NIHR Research Unit on Health in Fragility, Institute for Global Health and Development, Queen Margaret University, Edinburgh, Scotland
6. InStrat LLC, Montclair, NJ USA
7. Nuffield Centre for International Health and Development, Leeds Institute of Health Sciences, University of Leeds, Leeds, UK

Corresponding Author:

Dr Akaninyene Otu

Department of Internal Medicine, College of Medical Sciences

University of Calabar, Calabar, Cross River State, Nigeria

E mail: akanotu@yahoo.com

For peer review only

Abstract

Objective: To pilot the use of a scalable innovative mobile health (mHealth) non-communicable diseases (NCDs) training application for nurses at primary care level.

Design: Mixed methods pilot of an mHealth training on NCD care for nurses at primary health care (PHC) facilities. We provide a descriptive analysis of mHealth training test scores, with trend analysis of blood pressure control using paired t-test for quantitative data and thematic analysis for qualitative data.

Setting: Primary Healthcare facilities in rural and urban communities in Cross River State, south eastern Nigeria. NCDs was not part of training previously. Here, as in most low-middle income settings, funding for scale-up using conventional classroom in-service training for NCDs is not available, and onsite supervision challenging.

Participants: Twenty-three nurses in 19 PHC facilities

Intervention: Self-paced mHealth training on an NCD desk guide and training module package adapted to be feasible in the country context guidelines in collaboration with Nigeria's WHO and Federal Ministry of Health. Training focused on hypertension, diabetes, and sickle cell disease, and WhatsApp group support was delivered via android tablet devices, supplemented by quarterly onsite supervision. This was evaluated with pre- and post-course tests structured observations and focus group discussions. This was an implementation pilot assessing the feasibility and potential effectiveness of mHealth training and NCD and primary care delivery.

Results: Nurses who received mHealth training recorded a statistically significant difference ($p=0.000$) in average pre-and post-test training scores of 65.2 (±12.2) and 86.5(±7.9) respectively. Recordings on treatment cards indicated appropriate diagnosis and follow-up of hypertensive patients with significant improvements in systolic blood pressure ($t=5.09$, $p<0.001$) and diastolic blood pressure ($t=5.07$, $p<0.001$). The mHealth nurse training and WhatsApp support groups were perceived as a valuable experience and obviated the need for face-to-face training. Increased workload, non-availability of medications, facility-level conflicts, and poor task shifting were identified challenges.

Conclusions: This initiative provides evidence of feasibility of implementing an NCD care package supported by mHealth training for health workers in PHCs, and the strong possibility of successful scale-up nationally.

Article Summary

Strengths and limitations of this study

- Pragmatic implementation of an mHealth training and support model based on an country context adapted (WHO PEN compatible) NCD package of care.

For peer review only - http://bmjopen.bmj.com/site/about/guidelines.xhtml

- Process evaluation of feasibility, implementation experience, challenges and patient satisfaction using quantitative and qualitative methods.
- Limitation in patient numbers attending facilities imposed by the COVID-19 pandemic and shortened pilot period leading to reduction in numbers screened, diagnosed, started on treatment and evaluated.
- Perception of the initiative as largely as a non-governmental organisation (NGO) pilot rather than an obligatory health systems strengthening initiative driven by the relevant authorities (State and Federal Ministries of Health)

Introduction

Non-Communicable Diseases (NCDs) are responsible for the deaths of 41 million people annually with an estimated 15 million of these deaths occurring in people between 30 and 69 years of age.[1] Among NCDs, the four top conditions that together account for more than 80% of all these premature deaths include cardiovascular diseases (17.9 million deaths annually), cancers (9 million), respiratory diseases (3.9 million), and diabetes (1.6 million). Almost three quarters of NCD linked deaths occur in low-and middle-income countries (LMICs). Fragile health systems experience challenges in controlling both NCDs and infectious diseases such as HIV/AIDS and tuberculosis (TB). The rapid global epidemiologic transition and rising rates of NCDs are also having negative effects on the health of Nigerians.

In Nigeria, NCDs are estimated to account for 29% of all deaths[2] with the probability of dying prematurely from NCDs being put at 20% by the World Health Organization (WHO).[3] Community-based surveys involving adults in Cross River Sate Nigeria have identified that approximately 7% of residents in Calabar had undiagnosed diabetes mellitus[4], with reported prevalence of hypertension and obesity being 42% and 18% respectively.[5] It is projected that by 2030, NCDs will be the leading cause of morbidity and mortality in Nigeria.[6,7] This rise in NCDs has been attributed to rapid population growth and urbanisation, limited access to healthy and nutritious food and physical inactivity.[8,9]

As with other low-middle income countries PHC facility delivery has been for acute communicable diseases such as malaria. Nurses and other health workers in PHC facilities were not trained to prevent, diagnose and manage NCDs.

The fragile Nigerian public health system is not well equipped to effectively manage and control NCDs.[10] Prior to now, NCD care was not prioritised by policy makers. For example, only 3.3% of the national health budget is devoted to mental health care with fewer than 150 mental health specialists serving Nigeria's estimated 206 million population.[11] In 2019, Nigeria's Federal Ministry of Health launched the National Multisectoral Action Plan for the Control and Prevention of NCDs[12], prioritising health systems strengthening at all levels of care towards universal health coverage (UHC). A major thrust of the plan was the integration of NCDs management into primary healthcare (PHC) services. Despite this, the

For peer review only - http://bmjopen.bmj.com/site/about/guidelines.xhtml

recently launched National task-shifting and task-sharing policy does not cover NCD prevention and control.[13] NCDs are not prioritised in health worker basic or post basic training, as in many other countries. So far, the PHC facilities in Nigeria have not been involved in programmatic/systematic care for NCDs (or other chronic diseases except for TB and HIV).

The Communicable Disease Health Service Delivery (COMDIS-HSD) programme has researched and developed care guideline processes to identify, diagnose and treat NCDs. These packages of care have been adapted and used at provincial scale in Pakistan, Swaziland[14] and elsewhere with higher rates of diagnosis and NCD control in the primary care facilities.[15-20] This NCD package has been scaled-up across the outpatient departments of all public hospitals and many health centres in the Punjab Province, resulting in the diagnosis and treatment of 540,000 people since 2017.[16-20] Similarly, COMDIS-HSD has researched, developed, trialled, and scaled to 71 rural health facilities in China covering 2.5 million people.[21]

The COMDIS-HSD NCD package has been adapted and adopted in Sierra Leone.[22] It has been also adapted by the Nigerian Federal Ministry of Health (FMoH) NCD technical working group with technical support from the Nuffield Leeds, United Kingdom (UK). The package is technically compatible with the WHO Package for Essential Noncommunicable (PEN) disease interventions and provides steps of medication and patient education (https://comdis-hsd.leeds.ac.uk/resources/ncd-care-package/). The PEN/COMDIS-HSD package has been piloted in its hard copy residential course format by the FMoH/NCD division in Abuja Federal Capital Territory. However, there is no funding for scaling -up these traditional in-class service courses for health workers at a national level.

By leveraging the high mobile phone penetration in Nigeria, we aimed to pilot the use of an innovative mobile health (mHealth) user-friendly view-through rate (VTR) mobile training application that can be used on any smart phone, tablet or computer, off-line or while connected to the internet. The guide and training modules were adapted for mHealth online delivery by Instrat Global Health Solutions (an indigenous technology company) with pre/ post tests and award of continuing professional development certificates. We hypothesised that mHealth NCD training and a support group for nurses should lead to improved knowledge and improved competencies in the screening/diagnosis, treatment initiation and optimal blood pressure control in patients at PHC level. Our overarching goal was to develop a workable and sustainable approach to support health workers in managing NCDs at PHC level which can be scaled-up across Nigeria.

Methods

Implementation package

The NCD desk guide, training modules, and treatment card record package was adapted from the World Health Organization PEN guidelines by our team (http://comdis-

For peer review only - http://bmjopen.bmj.com/site/about/guidelines.xhtml

hsd.leeds.ac.uk) in collaboration with Nigeria's WHO and the Federal Ministry of Health. Doctors are at the hospital level. Registered nurses are the more qualified health workers in primary healthcare (PHC) facilities in Nigeria but have not been trained to provide NCD prevention and treatment. The NCD package had been initially piloted in a conventional face to face training at a venue in the Federal capital territory of Abuja with WHO Nigeria funding. However, there is not funding for scale-up across the many states and thousands of PHC facilities of Nigeria. Online mHealth training and support has potential for large scale replication.

The case management desk guide(line) and training module package was adapted to be feasible in the country context guidelines in collaboration with Nigeria's WHO and Federal Ministry of Health. However, the mHealth training focused hypertension and/or diabetes and related obesity and lifestyle change as these are the most common NCD conditions in this context.

A logic model table was drawn up to include the inputs, desk guide(line), mHealth training modules, treatment card, nurse WhatsApp support group (Table 1) and the quarterly PHC clinic supervision visits. Included, in brackets were the research pre/post training scores, primary outcome (BP trend) and focus groups.

Research design

In this pilot, we adopted a mixed-methods approach to:

(i) Evaluate changes in NCD related knowledge through pre-post training assessments included with the online training.

(ii) Understand service delivery and implementation contextual factors through structured observations and focus group discussions involving the health workers following training.

(iii) Assess outcomes through record review of treatment cards for appropriateness of screening, diagnosis and initiation of treatment and changes in blood pressure etc.

The implementation of this pilot was guided by the proposed theory of change that recognises the various inputs and activities expected to generate patient related outcomes and health systems impact driven by policy change. The theory is underpinned by contextual factors such as the current organisation and resourcing and limitations of the health system, limitations placed on the scope of tasks performed by health workers at PHCs, availability of funding to train health workers and the opportunity to leverage mobile phone penetration. It was not possible to involve patients or the public in the design, or conduct, or reporting, or dissemination plans of our research.

For peer review only - http://bmjopen.bmj.com/site/about/guidelines.xhtml

Table 1:Logic model table showing intervention inputs, intervention process and intended practice change

Intervention inputs	Intervention process & actions	Intended		
		Practice change	Outputs	Health outcome
• Case management desk guide(line), including lifestyle counselling<br>• mHealth training of nurses on the deskguide package<br>• Clinical and implementation, nurse WhatsApp support group<br>• Supervision of care visits quarterly<br>• Digital BP apparatus, glucometer & strips<br>• Treatment card, used for clinical care [subsequently also as research data records]	• Screen/ diagnose*<br>• Prescribe anti-hypertensive<br>• Identify hypertension and/ or diabetes and treat<br>• Counsel for lifestyle modification<br>• Follow-up care<br><br>[Focus group discussion with nurses, month 5]	Provider practice:<br>• Screen, diagnose, treat, counsel, follow-up & report as per the case management desk guide(line)<br>Patients practice:<br>• Follow-up visits<br>• Treatment<br>• Lifestyle changes as counselled eg healthy eating, activity, smoking cessation	Patients are:<br>• Screened & diagnosed as per desk guide<br>• Prescribed right drug/ dose<br>• Counselled for life-style change<br>• Followed-up and treated – i.e., continuing care	• BP trend by visit [primary outcome] etc. hypertension and/or diabetes lifestyle and care recorded on the NCD card.<br><br>[Also pre/ post training scores]

Setting and participants

This pilot was carried out in a total of 19 PHC facilities in Cross River State, South-Eastern Nigeria over 12 months between February 2020 and January 2021. It involved 23 nurses drawn from selected sites in rural areas and peri-urban areas in Cross River State. Purposive sampling was used to choose the 19 PHC facilities and this was done in collaboration with the Cross River State Primary Health Care Development Agency. PHCs. It was spread across all the senatorial zones and local government areas with the aim of sampling from at least 10% of the just over 190 larger PHCs (staffing including at least one registered nurse) in Cross River State

For peer review only - http://bmjopen.bmj.com/site/about/guidelines.xhtml

Process

A one-day face-to-face orientation was organised in Calabar, the capital of Cross River State, in February 2020. This introduced the 23 health workers to the NCD training package, giving them hard copies of the desk guide, job aide, treatment cards and showed how to access and use the mHealth training modules for home self-study .

The mHealth modules were developed using the *MediXcel Lite* health technology platform which has been previously described.[23,24] The training modules consisting of video and text-based learning materials were created in English which was understood by all health workers. Several facilitation techniques were used by the trainers such as practical sessions, group discussions and case studies. All health workers were then expected to complete the eight NCD modules over a two-week period by accessing the NCD materials on their mobile phones and tablets. User logins were created for the health workers by the staff of InStrat to enable them download the App onto their android mobile phones. This App also tracked their progress with the NCD modules automatically. The maximum possible score was 100% with the pass score set at 70% for each module. Continuing professional development certificates were awarded to the health workers who completed the NCD modules.

Following this orientation and mHealth training, the health workers were expected to utilise the information contained in the modules to screen and identify patients presenting with NCDs to their various facilities. Treatment cards were distributed, and participants were instructed to upload monthly updates on the number of NCD cases seen in the various facilities via an electronic *Comm Care* App portal on the mobile electronic devices. Relevant clinical materials such sphygmomanometers, glucometers and test strips were provided for the facilities to facilitate the clinical assessment of clients with NCDs.

The health workers were provided with funds monthly to cover telecommunications fees. A community of practice (CoP) WhatsApp group was created for the health workers to facilitate peer-to-peer interactions, real time patient care support by clinicians and troubleshooting upload of data by IT personnel of the implementation team. The WhatsApp group also served as an avenue to provide clinical support and data recording support. . The health workers who uploaded their facility data onto the *Comm Care App* on time were openly commended as a form of non-financial motivation and as a reminder to others to do the same.

Supportive supervision was provided to the health workers in the form of weekly discussion WhatsApp phone calls and supplemented by quarterly facility clinician supervisory visits. The facility visits involved first-hand assessments of the service delivery contextual factors. During these trips, clinical mentoring was provided, and the health workers were observed providing care to patients using the clinical desk guide and treatment cards. Feedback was obtained from the health workers during these sessions.

For peer review only - http://bmjopen.bmj.com/site/about/guidelines.xhtml

Responding to the early implementation issues identified by the implementation team, a new module on integrating NCD management into daily activities of PHC facilities was developed and deployed on the training App.

Focus group discussions

In the 5th month from the start of implementation, focus group discussions (FGDs) were conducted as part of the research, with the health workers. Shorts message service invitation were sent to 24 participants who completed the intervention to share their perceptions of the digital training and experiences in focus group discussions. Focus groups[25] were the chosen method as they are useful for exploring perspectives and encouraging reflections on experiences. Twenty-three participants accepted the invitation. We conducted four focus group sessions over 10 days, with an average of 6 participants in each group (FG1 n=8, FG2 n=4, FG3 n=5, FG4 n=6). There were only two male participants, and they were part of the fourth group.

Each FGD was facilitated by 2 researchers experienced in conducting FGDs and an assistant. The research assistant coordinated the session schedules, recording and served as the official note-taker. Sessions were 1 – 2 hours in length and conducted face-to-face in the place convenient for participants. Open-ended questions, formulated by the team who have formal training in qualitative research were used to gain the best perspective and scope of individual responses on (1) perceptions of the use of m-Health training strategy, (2) experiences with the implementation of clinical guidelines (3) reflections on the adequacy of received supervisions, (4) reflections on potential scale-up of the intervention. Facilitator notes ensured uniformity among FGDs and kept each discussion on target. At each FDG, an opportunity was given to withdraw consent to participate and recording. Responses were recorded, and participants were assured their comments would be kept confidential and anonymous.

No formal sample size was calculated for this pilot study.

Ethical approval for the study was received from the Cross River State Health Research Ethics Committee CRSMOH/RP/REC/2019/173.

Data analysis

Quantitative data

The screening, diagnosis, treatment and follow-up care is specified in the NCD guide and this information is recorded on the treatment card. The record review was of all patients identified and initiated on care. The assessment was of quality care according to these guidelines and the completeness of recording of key clinical measurements of blood pressure (BP), body mass index (BMI), fasting blood sugar (FBS) and urine analysis (as per guideline for that disease) were metrics used to assess the effectiveness of the pilot project. We assessed the effect of the NCD mHealth training through the pre/post test results and

For peer review only - http://bmjopen.bmj.com/site/about/guidelines.xhtml

assessed operational issues that may have contributed to the results. The overall assessment was the degree to which the NCD mHealth training and care was feasible, sustainable, replicable, and treatment initiation quality was (as per guideline).

Data were imported and analysed in SPSS v23.[26] Descriptive analysis of quantitative data was carried out using means, standard deviations, frequencies, and percentages. Group differences were established using chi-square analysis. Descriptive trend analysis for the follow up rates in the systolic blood pressure and diastolic blood pressure changes in the hypertensive patients was conducted on the monthly basis. The paired t-test was used to compare the average change in systolic blood pressure and diastolic blood pressure levels at baseline and three-month follow up among the hypertensive patients. Hypothesis testing was two-tailed, at the 5% level.

Qualitative data

We used qualitative thematic analysis to identify prominent themes and patterns in the data and included a combination of deductive and emergent strategies. The facilitators debriefed immediately after each FGD. Two researchers (AO and SA) independently reviewed the data and came together to discuss emerging ideas. After the discussion, the researchers returned to the data to further review and confirm coding. This iterative process resulted in grouping the codes into three major themes: (1) perceptions with the mHealth trainings, (2) experiences with the implementation of NCD care packages, (3) potential issues with the intervention scale-up. Qualitative data were analysed using Dedoose software.[27]

The development of the research question and outcome measures in this initiative were directly informed by Nigerian patients' priorities, experience, and preferences. These priorities were factored into the design of this study. Patients were not directly involved in the recruitment to and conduct of the study but were beneficiaries of the NCD package of care we provided in the PHCs. We aim to disseminate the results of this initiative by conducting interactive sessions with the patients who continue to receive care for their NCDs in the PHCs in Cross River State.

Results

A total of 23 health workers from 19 primary health centres (PHCs) across the state were trained using the App. The average pre-test and post-test scores were 65(±12) and 87(±8) respectively with a statistically significant difference between these scores following a two-tailed t-test (p=0.000).

Since the project initiation, 414 (72.7%) community members served by 19 targeted centres were diagnosed with NCDs and 569 patients were served in total. Average age of males diagnosed with an NCD was 53 years (SD= 15) and for females 49 years (SD=14). Over three-quarters (n=432, 76%) of all cases and over half (n=315, 55%) of newly diagnosed cases

For peer review only - http://bmjopen.bmj.com/site/about/guidelines.xhtml

were hypertensive patients (newly diagnosed:110 male, 205 female, average age 52, SD=13) and 14% were diabetics (15 male, 35 female, average age 49, SD=14) (Table 1).

Fasting blood sugar checks were conducted on 215 of the community members with 47 (22%) of them falling within the prediabetes range of 5.6-6.9mmol/l and 82 (38%) falling above the diabetes threshold of >7.0mmol/l (Table 2). Proteinuria and/or glycosuria was identified in 14 of the 113 persons who had a urine analysis. In accordance with the NCD guideline, fasting blood glucose was only done if the person was overweight or had related symptoms, and urine analysis was only done as indicated.

Table 2. Characteristics of NCDs patients served by targeted PHC clinics n=569

**Characteristics**	**Male (n=206/36%)**	**Female (n=363/64%)**	**Total (n=569)**
Age (μ ± SD)	53.5±15.2	49.75±14.2	51.8 ±14.2
**NCD patients who attended facilities**			
Hypertension	155 (75.2%)	277 (76.3%)	432 (75.9%)
Diabetes	23 (11.2%)	47 (13.0%)	70 (12.3%)
Asthma/COPD	10 (4.9%)	17 (4.7%)	27 (4.7%)
Depression	1 (0.5%)	8 (2.2%)	9 (1.6%)
Sickle cell anaemia	6 (3.0%)	1(0.3%)	7 (1.2%)
Epilepsy	3 (1.5%)	3 (0.8%)	6 (1.1%)
Other NCD	8 (3.9%)	10 (2.8%)	18 (3.2%)
Total	206 (100%)	363 (100%)	569 (100%)
**New diagnosis of NCDs**			
Yes	145 (70.4%)	269 (74.2%)	414 (72.7%)
No	61 (29.6%)	94 (25.8%)	155 (27.3%)
Total	206 (100%)	363 (100%)	569 (100%)
**Check-up items**			
Height (μ ± SD)	1.56±0.14	1.56±0.11	1.56±0.12
Weight (μ ± SD)	67.4±12.6	68.5±12.4	68.1±12.5
**BMI**			

For peer review only - http://bmjopen.bmj.com/site/about/guidelines.xhtml

Normal 18.5-24	57 (27.7%)	114 (31.4%)	171 (30.0%)
Overweight 25-30	78 (37.9%)	124 (34.1%)	202 (35.5%)
Obese ≥30	62 (30.1%)	122 (33.6%)	184 (32.3%)
Total	206 (100%)	363 (100%)	569 (100%)
**Blood pressure (BP)**			
Normal BP 90/60mmHg-120/80mmHg	52 (25.2%)	73 (20.1%)	125 (22.7%)
High normal 130/85mmHg - 139/89mm Hg	21 (10.2%)	38 (10.5%)	59(10.4%)
High BP ≥140/90mmHg	133 (64.6%)	252(69.4%)	385 (67.7)
Total	206 (100%)	363 (100%)	569 (100%)
**Diagnostic tests**			
**Fasting blood sugar (FBS)**			
Normal reading <5.6mmol/l	30 (40.0%)	56 (40.0%)	86 (40.0%)
Prediabetes 5.6-6.9mmol/l	15 (20.0%)	32 (23%)	47 (21.9%)
Diabetes reading >7.0mmol/l	30 (40.0%)	52 (37.1%)	82 (38.1%)
Total	75(100%)	140 (100%)	215 (100%)
**Urine analysis**			
Normal	32(69.6%)	57(65.5%)	89 (66.9%)
Proteinuria and/or glycosuria§	14 (30.4%)	30(34.5%)	44 (33.1%)
Total recorded	46(100%)	87(100%)	133 (100%)

§Proteinuria- proteins in urine, glycosuria-sugar in urine. According to the NCD guideline, fasting blood glucose taken if overweight or have related symptoms, and urine analysis as indicated.

Gender distribution of newly diagnosed NCD cases (Figure 1) was not statistically different $\chi 2$ (6) =8.4, p=0.2. On average, 16 hypertensive and 3 diabetic patients were identified per facility (range 3-38 for hypertensive and 0-12 for diabetic patients).

Figure 1. Gender distribution of newly diagnosed NCD cases

All NCD patients (n=569) registered at targeted facilities received height (μ=1.56, SD=0.12) and weight measurements (μ=68.1, SD=12.47) at the first appointment. BMI was calculated

For peer review only - http://bmjopen.bmj.com/site/about/guidelines.xhtml

and recorded for all of these patients (μ=28.1, SD=5.85). Over forty percent of NCD patients (n=234, 41%) received an appointment reminder following the screening appointment.

Healthy lifestyle advice

In all, 356 (62.6 %) patients were given lifestyle advice such as reducing/ stopping cigarette smoking and alcohol, reducing weight, reducing salt intake, increasing physical activity/ exercise, reducing stress, medication adherence, healthy eating, and healthy living.. For these a motivational counselling strategy was used.

Primary outcome

Recordings on treatment cards indicated a positive significant improvement in blood pressure of hypertensive patients from visit 1 through to visit 3 (Figure 2). The systolic blood pressure for hypertensive patients steadily decreased from the screening (baseline) average of 160 to a visit 3 average of 135 mmHg. (Table 3). The average systolic blood pressure decreased 23 mmHg between the screening to follow-up visit 3 ($p<0.001$). A similar trend was registered in average decrease of 12mmHg in diastolic blood pressure from screening (baseline) of 96mmHg to follow-up visit 3 average of 79mmHg ($p<0.001$).

Figure 2: Trends in blood pressure change among hypertensive patients (n=428)

Table 3: Trend on blood pressure from visit 1 to visit 3 based on average of two measurements

Parameter	Value	Visit 1	Visit 2	Visit 3
Systolic BP (mmHg)	Valid values	416	227	96
	Mean (±SD)	159.9 (23.3)	143.3 (20.8)	139.5 (19.2)
	Range	90-270	99-218	80-180
	Values >140 (%)	60.7%	20.8%	7.5%
	Values >180 (%)	14.5%	2.1%	--
Diastolic BP (mmHg)	Valid values	416	227	96
	Mean (±SD)	95.9 (13.9)	85.1 (13.3)	81.6 (11.2)
	Range	50-152	80-130	50-120
	Values >90 (%)	49.1%	9.8%	2.8%
	Values >110 (%)	10%	2.1%	0.2%

Compliance with medication

The hypertensive patients were prescribed antihypertensive drugs from the following classes: (1) Angiotensin-converting enzyme inhibitors (lisinopril, captopril), (2) Calcium channel blockers (nifedipine, amlodipine), (3) Diuretics (furosemide, hydrochlorothiazide) which were all in line with recommendations provided in the NCD deskguide. Where

For peer review only - http://bmjopen.bmj.com/site/about/guidelines.xhtml

hydrochlorothiazide was unavailable, normoretic (i.e. amiloride hydrochloride 5mg and hydrochlorothiazide 50mg) was prescribed. The top three most often prescribed antihypertensive drugs were normoretic (50%), nifedipine (44%) and amlodipine (33%). Diabetic patients were mostly prescribed metformin, glibenclamide, or insulin injections.

Focus group discussions

Participants

Focus groups included 21 women and 2 men, between 31 to 60 years of age. All participants had post-secondary training in nursing. Some of the participants had prior experience of attending digital training. However, NCDs had not been prioritised in the health worker basic or post basic training the participant had previously received.

Perceptions of m-health training

Content

The mHealth training was stated to be a valuable experience. Participants were very appreciative of the knowledge they acquired about prevention and control of NCDs. They talked about improved confidence and an increased sense of empowerment in their ability to provide counselling on lifestyle behaviours and medication adherence. This positively influenced their internal sense of importance, consistent with improved self-efficacy.

> *"Some of these cases … before now I didn't know how to handle them but now I am very confident in handling the issues. The way and manner I am able to regulate clients' blood pressure are making me feel important" [FG1].*

> *"I will begin by appreciating the NCD project and people. Before now, we actually didn't know how to handle these cases but now we are very confident. We know the particular drugs to prescribe and the dosage". [FG2]*

Functionality

Participants perceive the functionality of m-health technology and ease of use as important. Multiple benefits of online learning were noted including the ability to learn at your own pace, the opportunity to return to the material when needed, using smart phones to access on the VTR the NCD deskguide and modules.

> *"We are upgraded. It makes the provider more educated, because you read at your pace, you don't stress yourself, anytime you come back you read and understand. So.. I think it was great."[FG1].*

> *"It was a nice one. Almost all the trainings we have attended have not been this type. It has made us to know how to use our phone in learning online and also searching*

For peer review only - http://bmjopen.bmj.com/site/about/guidelines.xhtml

for other things you would not have ordinarily done because of the workload where we find ourselves. It is a good one. Many times, we tell ourselves let's go online something is happening "[FG4].

Experiences with the implementation of NCD care package

Positive impact

Participants talked highly about positive changes the integrated care delivery brought to the communities they served. Provision of BP and blood glucose diagnostic equipment meant targeted diagnostic and curative services delivery at a point of access.

"The NCDP [the project] has made us have the necessary equipment to carry out blood pressure, diabetes diagnosis in my facility. This is a great achievement really. People come in now to say I want to check my blood pressure and sugar." [FG4]

When people come to our ANC, we have some of the test kits to use and we started testing. We admitted some people. When people come to visit them, they go back to the community to tell others. Presently, we have increased client flow. It is helping us to detect these NCDs". [FG4]

The provision of PHC facility services dramatically improved relationships with communities and encouraged follow-up visits. During outreaches, health workers were able to use the donated sphygmomanometers to measure blood pressure within the communities and refer people to the PHC facility.

"The relationship with the community as a result of the NCD is marvellous wherever we meet with these clients they greet one with respect and are open to me" [FG1]

"Giving the test that was not existing in the community before really help to mobilize the people. They feel more relaxed and confident to come to the facility".[FD4]

Participants described a variety of community education strategies to raise awareness of the availability of NCD services. Some strategies involved monetary reward for spreading the word about services by the town crier, and also use of local worship places and/or community organisation to engage with communities.

"…I have a relationship with the town announcer. Although when people come and take the test, we collect a token from them, and I use that to pay the town announcer. I type what I want him to announce. He reads through it and announces it to the people. From there people started coming". [FG4]

For peer review only - http://bmjopen.bmj.com/site/about/guidelines.xhtml

> *"This project has impacted on our knowledge, and we can confidently do health talk and create awareness about blood pressure, and other NCDs efficiently. My wish is.., I hope this project stays, honestly." [FG1]*

Challenges

Participants, however, also experienced, or perceived challenges to the implementation of care packages as extending across clinical and organisational levels.

Challenges at the clinical level

Factors identified as challenges at the clinical level included increased workload associated with (a) ensuring the continuity of NCD care delivery, and (2) filling in the treatment cards. Participants noted the NCD initiatives' success meant the increased flow of patients to facilities for check-ups and follow-up consultations. Although this has generally been perceived as a positive dynamic, the increase in workload was mentioned.

> *"NCDs increased our work. Before now, we see them and they go away, we don't bother to follow them up or they don't come back to us to maintain their BP. We need to maintain them to get their drugs or make sure their blood sugar level got controlled. Some of us, our facilities are easy to get to, so they can easily come. Even if you are resting, they want you to come and check them. When they heard that someone in the community died due to high blood pressure, that period a lot of people came for checking [FG4].*

Some participants also noted that filling the treatment cards and registers was time-consuming and often discouraged their use. Content reduction was a proposed solution to address this.

> *"Ordinarily we have few questions to capture from clients but the NCD has widened a lot of information that needs to be captured, looking at this with other registers in the facility too has made the workload high, and again there are series of tests that also needs to be run on an individual basis." [FG1].*

Technical glitches in the application and internet connection were also noted resulting in issues with uploading data into the central system. This resulted in double entry of data which was perceived as a double burden on staff. Some participants also recognised that supervision and monitoring provided by staff of the initiative helped them navigate many challenges along the way.

Challenges at the organisational level

For peer review only - http://bmjopen.bmj.com/site/about/guidelines.xhtml

The presence of professional support or opposition was highlighted as a considerable factor for the successful integration of the care packages into daily routine. Participants talked in length about conflict of interest between the focal person responsible for NCD care and PHC facility in-charge. Examples of interference from *person-in-charge* into modes of care delivery, equipment storage and use as well as generated income distribution were noted.

> *"The thing is really disturbing because the in-charge kept telling you that the equipment is not for you and should be kept in the laboratory, giving unnecessary orders before you can make use of it. It pissed me off a lot." [FD1].*

Some participants, on the other hand, talked positively about PHC facility in-charge and the support they received. They also acknowledged how some managers actively "champion" the awareness-raising activities amongst communities, and how this considerably increased the flow of patients to facilities.

> *"The new in charge has leadership skills. He went to the community heads and introduced himself, he went to churches. He will encourage sick people to always come and test. A lady died and it was discovered her blood pressure was high. So, they announced in all the churches that people should go and check their blood pressure and sugar level. This really helped us. [FG4]*

Although many participants talked in terms of conflict, in general, negotiation, avoiding confrontations and/ or for managers undergoing training on NCD care alongside junior staff were preferred approaches to resolving the problems.

> *"I believe they should train them. Since we have this knowledge, they know some of the drugs, but they don't know the right time to administer. If they are exposed to this training and they are told the stages and when to prescribe, they will know what to do. For example, in my facility my in-charge prescribed stage II drugs instead of stage I, I had to correct him. If they know, they will do better." [FG4]*

Participants also noted negative changes in the spirit of collegiality and the need to ensure that *"the focal persons"* have the expertise to manage NCD cases.

Potential issues with the intervention scale-up

Participants talked about pressing issues in a healthcare system that potentially can have a negative impact on the scale-up of the intervention. The most often mentioned challenges identified were health personnel shortage (especially nurses), lack of knowledge on NCD management and control, lack of essential diagnostic equipment and medication necessary

For peer review only - http://bmjopen.bmj.com/site/about/guidelines.xhtml

for effective NCD care delivery. The intervention included to train one nurse (a 3-year trained registered nurse).

Potential key solutions for ensuring the sustainability and potential scale-up of care packages at a national level were (a) targeted training of other staff on NCD prevention and management, and (b) task-shifting activities for NCD care. Participants also noted that staff at primary care level lacked basic knowledge on NCD care and if left at their own disposal many essential diagnostic tests will be missed. Therefore, they will be willing to provide training to junior staff to ensure effective delivery of services.

> *"Yes…, because some things, if left for the facility to carry on, will not be effective, but with a focal person she will ensure things are in order, since they know they will be held responsible" [FG1].*

> *"Yes, like in my facility I am the only staff I will appreciate if another staff is sent here so that we can have more hands, and I will give the person on the job training or some kind of step-down training to enable whoever is coming to render the services. It will be bad if this NCDP [the NCD project] stops, before now we have had cases where somebody will just fall while walking. The next thing you hear is people will be saying he is a ghost, not knowing is the effect of high blood pressure. So,…. I really don't want this NCD thing to just fade like that, I don't know how to put it really" [FGD2].*

Discussion

Main findings

In this mixed-methods pragmatic implementation of health worker training using an mHealth platform complemented by clinical supervision visits and a community of practice forum, we have demonstrated the feasibility of delivering training remotely leading to improvement in knowledge and skills in NCD screening, diagnosis, and treatment. In addition, we have documented nurses' experiences, perspectives, and challenges as well as satisfaction of clients with the care provided which involved shifting of tasks to nurses. This cohort of patients was found to have improved trends on average control of blood pressure. There was a statistically significant reduction on both systolic and diastolic blood pressures between the first and third visits among hypertensive patients. Nonetheless, the results do not prove a causal relationship between the mhealth training intervention and the resultant trend in blood pressure. Diuretics and calcium channel blockers (nifedipine and amlodipine) were the most commonly prescribed antihypertensives with metformin and glibenclamide being the most commonly prescribed therapies for diabetes mellitus. These prescribing practices were all in line with the recommendations within the NCD guide.

For peer review only - http://bmjopen.bmj.com/site/about/guidelines.xhtml

Health education/counselling was given to about two-thirds of patients on all visits. The process indicators demonstrate the ability of nurses in PHC clinics to successfully deliver the key components of NCD care. The NCD guide was designed for qualified staff at the level of a 3-year trained registered nurse. The deployment of an mHealth platform rather than a face-to-face classroom style model to deliver the training has been shown to be feasible and effective. This is seen in the improved knowledge scores upon completion of the modules and assessment of skills seen in ability to initiate care for patients with NCDs. The mHealth NCD training was perceived as a valuable experience with the health workers identifying multiple benefits of online learning and the integrated NCD care delivery package. This is important as there is not funding sufficient for face to face training (including per diems and venue cost) for nation-wide scale up of NCD training and supervision across Nigeria, as in other low-middle income countries - there is no 'Global Fund for NCDs, as there is for AIDS, TB, Malaria).

Challenges to the smooth rollout of this NCD package were the increased workload it generated, the technical problems with internet connection and difficultly in securing the support of health centre in-charges for the project. Remote and periodic onsite supervision by local doctors and the provision of the materials (i.e BP equipment, glucometres etc) that were lacking at the facilities were additional strategy adopted to support this initiative. The availability of these se materials contributed tothe overall success of the pilot. Therefore, such extra provisions will need to factored into the eventual scale up of this NCD package. Considerations such as appropriate diagnosis, quality of care and linkage to continuing care will need to be further addressed using a health systems framework that deals with these challenges more comprehensively.[28]

While the pilot appears to be successful, the sustainability and replicability of this model of training and care initiation will depend on addressing several health system barriers such as appropriate staffing (not all facilities have a nurse), provision of medicines and diagnostic equipment, improving on the governance system at facility level and formally task shifting to nurses the NCD care and prescribing. In addition, the reference to the initiative as a 'project' by nurses implies a risk to sustainability, if the NCD initiative is not wholly adopted by the state health management. Despite its implementation through the routine system by a local medical NGO, the perception of it as a 'project' may undermine the long-term sustainability at facility level.This mixed methods research adds to the evidence base of similar NCD interventions in resource-limited settings. Using a similar shared care guide for management of diabetes and hypertension care in Eswatini (southern Africa), nurses were able to successfully deliver diabetes and hypertension care with improvements in physiological parameters, though continued training and supervision were required to consolidate change.[14] A similar NCD guideline and training COMDIS-HSD partner initiative in Pakistan was found to be effective in trials and was scaled across the Punjab Province of 110 million population.[16-20] This Nigeria pilot adds evidence for the potential in a West African

For peer review only - http://bmjopen.bmj.com/site/about/guidelines.xhtml

setting and for mHealth as an alternative to expensive residential courses, which is a major constraint to NCD care scale-up.

Limitations

The observational nature of our study precludes from drawing decisive conclusions as to whether care under the new NCD care model is better or worse than the alternative conventional residential training practiced.

The emergence of COVID-19 and imposed public health restrictions substantially limited the number of individuals attending the pilot PHCs to be screened and diagnosed over the equally short period of ten months. Community mobilization was conducted by the trained nurses to help overcome some social barriers to accessing care that came in the wake of the COVID-19 pandemic. Some of the health facilities were vandalised during the nationwide protests that engulfed Nigeria in October 2020, and this also had a negative impact on the NCD care services. In addition, the pragmatic nature of our initiative precluded the provision of medicines, which may have led to an increase in NCD patients observed. Furthermore, given that many NGOs are operating in fragile health environments, pilot initiatives may not be subsequently implemented by ministries of health.

Conclusion

This pilot initiative provides evidence of feasibility of implementing an NCD care package supported by mHealth training and support for health workers in PHCs as indicated by improvement in BP measurements in follow-up visits. These results support the implementation of such a package of care at scale within the Nigerian context.

Acknowledgements: We appreciate the NCD division of the FMOH, Nigeria, Cross River State Ministry of Health and Primary Healthcare Development Agency as well as nurses and facility heads who participated in the pilot. We are grateful to all the patients who accepted to be managed using the NCD guides in the various health facilities in Cross River State Nigeria. We are thankful to Mr Tijesu Ojumu of the Nigeria Centre for Disease Control for designing the conceptual framework. Our sincere gratitude goes to the NIHR RUHF project of QMU Edinburgh for funding this initiative.

Contributorship statement: AO, EE, ObO and JW conceived the study. AO, EE, ObO, OO and CO were involved in the implementation of the programme. EE, ObO, OO and CO handled the data entry. SA, AO, ObO, JW and OO did the data cleaning and analysis. AO, SA, EE, JW and ObO wrote the first draft. All authors were involved in the reviewing and editing of this article and gave final approval of the version to be published. AO is the guarantor of the article.

For peer review only - http://bmjopen.bmj.com/site/about/guidelines.xhtml

Competing interests: None declared.

Funding: This study/project is funded by the UK National Institute for Health Research (NIHR) [NIHR Global Health Research programme (project reference 16/136/100)/ NIHR Research Unit on Health in Situations of Fragility]. The views expressed are those of the author(s) and not necessarily those of the NIHR or the Department of Health and Social Care. The funder played no role in the study design, data collection, analysis, interpretation, or in writing the manuscript.

Data sharing statement: Data generated by your research that supports your article will be made openly and publicly available upon publication of your article.

Patient consent for publication: not required

Ethics approval: This study was performed in line with the principles of the Declaration of Helsinki. The study received the approval of the Health Research and Ethics Committee of the Cross River State Ministry of Health Nigeria (Number/ID: CRSMOH/RP/REC/2019/173). Participation was voluntary.

Data availability statement: No data are available.

Supplemental material: None supplied.

Patient and Public Involvement: The development of the research question and outcome measures in this initiative were directly informed by Nigerian patients' priorities, experience, and preferences. These priorities were factored into the design of this study. Patients were not directly involved in the recruitment to and conduct of the study but were beneficiaries of the NCD package of care we provided in the PHCs. We aim to disseminate the results of this initiative by conducting interactive sessions with the patients who continue to receive care for their NCDs in the PHCs in Cross River State.

References

1. World Health Organization 2021. Noncommunicable diseases – key facts. Available: https://www.who.int/news-room/fact-sheets/detail/noncommunicable-diseases
2. World Health Organization - Noncommunicable Diseases (NCD) Country Profiles. Available: https://www.who.int/nmh/countries/nga_en.pdf?ua=1
3. World Health Organization [WHO]. Global health observatory data 2018: Premature NCD deaths. Available: https://www.who.int/gho/ncd/mortality_morbidity/ncd_premature_text/en/
4. Enang OE, Otu AA, Essien OE, *et al*. Prevalence of dysglycemia in Calabar: a cross-sectional observational study among residents of Calabar, Nigeria. *BMJ Open Diabetes Res Care* 2014; 2(1):e000032. doi: 10.1136/bmjdrc-2014-000032

For peer review only - http://bmjopen.bmj.com/site/about/guidelines.xhtml

5. Effa E, Onwusaka, O, Ekrikpo U, *et al*. Community based screening for risk factors of chronic kidney disease in Cross River State. *Kidney International Reports* 2020; 5(3):S58-S59.
6. Maiyaki M, Garbati M. The burden of non-communicable diseases in Nigeria; In the context of globalization. *Ann Afr Med* 2014;13:1–10.
7. World Health Organization. Noncommunicable diseases: the slow motion disaster. Geneva: WHO; 2017.
8. Crush J, Frayne B, McLachlan M. "Rapid Urbanization and the Nutrition Transition in Southern African." Urban Food Security Series No. 7. Queen's University and AFSUN: Kingston and Cape Town. 2011.
9. Maher D, Sekajugo J. Research on health transition in Africa: time for action. *Health Res Policy Syst* 2011; 9:1–4.
10. Green A. Country in focus: the growing burden of NCDs in Nigeria. *The Lancet Diabetes and Endocrinology* 2016; 4(1): 23-24
11. Ajayi T. What is Mental Health to an African? Available: https://www.linkedin.com/pulse/what-mental-health-african-tomiwa-ajayi
12. National multi-sectoral action plan for the prevention and control of non-communicable diseases (2019 – 2025). Available: https://www.iccp-portal.org/system/files/plans/NCDs_Multisectoral_Action_Plan.pdf
13. Federal Ministry of Health Nigeria 2014. Task-shifting and task-sharing policy for essential health care services in Nigeria. Available: file:///C:/Users/akaninyene.otu/Downloads/TSTS.pdf
14. Sharp A, Riches N, Mims A, *et al*. Decentralising NCD management in rural southern Africa: evaluation of a pilot implementation study. *BMC Public Health* 2020*;* 20:44
15. Walley J, Khan MA, Witter S, et al. Embedded health service development and research: why and how to do it (a ten-stage guide). *H Research Policy* & *Systems*. 2018; D-17-*00256R1*
16. Khan MA, Khan N, Walley JD, *et al*. Enhanced hypertension care through private clinics in Pakistan: a cluster randomised trial. *BJGP Open*. 2019; 3(1):bjgpopen18X101617.
17. Khan MA, Walley JD, Khan N, *et al*. Effectiveness of an integrated diabetes care package at primary healthcare facilities: a cluster randomised trial in Pakistan. *BJGP Open.* 2018;2(4):bjgpopen18X101618. doi:10.3399/bjgpopen18X101618
18. Khan N, Khan M, Walley J, et al. A process evaluation of integrated diabetes management at primary health care facilities in Pakistan. *BJGP Open*. 2018;2(4):bjgpopen18X101612
19. Khan MA, Khan N, Walley J, *et al*. How effective is it to deliver integrated COPD care at public healthcare facilities: a cluster randomised trial in Pakistan? BJGP Open 2019; 3 (1). bjgpopen18X101634. ISSN 2398-3795

For peer review only - http://bmjopen.bmj.com/site/about/guidelines.xhtml

20. Khan MA, Khan MA, Walley J, *et al*. Feasibility to deliver integrated COPD-Asthma care at primary and secondary level public healthcare facilities in Pakistan: a process evaluation. BJGP Open 2019; DOI: 10.3399/bjgpopen18X101632
21. Wei X, Walley JD, Zhang Z, et al. Implementation of a comprehensive intervention for patients at high risk of cardiovascular disease in rural China: A pragmatic cluster randomized controlled trial. *PloS One* 2017; 12 (8). e0183169.
22. Witter S, Zou G, Diaconu, K. et al. Opportunities and challenges for delivering non-communicable disease management and services in fragile and post-conflict settings: perceptions of policy-makers and health providers in Sierra Leone*. Confl Health* 2020; 14, 3. https://doi.org/10.1186/s13031-019-0248-3
23. Otu, A, Okuzu, O, Ebenso, B, et al. Introduction of mobile health tools to support COVID-19 training and surveillance in Ogun State Nigeria. *Front Sustain Cities* 2021; 3: 10.
24. Otu A, Okuzu O, Effa E, et al. Training health workers at scale in Nigeria to fight COVID-19 using the InStrat COVID-19 tutorial app: an e-health interventional study. *Therapeutic Advances in Infectious Disease* 2021. doi:10.1177/20499361211040704
25. Willis K, Green J, Daly J, *et al*. Perils and possibilities: achieving best evidence from focus groups in public health research. *Aust N Z J Public Health.* 2009;33(2):131–6
26. IBM Corp. Released 2015. IBM SPSS Statistics for Windows, Version 23.0. Armonk, NY: IBM Corp.
27. Dedoose Version 9.0.17, web application for managing, analyzing, and presenting qualitative and mixed method research data (2021). Los Angeles, CA: SocioCultural Research Consultants, LLC www.dedoose.com.
28. Bloomfield GS, Vedanthan R, Vasudevan L et al. Mobile health for non-communicable diseases in Sub-Saharan Africa: a systematic review of the literature and strategic framework for research. Global Health. 2014 Jun 13;10:49. doi: 10.1186/1744-8603-10-49.

For peer review only - http://bmjopen.bmj.com/site/about/guidelines.xhtml

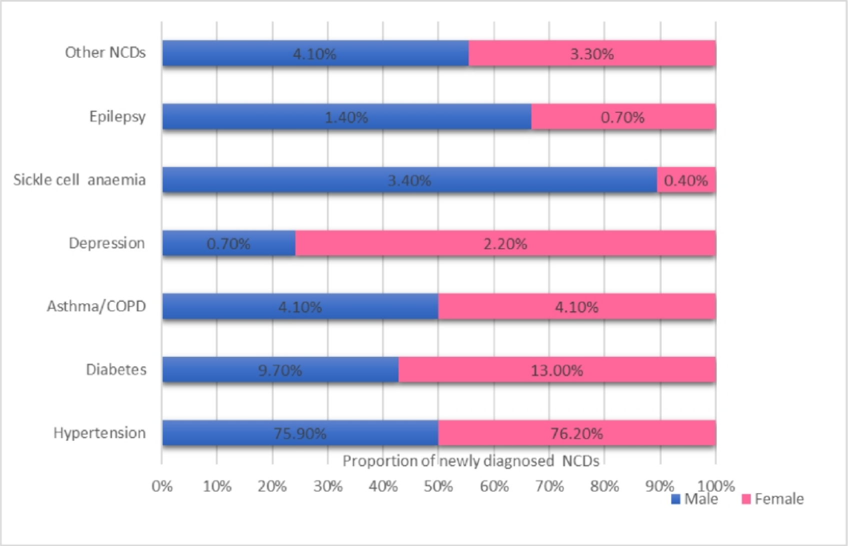

Figure 1. Gender distribution of newly diagnosed NCD cases

338x190mm (200 x 200 DPI)

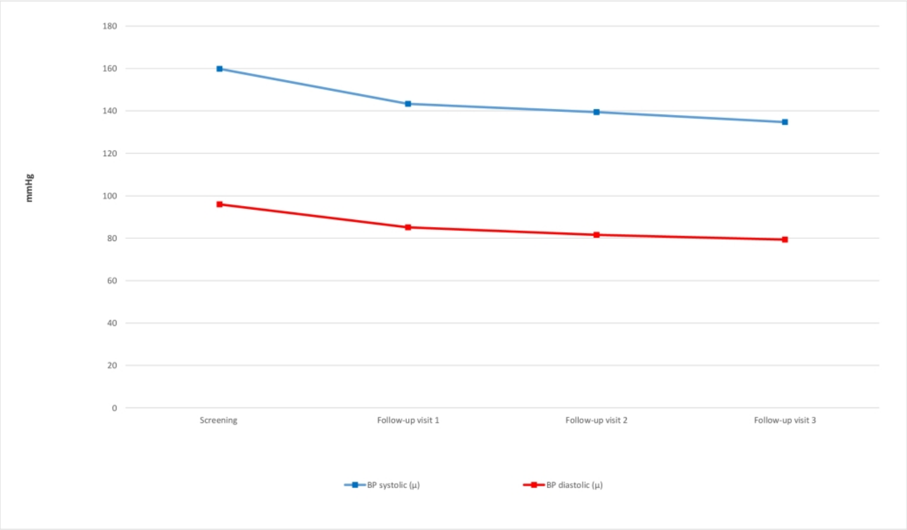

Figure 2. Trends in blood pressure change among hypertensive patients (n=428)

338x190mm (200 x 200 DPI)

Standards for Reporting Qualitative Research (SRQR)*

http://www.equator-network.org/reporting-guidelines/srqr/

Page/line no(s).

Title and abstract

**Title** - Concise description of the nature and topic of the study Identifying the study as qualitative or indicating the approach (e.g., ethnography, grounded theory) or data collection methods (e.g., interview, focus group) is recommended	Page 1, line 2-3
**Abstract** - Summary of key elements of the study using the abstract format of the intended publication; typically includes background, purpose, methods, results, and conclusions	Page 2, line 33-66

Introduction

**Problem formulation** - Description and significance of the problem/phenomenon studied; review of relevant theory and empirical work; problem statement	Page 4, line 113-133
**Purpose or research question** - Purpose of the study and specific objectives or questions	Page 4, line 213-130

Methods

**Qualitative approach and research paradigm** - Qualitative approach (e.g., ethnography, grounded theory, case study, phenomenology, narrative research) and guiding theory if appropriate; identifying the research paradigm (e.g., postpositivist, constructivist/ interpretivist) is also recommended; rationale**	Page 6 and 7, Line197-221
**Researcher characteristics and reflexivity** - Researchers' characteristics that may influence the research, including personal attributes, qualifications/experience, relationship with participants, assumptions, and/or presuppositions; potential or actual interaction between researchers' characteristics and the research questions, approach, methods, results, and/or transferability	Page 6, line 205 -211
**Context** - Setting/site and salient contextual factors; rationale**	Page 4, line 133-145
**Sampling strategy** - How and why research participants, documents, or events were selected; criteria for deciding when no further sampling was necessary (e.g., sampling saturation); rationale**	Page 7, line 245 - 252
**Ethical issues pertaining to human subjects** - Documentation of approval by an appropriate ethics review board and participant consent, or explanation for lack thereof; other confidentiality and data security issues	Page 20, line 656-659
**Data collection methods** - Types of data collected; details of data collection procedures including (as appropriate) start and stop dates of data collection and analysis, iterative process, triangulation of sources/methods, and modification of procedures in response to evolving study findings; rationale**	Page 7, line 245-252

For peer review only - http://bmjopen.bmj.com/site/about/guidelines.xhtml

**Data collection instruments and technologies** - Description of instruments (e.g., interview guides, questionnaires) and devices (e.g., audio recorders) used for data collection; if/how the instrument(s) changed over the course of the study	Page 7, line 245-252
**Units of study** - Number and relevant characteristics of participants, documents, or events included in the study; level of participation (could be reported in results)	Page 12, line 322-325
**Data processing** - Methods for processing data prior to and during analysis, including transcription, data entry, data management and security, verification of data integrity, data coding, and anonymization/de-identification of excerpts	Page 12, line 322-325
**Data analysis** - Process by which inferences, themes, etc., were identified and developed, including the researchers involved in data analysis; usually references a specific paradigm or approach; rationale**	Page 12, line 322-325
**Techniques to enhance trustworthiness** - Techniques to enhance trustworthiness and credibility of data analysis (e.g., member checking, audit trail, triangulation); rationale**	Page 12, line 322-325

Results/findings

**Synthesis and interpretation** - Main findings (e.g., interpretations, inferences, and themes); might include development of a theory or model, or integration with prior research or theory	Page 12-16
**Links to empirical data** - Evidence (e.g., quotes, field notes, text excerpts, photographs) to substantiate analytic findings	Page 17, line 540-579

Discussion

**Integration with prior work, implications, transferability, and contribution(s) to the field** - Short summary of main findings; explanation of how findings and conclusions connect to, support, elaborate on, or challenge conclusions of earlier scholarship; discussion of scope of application/generalizability; identification of unique contribution(s) to scholarship in a discipline or field	Page 3, line 62-72
**Limitations** - Trustworthiness and limitations of findings	Page 19, line 551-559

Other

**Conflicts of interest** - Potential sources of influence or perceived influence on study conduct and conclusions; how these were managed	Page 20, line 654
**Funding** - Sources of funding and other support; role of funders in data collection, interpretation, and reporting	Page 20, line 647-653

*The authors created the SRQR by searching the literature to identify guidelines, reporting standards, and critical appraisal criteria for qualitative research; reviewing the reference lists of retrieved sources; and contacting experts to gain feedback. The SRQR aims to improve the transparency of all aspects of qualitative research by providing clear standards for reporting qualitative research.

For peer review only

For peer review only - http://bmjopen.bmj.com/site/about/guidelines.xhtml

**The rationale should briefly discuss the justification for choosing that theory, approach, method, or technique rather than other options available, the assumptions and limitations implicit in those choices, and how those choices influence study conclusions and transferability. As appropriate, the rationale for several items might be discussed together.

Reference:

O'Brien BC, Harris IB, Beckman TJ, Reed DA, Cook DA. **Standards for reporting qualitative research: a synthesis of recommendations.** *Academic Medicine*, Vol. 89, No. 9 / Sept 2014 DOI: 10.1097/ACM.0000000000000388

For peer review only

BMJ Open

mHealth guideline training for non-communicable diseases in primary care facilities in Nigeria: a mixed methods pilot study

Journal:	*BMJ Open*
Manuscript ID	bmjopen-2021-060304.R2
Article Type:	Original research
Date Submitted by the Author:	29-Jun-2022
Complete List of Authors:	Otu, Akaninyene; University of Calabar, Internal Medicine<br>Effa, Emmanuel; University of Calabar, Renal Unit, Department of Internal Medicine; University of Cape Town, Division of Nephrology and Hypertension<br>Onwusaka, Obiageli; University of Calabar<br>Omoyele, Chiamaka; Federal Ministry of Health<br>Arakelyan, Stella; Queen Margaret University Institute for International Health and Development<br>Okuzu, Okey; Instrat Global Health Solutions<br>Walley, John; University of Leeds, Nuffield Centre for International Health and Development, Leeds Institute of Health Sciences
**Primary Subject Heading**:	Public health
Secondary Subject Heading:	Global health, Public health
Keywords:	PUBLIC HEALTH, Health informatics < BIOTECHNOLOGY & BIOINFORMATICS, EDUCATION & TRAINING (see Medical Education & Training)

SCHOLARONE™
Manuscripts

I, the Submitting Author has the right to grant and does grant on behalf of all authors of the Work (as defined in the below author licence), an exclusive licence and/or a non-exclusive licence for contributions from authors who are: i) UK Crown employees; ii) where BMJ has agreed a CC-BY licence shall apply, and/or iii) in accordance with the terms applicable for US Federal Government officers or employees acting as part of their official duties; on a worldwide, perpetual, irrevocable, royalty-free basis to BMJ Publishing Group Ltd ("BMJ") its licensees and where the relevant Journal is co-owned by BMJ to the co-owners of the Journal, to publish the Work in this journal and any other BMJ products and to exploit all rights, as set out in our licence.

The Submitting Author accepts and understands that any supply made under these terms is made by BMJ to the Submitting Author unless you are acting as an employee on behalf of your employer or a postgraduate student of an affiliated institution which is paying any applicable article publishing charge ("APC") for Open Access articles. Where the Submitting Author wishes to make the Work available on an Open Access basis (and intends to pay the relevant APC), the terms of reuse of such Open Access shall be governed by a Creative Commons licence – details of these licences and which Creative Commons licence will apply to this Work are set out in our licence referred to above.

Other than as permitted in any relevant BMJ Author's Self Archiving Policies, I confirm this Work has not been accepted for publication elsewhere, is not being considered for publication elsewhere and does not duplicate material already published. I confirm all authors consent to publication of this Work and authorise the granting of this licence.

For peer review only

Title:

mHealth guideline training for non-communicable diseases in primary care facilities in Nigeria: a mixed methods pilot study

Akaninyene Otu[1,2], Emmanuel Effa[1,2], Obiageli Onwusaka[2,3], Chiamaka Omoyele[4], Stella Arakelyan[5], Okey Okuzu[6], John Walley[7]

1. Department of Internal Medicine, College of Medical Sciences, University of Calabar, Calabar, Cross River State, Nigeria
2. Foundation for Healthcare Innovation and Development, Calabar, Cross River State, Nigeria.
3. Department of Public Health Faculty of Allied Medical Sciences, College of Medical Sciences, University of Calabar, Calabar, Nigeria.
4. Monitoring & Evaluation Branch, Non-Communicable Diseases Division, Federal Ministry of Health, Abuja, Nigeria
5. NIHR Research Unit on Health in Fragility, Institute for Global Health and Development, Queen Margaret University, Edinburgh, Scotland
6. InStrat LLC, Montclair, NJ USA
7. Nuffield Centre for International Health and Development, Leeds Institute of Health Sciences, University of Leeds, Leeds, UK

Corresponding Author:

Dr Akaninyene Otu

Department of Internal Medicine, College of Medical Sciences

University of Calabar, Calabar, Cross River State, Nigeria

E mail: akanotu@yahoo.com

For peer review only

Abstract

Objective: To pilot the use of a scalable innovative mobile health (mHealth) non-communicable diseases (NCDs) training application for nurses at primary care level.

Design: Mixed methods pilot of an mHealth training on NCD care for nurses at primary health care (PHC) facilities. We provide a descriptive analysis of mHealth training test scores, with trend analysis of blood pressure control using paired t-test for quantitative data and thematic analysis for qualitative data.

Setting: Primary Healthcare facilities in rural and urban communities in Cross River State, south eastern Nigeria. NCDs were not part of routine training previously. As in most low-middle income settings, funding for scale-up using conventional classroom in-service training for NCDs is not available in Nigeria, and onsite supervision poses challenges

Participants: Twenty-three nurses in 19 PHC facilities

Intervention: A self-paced mHealth training module on an NCD desk guide was adapted to be applicable within the the Nigerian context in collaboration with the Federal Ministry of Health. Training focused on hypertension, diabetes, and sickle cell disease was delivered via android tablet devices supplemented by quarterly onsite supervision and group support via WhatsApp. The training was evaluated with pre- and post-course tests structured observations and focus group discussions. This was an implementation pilot assessing the feasibility and potential effectiveness of mHealth training and NCD and primary care delivery.

Results: Nurses who received mHealth training recorded a statistically significant difference ($p<0.001$) in average pre-and post-test training scores of 65.2 (±12.2) and 86.5(±7.9) respectively. Recordings on treatment cards indicated appropriate diagnosis and follow-up of hypertensive patients with significant improvements in systolic blood pressure ($t=5.09$, $p<0.001$) and diastolic blood pressure ($t=5.07$, $p<0.001$). The mHealth nurse training and WhatsApp support groups were perceived as a valuable experience and obviated the need for face-to-face training. Increased workload, non-availability of medications, facility-level conflicts, and poor task shifting were identified challenges.

Conclusions: This initiative provides evidence of feasibility of implementing an NCD care package supported by mHealth training for health workers in PHCs, and the strong possibility of successful scale-up nationally.

Article Summary

Strengths and limitations of this study

- Pragmatic implementation of an mHealth training and support model based on a country specific (WHO PEN compatible) NCD package of care.

For peer review only - http://bmjopen.bmj.com/site/about/guidelines.xhtml

- Successful process evaluation of feasibility, implementation experience, challenges and patient satisfaction using quantitative and qualitative methods.
- Limitation in patient numbers attending facilities imposed by the COVID-19 pandemic and shortened pilot period leading to reduction in numbers screened, diagnosed, started on treatment and evaluated.
- Perception of the initiative as largely as a non-governmental organisation (NGO) pilot rather than an obligatory health systems strengthening initiative driven by the relevant authorities (State and Federal Ministries of Health)

Introduction

Non-Communicable Diseases (NCDs) are responsible for the deaths of 41 million people annually with an estimated 15 million of these deaths occurring in people between 30 and 69 years of age.[1] Among NCDs, the four top conditions that together account for more than 80% of all these premature deaths include cardiovascular diseases (17.9 million deaths annually), cancers (9 million), respiratory diseases (3.9 million), and diabetes (1.6 million). Almost three quarters of NCD linked deaths occur in low-and middle-income countries (LMICs). Fragile health systems experience challenges in controlling both NCDs and infectious diseases such as HIV/AIDS and tuberculosis (TB). The rapid global epidemiologic transition and rising rates of NCDs are also having negative effects on the health of Nigerians.

In Nigeria, NCDs are estimated to account for 29% of all deaths[2] with the probability of dying prematurely from NCDs being put at 20% by the World Health Organization (WHO).[3] Community-based surveys involving adults in Cross River Sate Nigeria have identified that approximately 7% of residents in Calabar had undiagnosed diabetes mellitus[4], with reported prevalence of hypertension and obesity being 42% and 18% respectively.[5] It is projected that by 2030, NCDs will be the leading cause of morbidity and mortality in Nigeria.[6,7] This rise in NCDs has been attributed to rapid population growth and urbanisation, limited access to healthy and nutritious food and physical inactivity.[8,9]

As with other low-middle income countries PHC facility delivery has been for acute communicable diseases such as malaria. Nurses and other health workers in PHC facilities were not trained to prevent, diagnose and manage NCDs.

The fragile Nigerian public health system is not well equipped to effectively manage and control NCDs.[10] Prior to now, NCD care was not prioritised by policy makers. For example, only 3.3% of the national health budget is devoted to mental health care with fewer than 150 mental health specialists serving Nigeria's estimated 206 million population.[11] In 2019, Nigeria's Federal Ministry of Health launched the National Multisectoral Action Plan for the Control and Prevention of NCDs[12], prioritising health systems strengthening at all levels of care towards universal health coverage (UHC). A major thrust of the plan was the integration of NCDs management into primary healthcare (PHC) services. Despite this, the

For peer review only - http://bmjopen.bmj.com/site/about/guidelines.xhtml

recently launched National task-shifting and task-sharing policy does not cover NCD prevention and control.[13] NCDs are not prioritised in health worker basic or post basic training, as in many other countries. So far, the PHC facilities in Nigeria have not been involved in programmatic/systematic care for NCDs (or other chronic diseases except for TB and HIV).

The Communicable Disease Health Service Delivery (COMDIS-HSD) programme has researched and developed care guideline processes to identify, diagnose and treat NCDs. These packages of care have been adapted and used at provincial scale in Pakistan, Swaziland[14] and elsewhere with higher rates of diagnosis and NCD control in the primary care facilities.[15-20] This NCD package has been scaled-up across the outpatient departments of all public hospitals and many health centres in the Punjab Province, resulting in the diagnosis and treatment of 540,000 people since 2017.[16-20] Similarly, COMDIS-HSD has researched, developed, trialled, and scaled to 71 rural health facilities in China covering 2.5 million people.[21]

The COMDIS-HSD NCD package has been adapted and adopted in Sierra Leone.[22] It has been also adapted by the Nigerian Federal Ministry of Health (FMoH) NCD technical working group with technical support from the Nuffield Leeds, United Kingdom (UK). The package is technically compatible with the WHO Package for Essential Noncommunicable (PEN) disease interventions and provides steps of medication and patient education (https://comdis-hsd.leeds.ac.uk/resources/ncd-care-package/). The PEN/COMDIS-HSD package has been piloted in its hard copy residential course format by the FMoH/NCD division in Abuja Federal Capital Territory. However, there is no funding for scaling -up these traditional in-class service courses for health workers at a national level.

By leveraging the high mobile phone penetration in Nigeria, we aimed to pilot the use of an innovative mobile health (mHealth) user-friendly view-through rate (VTR) mobile training application that can be used on any smart phone, tablet or computer, off-line or while connected to the internet. The guide and training modules were adapted for mHealth online delivery by Instrat Global Health Solutions (an indigenous technology company) with pre/ post tests and award of continuing professional development certificates. We hypothesised that mHealth NCD training and a support group for nurses should lead to improved knowledge and improved competencies in the screening/diagnosis, treatment initiation and optimal blood pressure control in patients at PHC level. Our overarching goal was to develop a workable and sustainable approach to support health workers in managing NCDs at PHC level which can be scaled-up across Nigeria.

Methods

Implementation package

The NCD desk guide, training modules, and treatment card record package was adapted from the World Health Organization PEN guidelines by our team (http://comdis-

For peer review only - http://bmjopen.bmj.com/site/about/guidelines.xhtml

hsd.leeds.ac.uk) in collaboration with Nigeria's WHO and the Federal Ministry of Health. Doctors are at the hospital level. Registered nurses are the more qualified health workers in primary healthcare (PHC) facilities in Nigeria but have not been trained to provide NCD prevention and treatment. The NCD package had been initially piloted in a conventional face- to- face training at a venue in the Federal capital territory of Abuja with WHO Nigeria funding. However, there is no funding for scale-up across the many states and thousands of PHC facilities of Nigeria. Online mHealth training and support has potential for large scale replication.

The case management desk guideline and training module package was adapted to be feasible in the country context guidelines in collaboration with Nigeria's WHO and Federal Ministry of Health. However, the mHealth training focused on hypertension,diabetes and obesity as these are the most common

A logic model table was drawn up to guide the implementation of this pilot. (Table 1) The logic model table captures the various inputs for this project which included the case management and lifestyle counselling desk guide(lines), the mHealth training, supervisory visits, treatment cards and relevant equipment. Also reflected in the logic model table are the specific actions undertaken in the course of this project namely: screening for NCDS, prescription of relevant drugs, counselling on healthy lifestyle, focus group discussions and follow up care. These actions are then linked to intended practice changes, outputs and health outcomes (see Table 1)

Research design

In this pilot, we adopted a mixed-methods approach to:

(i) Evaluate changes in NCD related knowledge through pre-post training assessments included with the online training.

(ii) Understand service delivery and implementation contextual factors through structured observations and focus group discussions involving the health workers following training.

(iii) Assess outcomes through record review of treatment cards for appropriateness of screening, diagnosis and initiation of treatment and changes in blood pressure etc.

The implementation of this pilot was guided by the proposed theory of change that recognises the various inputs and activities expected to generate patient related outcomes and health systems impact driven by policy change. The theory is underpinned by contextual factors such as the current organisation and resourcing and limitations of the health system, limitations placed on the scope of tasks performed by health workers at PHCs, availability of funding to train health workers and the opportunity to leverage mobile phone penetration.

For peer review only - http://bmjopen.bmj.com/site/about/guidelines.xhtml

It was not possible to involve patients or the public in the design, or conduct, or reporting, or dissemination plans of our research.

Table 1: Logic model table showing intervention inputs, intervention process and intended practice change

Intervention inputs	Intervention process & actions	Intended		
		Practice change	Outputs	Health outcome
• Case management desk guideline, including lifestyle counselling<br>• mHealth training of nurses on the deskguide package<br>• Nurse WhatsApp support group<br>• Supervision of care visits (quarterly)<br>• Digital BP apparatus, glucometer & strips<br>• Treatment card, used for clinical care [subsequently also as research data records]<br>• Monthly funds for health workers' telecommunication fees	• Screen/ diagnose<br>• Prescribe anti-hypertensive<br>• Identify hypertension and/ or diabetes and treat<br>• Counsel for lifestyle modification<br>• Follow-up care<br><br>[Focus group discussion with nurses, month 5]	Provider practice:<br>• Screen, diagnose, treat, counsel, follow-up & report as per the case management desk guideline<br>Patients practice:<br>• Follow-up visits<br>• Treatment<br>• Lifestyle changes as counselled e.g. healthy eating, activity, smoking cessation	Patients are:<br>• Screened & diagnosed as per desk guide<br>• Prescribed right drug/ dose<br>• Counselled for life-style change<br>• Followed-up and treated – i.e., continuing care	• BP trend by visit [primary outcome]. Hypertension and/or diabetes care recorded on the NCD card.

Setting and participants

This pilot was carried out in a total of 19 PHC facilities in Cross River State, South-Eastern Nigeria over 12 months between February 2020 and January 2021. It involved 23 nurses

For peer review only - http://bmjopen.bmj.com/site/about/guidelines.xhtml

drawn from selected sites in rural areas and peri-urban areas in Cross River State. Purposive sampling was used to choose the 19 PHC facilities and this was done in collaboration with the Cross River State Primary Health Care Development Agency (CRSPHCDA). The CRSPHCDA is a government agency tasked with ensuring 70% of the population in CRS have access to affordable quality healthcare at primary care level. The CRSPHCDA proposed the 23 nurses from the 19 PHC facilities for the NCD training and this was based on the number of available nurses per facility. On the average, there is one nurse per PHC facility. It was spread across all the senatorial zones and local government areas with the aim of sampling from at least 10% of the just over 190 larger PHCs (staffing including at least one registered nurse) in Cross River State

Process

A one-day face-to-face orientation was organised in Calabar, the capital of Cross River State, in February 2020. This introduced the 23 health workers to the NCD training package, giving them hard copies of the desk guide, job aide, treatment cards and showed how to access and use the mHealth training modules for home self-study .

The mHealth modules were developed using the *MediXcel Lite* health technology platform which has been previously described.[23,24] The training modules consisting of video and text-based learning materials were created in English which was understood by all health workers. Several facilitation techniques were used by the trainers such as practical sessions, group discussions and case studies. All health workers were then expected to complete the eight NCD modules over a two-week period by accessing the NCD materials on their mobile phones and tablets. User logins were created for the health workers by the staff of InStrat to enable them download the App onto their android mobile phones. This App also tracked their progress with the NCD modules automatically. The maximum possible score was 100% with the pass score set at 70% for each module. Continuing professional development certificates were awarded to the health workers who completed the NCD modules.

Following this orientation and mHealth training, the health workers were expected to utilise the information contained in the modules to screen and identify patients presenting with NCDs to their various facilities. Treatment cards were distributed, and participants were instructed to upload monthly updates on the number of NCD cases seen in the various facilities via an electronic *Comm Care* App portal on the mobile electronic devices. Relevant clinical materials such sphygmomanometers, glucometers and test strips were provided for the facilities to facilitate the clinical assessment of clients with NCDs.

The health workers were provided with funds monthly to cover telecommunications fees. A community of practice (CoP) WhatsApp group was created for the health workers to facilitate peer-to-peer interactions, real-time patient care support by clinicians within the research team and data management by IT personnel of the implementation team. The WhatsApp group also served as an avenue to provide clinical support and data recording support. FDG The health workers who uploaded their facility data onto the *Comm Care App*

For peer review only - http://bmjopen.bmj.com/site/about/guidelines.xhtml

on time were openly commended as a form of non-financial motivation and as a reminder to others to do the same.

Supportive supervision was provided to the health workers in the form of weekly discussion WhatsApp phone calls and supplemented by quarterly facility clinician supervisory visits. The facility visits involved first-hand assessments of the service delivery contextual factors. During these trips, clinical mentoring was provided, and the health workers were observed providing care to patients using the clinical desk guide and treatment cards. Feedback was obtained from the health workers during these sessions.

Responding to the early implementation issues identified by the implementation team, a new module on integrating NCD management into daily activities of PHC facilities was developed and deployed on the training App.

Focus group discussions

In the 5th month from the start of implementation, focus group discussions (FGDs) were conducted as part of the research, with the health workers (see supplementary file). Short message service invitation were sent to 24 participants who completed the intervention to share their perceptions of the digital training and experiences in focus group discussions. Focus groups[25] were the chosen method as they are useful for exploring perspectives and encouraging reflections on experiences. Twenty-three participants accepted the invitation. We conducted four focus group sessions over 10 days, with an average of 6 participants in each group (FG1 n=8, FG2 n=4, FG3 n=5, FG4 n=6). There were only two male participants, and they were part of the fourth group.

Each FGD was facilitated by 2 researchers experienced in conducting FGDs and an assistant. The research assistant coordinated the session schedules, recording and served as the official note-taker. Sessions were 1 – 2 hours in length and conducted face-to-face in the place convenient for participants. Open-ended questions, formulated by the team who have formal training in qualitative research were used to gain the best perspective and scope of individual responses on (1) perceptions of the use of m-Health training strategy, (2) experiences with the implementation of clinical guidelines (3) reflections on the adequacy of received supervisions, (4) reflections on potential scale-up of the intervention. Facilitator notes ensured uniformity among FGDs and kept each discussion on target. At each FGD, an opportunity was given to withdraw consent to participate and recording. Responses were recorded, and participants were assured their comments would be kept confidential and anonymous.

No formal sample size was calculated for this pilot study.

Ethical approval for the study was received from the Cross River State Health Research Ethics Committee CRSMOH/RP/REC/2019/173.

Data analysis

For peer review only - http://bmjopen.bmj.com/site/about/guidelines.xhtml

Quantitative data

The screening, diagnosis, treatment and follow-up care is specified in the NCD guide and this information is recorded on the treatment card. The record review was of all patients identified and initiated on care. The assessment was of quality care according to these guidelines and the completeness of recording of key clinical measurements of blood pressure (BP), body mass index (BMI), fasting blood sugar (FBS) and urine analysis (as per guideline for that disease) were metrics used to assess the effectiveness of the pilot project. We assessed the effect of the NCD mHealth training through the pre/post test results and assessed operational issues that may have contributed to the results. The overall assessment was the degree to which the NCD mHealth training and care was feasible, sustainable, replicable, and treatment initiation quality was (as per guideline).

Data were imported and analysed in SPSS v23.[26] Descriptive analysis of quantitative data was carried out using means, standard deviations, frequencies, and percentages. Group differences were established using chi-square analysis. Descriptive trend analysis for the follow up rates in the systolic blood pressure and diastolic blood pressure changes in the hypertensive patients was conducted on the monthly basis. The paired t-test was used to compare the average change in systolic blood pressure and diastolic blood pressure levels at baseline and three-month follow up among the hypertensive patients. Hypothesis testing was two-tailed, at the 5% level.

Qualitative data

We used qualitative thematic analysis to identify prominent themes and patterns in the data and included a combination of deductive and emergent strategies. The facilitators debriefed immediately after each FGD. Two researchers (AO and SA) independently reviewed the data and came together to discuss emerging ideas. After the discussion, the researchers returned to the data to further review and confirm coding. This iterative process resulted in grouping the codes into three major themes: (1) perceptions with the mHealth trainings, (2) experiences with the implementation of NCD care packages, (3) potential issues with the intervention scale-up. Qualitative data were analysed using Dedoose software.[27]

The development of the research question and outcome measures in this initiative were directly informed by Nigerian patients' priorities, experience, and preferences. These priorities were factored into the design of this study. Patients were not directly involved in the recruitment to and conduct of the study but were beneficiaries of the NCD package of care we provided in the PHCs. We aim to disseminate the results of this initiative by conducting interactive sessions with the patients who continue to receive care for their NCDs in the PHCs in Cross River State.

Results

For peer review only - http://bmjopen.bmj.com/site/about/guidelines.xhtml

A total of 23 health workers from 19 primary health centres (PHCs) across the state were trained using the App. All the participating health workers achieved the pass score of 70% for each module to enable them progress to the next module. There was no limit on the number of attempts that could be made on the post module test and all the health workers were able to complete the modules in the given two weeks. The average pre-test and post-test scores were 65(±12) and 87(±8) respectively with a statistically significant difference between these scores following a two-tailed t-test ($p<0.001$).

Since the project initiation, 414 (72.7%) community members of the 569 patients seen were newly diagnosed cases of NCDs. The rest were patients who had previously been diagnosed with NCDs prior to the onset of project. . The average age of males diagnosed with an NCD was 53 years (SD= 15) and for females 49 years (SD=14). Over three-quarters (n=432, 76%) of all cases and over half (n=315, 55%) of newly diagnosed cases were hypertensive patients (newly diagnosed:110 male, 205 female, average age 52, SD=13) and 14% were diabetics (15 male, 35 female, average age 49, SD=14) (Table 1).

Fasting blood sugar checks were conducted on 215 of the community members with 47 (22%) of them falling within the prediabetes range of 5.6-6.9mmol/l and 82 (38%) falling above the diabetes threshold of >7.0mmol/l (Table 2). Proteinuria and/or glycosuria was identified in 14 of the 113 persons who had a urine analysis. In accordance with the NCD guideline, fasting blood glucose was only done if the person was overweight or had related symptoms, and urine analysis was only done as indicated.

Table 2. Characteristics of NCDs patients served by targeted PHC clinics n=569

**Characteristics**	**Male (n=206/36%)**	**Female (n=363/64%)**	**Total (n=569)**
Age (µ ± SD)	53.5±15.2	49.75±14.2	51.8 ±14.2
**NCD patients who attended facilities**			
Hypertension	155 (75.2%)	277 (76.3%)	432 (75.9%)
Diabetes	23 (11.2%)	47 (13.0%)	70 (12.3%)
Asthma/COPD	10 (4.9%)	17 (4.7%)	27 (4.7%)
Depression	1 (0.5%)	8 (2.2%)	9 (1.6%)
Sickle cell anaemia	6 (3.0%)	1(0.3%)	7 (1.2%)
Epilepsy	3 (1.5%)	3 (0.8%)	6 (1.1%)
Other NCD	8 (3.9%)	10 (2.8%)	18 (3.2%)
Total	206 (100%)	363 (100%)	569 (100%)

For peer review only - http://bmjopen.bmj.com/site/about/guidelines.xhtml

**New diagnosis of NCDs**			
Yes	145 (70.4%)	269 (74.2%)	414 (72.7%)
No	61 (29.6%)	94 (25.8%)	155 (27.3%)
Total	206 (100%)	363 (100%)	569 (100%)
**Check-up items**			
Height (µ ± SD)	1.56±0.14	1.56±0.11	1.56±0.12
Weight (µ ± SD)	67.4±12.6	68.5±12.4	68.1±12.5
**BMI**			
Normal 18.5-24	57 (27.7%)	114 (31.4%)	171 (30.0%)
Overweight 25-30	78 (37.9%)	124 (34.1%)	202 (35.5%)
Obese ≥30	62 (30.1%)	122 (33.6%)	184 (32.3%)
Total	206 (100%)	363 (100%)	569 (100%)
**Blood pressure (BP)**			
Normal BP 90/60mmHg-120/80mmHg	52 (25.2%)	73 (20.1%)	125 (22.7%)
High normal 130/85mmHg - 139/89mm Hg	21 (10.2%)	38 (10.5%)	59(10.4%)
High BP ≥140/90mmHg	133 (64.6%)	252(69.4%)	385 (67.7)
Total	206 (100%)	363 (100%)	569 (100%)
**Diagnostic tests**			
**Fasting blood sugar (FBS)**			
Normal reading <5.6mmol/l	30 (40.0%)	56 (40.0%)	86 (40.0%)
Prediabetes 5.6-6.9mmol/l	15 (20.0%)	32 (23%)	47 (21.9%)
Diabetes reading >7.0mmol/l	30 (40.0%)	52 (37.1%)	82 (38.1%)
Total	75(100%)	140 (100%)	215 (100%)
**Urine analysis**			
Normal	32(69.6%)	57(65.5%)	89 (66.9%)
Proteinuria and/or glycosuria§	14 (30.4%)	30(34.5%)	44 (33.1%)

For peer review only - http://bmjopen.bmj.com/site/about/guidelines.xhtml

Total recorded	46(100%)	87(100%)	133 (100%)

§Proteinuria- proteins in urine, glycosuria-sugar in urine. According to the NCD guideline, fasting blood glucose taken if overweight or have related symptoms, and urine analysis as indicated.

Gender distribution of newly diagnosed NCD cases (Figure 1) was not statistically different $\chi 2$ (6) =8.4, p=0.2. On average, 16 hypertensive and 3 diabetic patients were identified per facility (range 3-38 for hypertensive and 0-12 for diabetic patients).

Figure 1. Gender distribution of newly diagnosed NCD cases

All NCD patients (n=569) registered at targeted facilities received height (μ=1.56, SD=0.12) and weight measurements (μ=68.1, SD=12.47) at the first appointment. BMI was calculated and recorded for all of these patients (μ=28.1, SD=5.85). Over forty percent of NCD patients (n=234, 41%) received an appointment reminder following the screening appointment.

Healthy lifestyle advice

In all, 356 (62.6 %) patients were given lifestyle advice such as reducing/ stopping cigarette smoking and alcohol, reducing weight, reducing salt intake, increasing physical activity/ exercise, reducing stress, medication adherence, healthy eating, and healthy living. For these a motivational counselling strategy was used.

Primary outcome

Following the initial screening visit involving 420 patients, the patients were followed up with 230 of them making two of such follow-up visits. A total of 98 patients made up to three follow-up visits (Table 3). Recordings on treatment cards indicated a positive significant improvement in blood pressure of hypertensive patients from visit 1 through to visit 3 (Figure 2). The systolic blood pressure for hypertensive patients who had three follow-up visits (after the intial screening visit) steadily decreased from the visit 1 average of 162 to a visit 3 average of 139 mmHg. The average systolic blood pressure decreased 23 mmHg between the screening to follow-up visit 3 (p<0.001). A similar trend was registered in decrease of 12mmHg in diastolic blood pressure from follow-up visit 1 average of 93.8mmHg to follow-up visit 3 average of 81.7mmHg (p<0.001).

Figure 2: Trends in blood pressure change among hypertensive patients (n=428)

Table 3: Trend on blood pressure from visit 1 to visit 3 based on average of two measurements

Parameter	Value	All patients (screening	patients with 2 visits	patients with 3 visits

For peer review only - http://bmjopen.bmj.com/site/about/guidelines.xhtml

For peer review only

		visit)	Visit 1	Visit 2	Visit 1	Visit 3
Systolic BP (mmHg)	Valid values	420	230	230	98	98
	Mean (±SD)	159.9 (23.3)	160.3 (20.7)	143.2 (20.6)	162.1 (21.4)	139.3 (19.1)
	Range	90-270	90- 220	99-218	90- 220	80-180
	Values >140 (%)	77.6%	79.6	43%	83.7	32.3%
	Values >180 (%)	15.0%	14.8	3.9%	16.3	--
Diastolic BP (mmHg)	Valid values	420	230	230	98	98
	Mean (±SD)	96.0 (14.3)	95.3(13.5)	85.5 (12.3)	93.8 (15.3)	81.7 (11.1)
	Range	50-174	50- 152	80-130	50- 152	50-120
	Values >90 (%)	60.7%	59.1	22.6%	53.1	13.3%
	Values >110 (%)	10.2%	7.8	3.9%	9.2	1.0%

Compliance with medication

The hypertensive patients were prescribed antihypertensive drugs from the following classes: (1) Angiotensin-converting enzyme inhibitors (lisinopril, captopril), (2) Calcium channel blockers (nifedipine, amlodipine), (3) Diuretics (furosemide, hydrochlorothiazide) which were all in line with recommendations provided in the NCD deskguide. Where hydrochlorothiazide was unavailable, normoretic (i.e. amiloride hydrochloride 5mg and hydrochlorothiazide 50mg) was prescribed. The top three most often prescribed antihypertensive drugs were normoretic (50%), nifedipine (44%) and amlodipine (33%). Diabetic patients were mostly prescribed metformin, glibenclamide, or insulin injections.

Focus group discussions

Participants

Focus groups included 21 women and 2 men, between 31 to 60 years of age. All participants had post-secondary training in nursing. Some of the participants had prior experience of attending digital training. However, NCDs had not been prioritised in the health worker basic or post basic training the participant had previously received.

For peer review only - http://bmjopen.bmj.com/site/about/guidelines.xhtml

Perceptions of m-health training

Content

The mHealth training was stated to be a valuable experience. Participants were very appreciative of the knowledge they acquired about prevention and control of NCDs. They talked about improved confidence and an increased sense of empowerment in their ability to provide counselling on lifestyle behaviours and medication adherence. This positively influenced their internal sense of importance, consistent with improved self-efficacy.

> *"Some of these cases … before now I didn't know how to handle them but now I am very confident in handling the issues. The way and manner I am able to regulate clients' blood pressure are making me feel important" [FG1].*

> *"I will begin by appreciating the NCD project and people. Before now, we actually didn't know how to handle these cases but now we are very confident. We know the particular drugs to prescribe and the dosage". [FG2]*

Functionality

Participants perceive the functionality of m-health technology and ease of use as important. Multiple benefits of online learning were noted including the ability to learn at your own pace, the opportunity to return to the material when needed, using smart phones to access on the VTR the NCD deskguide and modules.

> *"We are upgraded. It makes the provider more educated, because you read at your pace, you don't stress yourself, anytime you come back you read and understand. So.. I think it was great."[FG1].*

> *"It was a nice one. Almost all the trainings we have attended have not been this type. It has made us to know how to use our phone in learning online and also searching for other things you would not have ordinarily done because of the workload where we find ourselves. It is a good one. Many times, we tell ourselves let's go online something is happening "[FG4].*

Experiences with the implementation of NCD care package

We interviewed 29 patients randomly selected across all the facilities. Of these, 21(72.4%) were satisfied with the main reason for dissatisfaction being our initiative's inability to provide medications.

Positive impact

Participants talked highly about positive changes the integrated care delivery brought to the communities they served. Provision of BP and blood glucose diagnostic equipment meant targeted diagnostic and curative services delivery at a point of access.

For peer review only - http://bmjopen.bmj.com/site/about/guidelines.xhtml

"The NCDP [the project] has made us have the necessary equipment to carry out blood pressure, diabetes diagnosis in my facility. This is a great achievement really. People come in now to say I want to check my blood pressure and sugar." [FG4]

When people come to our ANC, we have some of the test kits to use and we started testing. We admitted some people. When people come to visit them, they go back to the community to tell others. Presently, we have increased client flow. It is helping us to detect these NCDs". [FG4]

The provision of PHC facility services dramatically improved relationships with communities and encouraged follow-up visits. During outreaches, health workers were able to use the donated sphygmomanometers to measure blood pressure within the communities and refer people to the PHC facility.

"The relationship with the community as a result of the NCD is marvellous wherever we meet with these clients they greet one with respect and are open to me" [FG1]

"Giving the test that was not existing in the community before really help to mobilize the people. They feel more relaxed and confident to come to the facility".[FG4]

Participants described a variety of community education strategies to raise awareness of the availability of NCD services. Some strategies involved monetary reward for spreading the word about services by the town crier, and also use of local worship places and/or community organisation to engage with communities.

"…I have a relationship with the town announcer. Although when people come and take the test, we collect a token from them, and I use that to pay the town announcer. I type what I want him to announce. He reads through it and announces it to the people. From there people started coming". [FG4]

"This project has impacted on our knowledge, and we can confidently do health talk and create awareness about blood pressure, and other NCDs efficiently. My wish is.., I hope this project stays, honestly." [FG1]

Challenges

Participants, however, also experienced, or perceived challenges to the implementation of care packages as extending across clinical, organisational and societal levels.

Challenges at the clinical level

For peer review only - http://bmjopen.bmj.com/site/about/guidelines.xhtml

Factors identified as challenges at the clinical level included increased workload associated with (a) ensuring the continuity of NCD care delivery, and (2) filling in the treatment cards. Participants noted the NCD initiatives' success meant the increased flow of patients to facilities for check-ups and follow-up consultations. Although this has generally been perceived as a positive dynamic, the increase in workload was mentioned.

> *"NCDs increased our work. Before now, we see them and they go away, we don't bother to follow them up or they don't come back to us to maintain their BP. We need to maintain them to get their drugs or make sure their blood sugar level got controlled. Some of us, our facilities are easy to get to, so they can easily come. Even if you are resting, they want you to come and check them. When they heard that someone in the community died due to high blood pressure, that period a lot of people came for checking [FG4].*

Some participants also noted that filling the treatment cards and registers was time-consuming and often discouraged their use. Content reduction was a proposed solution to address this.

> *"Ordinarily we have few questions to capture from clients but the NCD has widened a lot of information that needs to be captured, looking at this with other registers in the facility too has made the workload high, and again there are series of tests that also needs to be run on an individual basis." [FG1].*

Technical glitches in the application and internet connection were also noted resulting in issues with uploading data into the central system. This resulted in double entry of data which was perceived as a double burden on staff. Some participants also recognised that supervision and monitoring provided by staff of the initiative helped them navigate many challenges along the way.

Challenges at the organisational level

The presence of professional support or opposition was highlighted as a considerable factor for the successful integration of the care packages into daily routine. Participants talked in length about conflict of interest between the focal person responsible for NCD care and PHC facility in-charge. Prior to the training, there was no focal person responsible for NCD in these facilities. Following the NCD training, the 23 nurses were encouraged to assume the role of NCD focal person in their respective facilities. The PHC facility in-charges were typically registered nurses who also doubled as the administrative head of the health facilities. Examples of interference from *person-in-charge* into modes of care delivery, equipment storage and use as well as generated income distribution were noted.

For peer review only - http://bmjopen.bmj.com/site/about/guidelines.xhtml

"The thing is really disturbing because the in-charge kept telling you that the equipment is not for you and should be kept in the laboratory, giving unnecessary orders before you can make use of it. It pissed me off a lot." [FG1].

Some participants, on the other hand, talked positively about PHC facility in-charge and the support they received. They also acknowledged how some managers actively "champion" the awareness-raising activities amongst communities, and how this considerably increased the flow of patients to facilities.

"The new in charge has leadership skills. He went to the community heads and introduced himself, he went to churches. He will encourage sick people to always come and test. A lady died and it was discovered her blood pressure was high. So, they announced in all the churches that people should go and check their blood pressure and sugar level. This really helped us. [FG4]

Although many participants talked in terms of conflict, in general, negotiation, avoiding confrontations and/ or for managers undergoing training on NCD care alongside junior staff were preferred approaches to resolving the problems.

"I believe they should train them. Since we have this knowledge, they know some of the drugs, but they don't know the right time to administer. If they are exposed to this training and they are told the stages and when to prescribe, they will know what to do. For example, in my facility my in-charge prescribed stage II drugs instead of stage I, I had to correct him. If they know, they will do better." [FG4]

Participants also noted negative changes in the spirit of collegiality and the need to ensure that *"the focal persons"* have the expertise to manage NCD cases. This point reflected the need for such training to be stepped down to other staff who may not have attended the initial face-to-face orientation meeting.

Challenges at societal level

The restrictions to social interaction that followed the emergence of COVID-19 substantially limited the number of individuals attending the pilot PHCs for care. This had a negative impact on the number of persons who were eventually screened for NCDs. Also, some civil unrest occurred during the course of the project as part of the nationwide protests which resulted in vandalisation of some of the pilot PHCs. This halted services in the affected PHCs for several weeks and scaled back their recruitment rates after they reopened.

Potential issues with the intervention scale-up

For peer review only - http://bmjopen.bmj.com/site/about/guidelines.xhtml

Participants talked about pressing issues in a healthcare system that potentially can have a negative impact on the scale-up of the intervention. The most often mentioned challenges identified were health personnel shortage (especially nurses), lack of knowledge on NCD management and control, lack of essential diagnostic equipment and medication necessary for effective NCD care delivery.

Potential key solutions for ensuring the sustainability and potential scale-up of care packages at a national level were (a) targeted training of other staff on NCD prevention and management, and (b) task-shifting activities for NCD care. Participants also noted that staff at primary care level lacked basic knowledge on NCD care and if left at their own disposal many essential diagnostic tests will be missed. Therefore, they will be willing to provide training to junior staff to ensure effective delivery of services.

> *"Yes…, because some things, if left for the facility to carry on, will not be effective, but with a focal person she will ensure things are in order, since they know they will be held responsible" [FG1].*

> *"Yes, like in my facility I am the only staff I will appreciate if another staff is sent here so that we can have more hands, and I will give the person on the job training or some kind of step-down training to enable whoever is coming to render the services. It will be bad if this NCDP [the NCD project] stops, before now we have had cases where somebody will just fall while walking. The next thing you hear is people will be saying he is a ghost, not knowing is the effect of high blood pressure. So,…. I really don't want this NCD thing to just fade like that, I don't know how to put it really" [FGD2].*

Discussion

Main findings

In this mixed-methods pragmatic implementation of health worker training using an mHealth platform complemented by clinical supervision visits and a community of practice forum, we have demonstrated the feasibility of delivering training remotely leading to improvement in knowledge and skills in NCD screening, diagnosis, and treatment. In addition, we have documented nurses' experiences, perspectives, and challenges as well as the feasibility of task-shifting to nurses. This cohort of patients was found to have improved trends on average control of blood pressure. There was a statistically significant reduction on both systolic and diastolic blood pressures between the first and third visits among hypertensive patients. Nonetheless, the results do not prove a causal relationship between the mhealth training intervention and the resultant trend in blood pressure. Diuretics and calcium channel blockers (nifedipine and amlodipine) were the most commonly prescribed antihypertensives with metformin and glibenclamide being the most commonly prescribed

For peer review only - http://bmjopen.bmj.com/site/about/guidelines.xhtml

For peer review only

therapies for diabetes mellitus. These prescribing practices were all in line with the recommendations within the NCD guide.

Health education/counselling was given to about two-thirds of patients on all visits. The process indicators demonstrate the ability of nurses in PHC clinics to successfully deliver the key components of NCD care. The NCD guide was designed for qualified staff at the level of a 3-year trained registered nurse. The deployment of an mHealth platform rather than a face-to-face classroom style model to deliver the training has been shown to be feasible and effective. This is seen in the improved knowledge scores upon completion of the modules and assessment of skills seen in ability to initiate care for patients with NCDs.

The mHealth NCD training was perceived as a valuable experience with the health workers identifying multiple benefits of online learning and the integrated NCD care delivery package. This is important as there is not funding sufficient for face-to-face training (including per diems and venue cost) for nation-wide scale up of NCD training and supervision across Nigeria, as in other low-middle income countries - there is no 'Global Fund for NCDs, as there is for AIDS, TB, Malaria. We provided a one-day face-to-face orientation session to introduce the health workers to the mHealth platform and later supported them remotely to complete the modules online. This hybrid form of training appears to be suitable for the Nigerian context where face-to-face training of large numbers of health workers might not be feasible.

Challenges to the smooth rollout of this NCD package were the increased workload it generated, the technical problems with internet connection and difficulty in securing the support of health centre in-charges for the project. Remote and periodic onsite supervision by local doctors and the provision of the materials (i.e BP equipment, glucometres etc) that were lacking at the facilities were additional strategy adopted to support this initiative. The availability of these materials along with the face-to-face orientation, provision of monthly funds for health workers' telecommunication fees and hard copies of the training materials contributed to the overall success of the pilot. Therefore, such extra provisions will need to be taken into consideration when implementing a scale up this NCD package. Considerations such as appropriate diagnosis, quality of care and linkage to continuing care will need to be further addressed using a health systems framework that deals with these challenges more comprehensively.[28]

While the pilot appears to be successful, the sustainability and replicability of this model of training and care initiation will depend on addressing several health system barriers such as appropriate staffing (not all facilities have a nurse), provision of medicines and diagnostic equipment, improving on the governance system at facility level and formally task shifting to nurses the NCD care and prescribing. In addition, the reference to the initiative as a 'project' by nurses implies a risk to sustainability, if the NCD initiative is not wholly adopted by the state health management. Despite its implementation through the routine system by a local medical NGO, the perception of it as a 'project' may undermine the long-term

For peer review only

For peer review only - http://bmjopen.bmj.com/site/about/guidelines.xhtml

sustainability at facility level. This mixed methods research adds to the evidence base of similar NCD interventions in resource-limited settings. Using a similar shared care guide for management of diabetes and hypertension care in Eswatini (southern Africa), nurses were able to successfully deliver diabetes and hypertension care with improvements in physiological parameters, though continued training and supervision were required to consolidate change.[14] A similar NCD guideline and training COMDIS-HSD partner initiative in Pakistan was found to be effective in trials and was scaled across the Punjab Province of 110 million population.[16-20] This Nigeria pilot adds evidence for the potential in a West African setting and for mHealth as an alternative to expensive residential courses, which is a major constraint to NCD care scale-up.

*Limitations*The emergence of COVID-19 and imposed public health restrictions substantially limited the number of individuals attending the pilot PHCs to be screened and diagnosed over the equally short period of ten months. Community mobilization was conducted by the trained nurses to help overcome some social barriers to accessing care that came in the wake of the COVID-19 pandemic. Some of the health facilities were vandalised during the nationwide protests that engulfed Nigeria in October 2020, and this also had a negative impact on the NCD care services. In addition, the pragmatic nature of our initiative precluded the provision of medicines, which may have led to an increase in NCD patients observed. Furthermore, given that many NGOs are operating in fragile health environments, pilot initiatives may not be subsequently implemented by ministries of health.

The observational nature of our study precludes from drawing decisive conclusions as to whether care under the new NCD care model is better or worse than the alternative conventional residential training practiced.

Conclusion

This pilot initiative provides evidence of feasibility of implementing an NCD care package supported by mHealth training. The complimentarysupport provided for health workers in the form of supervisory visits, a community of practice forum and ancillary equipment facilitated their ability to screen for NCDs in the PHCs. This utility of this approach is reflected in the improvement in BP measurements in clients in follow-up visits to the PHCs. These results support the implementation of such a package of care at scale within the Nigerian context and similar settings.

Acknowledgements: We appreciate the NCD division of the FMOH, Nigeria, Cross River State Ministry of Health and Primary Healthcare Development Agency as well as nurses and facility heads who participated in the pilot. We are grateful to all the patients who accepted to be managed using the NCD guides in the various health facilities in Cross River State Nigeria. We are thankful to Mr Tijesu Ojumu of the Nigeria Centre for Disease Control for

For peer review only - http://bmjopen.bmj.com/site/about/guidelines.xhtml

designing the conceptual framework. Our sincere gratitude goes to the NIHR RUHF project of QMU Edinburgh for funding this initiative.

Contributorship statement: AO, EE, ObO and JW conceived the study. AO, EE, ObO, OO and CO were involved in the implementation of the programme. EE, ObO, OO and CO handled the data entry. SA, AO, ObO, JW and OO did the data cleaning and analysis. AO, SA, EE, JW and ObO wrote the first draft. All authors were involved in the reviewing and editing of this article and gave final approval of the version to be published. AO is the guarantor of the article.

Competing interests: None declared.

Funding: This study/project is funded by the UK National Institute for Health Research (NIHR) [NIHR Global Health Research programme (project reference 16/136/100)/ NIHR Research Unit on Health in Situations of Fragility]. The views expressed are those of the author(s) and not necessarily those of the NIHR or the Department of Health and Social Care. The funder played no role in the study design, data collection, analysis, interpretation, or in writing the manuscript.

Data sharing statement: Data generated by your research that supports your article will be made openly and publicly available upon publication of your article.

Patient consent for publication: not required

Ethics approval: This study was performed in line with the principles of the Declaration of Helsinki. The study received the approval of the Health Research and Ethics Committee of the Cross River State Ministry of Health Nigeria (Number/ID: CRSMOH/RP/REC/2019/173). Participation was voluntary.

Data availability statement: No data are available.

Supplemental material: None supplied.

Patient and Public Involvement: The development of the research question and outcome measures in this initiative were directly informed by Nigerian patients' priorities, experience, and preferences. These priorities were factored into the design of this study. Patients were not directly involved in the recruitment to and conduct of the study but were beneficiaries of the NCD package of care we provided in the PHCs. We aim to disseminate the results of this initiative by conducting interactive sessions with the patients who continue to receive care for their NCDs in the PHCs in Cross River State.

References

For peer review only

1. World Health Organization 2021. Noncommunicable diseases – key facts. Available: https://www.who.int/news-room/fact-sheets/detail/noncommunicable-diseases
2. World Health Organization - Noncommunicable Diseases (NCD) Country Profiles. Available: https://www.who.int/nmh/countries/nga_en.pdf?ua=1
3. World Health Organization [WHO]. Global health observatory data 2018: Premature NCD deaths. Available: https://www.who.int/gho/ncd/mortality_morbidity/ncd_premature_text/en/
4. Enang OE, Otu AA, Essien OE, *et al*. Prevalence of dysglycemia in Calabar: a cross-sectional observational study among residents of Calabar, Nigeria. *BMJ Open Diabetes Res Care* 2014; 2(1):e000032. doi: 10.1136/bmjdrc-2014-000032
5. Effa E, Onwusaka, O, Ekrikpo U, *et al*. Community based screening for risk factors of chronic kidney disease in Cross River State. *Kidney International Reports* 2020; 5(3):S58-S59.
6. Maiyaki M, Garbati M. The burden of non-communicable diseases in Nigeria; In the context of globalization. *Ann Afr Med* 2014;13:1–10.
7. World Health Organization. Noncommunicable diseases: the slow motion disaster. Geneva: WHO; 2017.
8. Crush J, Frayne B, McLachlan M. "Rapid Urbanization and the Nutrition Transition in Southern African." Urban Food Security Series No. 7. Queen's University and AFSUN: Kingston and Cape Town. 2011.
9. Maher D, Sekajugo J. Research on health transition in Africa: time for action. *Health Res Policy Syst* 2011; 9:1–4.
10. Green A. Country in focus: the growing burden of NCDs in Nigeria. *The Lancet Diabetes and Endocrinology* 2016; 4(1): 23-24
11. Ajayi T. What is Mental Health to an African? Available: https://www.linkedin.com/pulse/what-mental-health-african-tomiwa-ajayi
12. National multi-sectoral action plan for the prevention and control of non-communicable diseases (2019 – 2025). Available: https://www.iccp-portal.org/system/files/plans/NCDs_Multisectoral_Action_Plan.pdf
13. Federal Ministry of Health Nigeria 2014. Task-shifting and task-sharing policy for essential health care services in Nigeria. Available: file:///C:/Users/akaninyene.otu/Downloads/TSTS.pdf
14. Sharp A, Riches N, Mims A, *et al*. Decentralising NCD management in rural southern Africa: evaluation of a pilot implementation study. *BMC Public Health* 2020*;* 20:44
15. Walley J, Khan MA, Witter S, et al. Embedded health service development and research: why and how to do it (a ten-stage guide). *H Research Policy* & *Systems*. 2018; D-17-*00256R1*
16. Khan MA, Khan N, Walley JD, *et al*. Enhanced hypertension care through private clinics in Pakistan: a cluster randomised trial. *BJGP Open*. 2019; 3(1):bjgpopen18X101617.

For peer review only - http://bmjopen.bmj.com/site/about/guidelines.xhtml

17. Khan MA, Walley JD, Khan N, *et al*. Effectiveness of an integrated diabetes care package at primary healthcare facilities: a cluster randomised trial in Pakistan. *BJGP Open.* 2018;2(4):bjgpopen18X101618. doi:10.3399/bjgpopen18X101618
18. Khan N, Khan M, Walley J, et al. A process evaluation of integrated diabetes management at primary health care facilities in Pakistan. *BJGP Open*. 2018;2(4):bjgpopen18X101612
19. Khan MA, Khan N, Walley J, *et al*. How effective is it to deliver integrated COPD care at public healthcare facilities: a cluster randomised trial in Pakistan? BJGP Open 2019; 3 (1). bjgpopen18X101634. ISSN 2398-3795
20. Khan MA, Khan MA, Walley J, *et al*. Feasibility to deliver integrated COPD-Asthma care at primary and secondary level public healthcare facilities in Pakistan: a process evaluation. BJGP Open 2019; DOI: 10.3399/bjgpopen18X101632
21. Wei X, Walley JD, Zhang Z, et al. Implementation of a comprehensive intervention for patients at high risk of cardiovascular disease in rural China: A pragmatic cluster randomized controlled trial. *PloS One* 2017; 12 (8). e0183169.
22. Witter S, Zou G, Diaconu, K. et al. Opportunities and challenges for delivering non-communicable disease management and services in fragile and post-conflict settings: perceptions of policy-makers and health providers in Sierra Leone. *Confl Health* 2020; 14, 3. https://doi.org/10.1186/s13031-019-0248-3
23. Otu, A, Okuzu, O, Ebenso, B, et al. Introduction of mobile health tools to support COVID-19 training and surveillance in Ogun State Nigeria. *Front Sustain Cities* 2021; 3: 10.
24. Otu A, Okuzu O, Effa E, et al. Training health workers at scale in Nigeria to fight COVID-19 using the InStrat COVID-19 tutorial app: an e-health interventional study. *Therapeutic Advances in Infectious Disease* 2021. doi:10.1177/20499361211040704
25. Willis K, Green J, Daly J, *et al*. Perils and possibilities: achieving best evidence from focus groups in public health research. *Aust N Z J Public Health.* 2009;33(2):131–6
26. IBM Corp. Released 2015. IBM SPSS Statistics for Windows, Version 23.0. Armonk, NY: IBM Corp.
27. Dedoose Version 9.0.17, web application for managing, analyzing, and presenting qualitative and mixed method research data (2021). Los Angeles, CA: SocioCultural Research Consultants, LLC www.dedoose.com.
28. Bloomfield GS, Vedanthan R, Vasudevan L et al. Mobile health for non-communicable diseases in Sub-Saharan Africa: a systematic review of the literature and strategic framework for research. Global Health. 2014 Jun 13;10:49. doi: 10.1186/1744-8603-10-49.

For peer review only - http://bmjopen.bmj.com/site/about/guidelines.xhtml

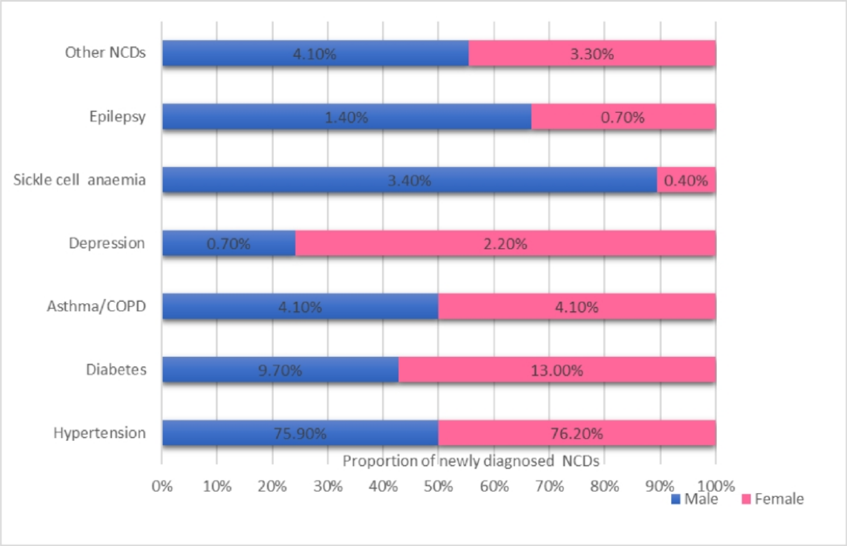

Figure 1. Gender distribution of newly diagnosed NCD cases

338x190mm (200 x 200 DPI)

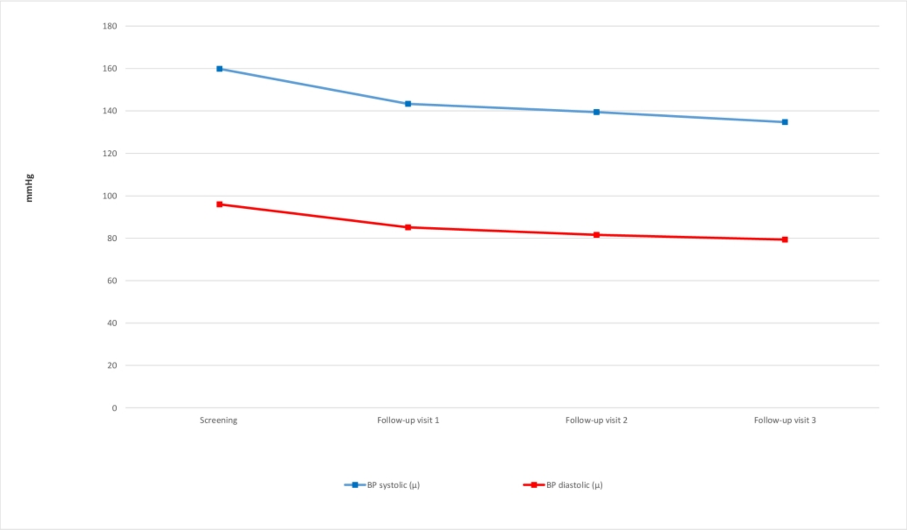

Figure 2. Trends in blood pressure change among hypertensive patients (n=428)

338x190mm (200 x 200 DPI)

Tackling non-communicable diseases in primary care facilities Nigeria through clinical guidelines and m-Health training strategies.

FOCUS GROUP DISCUSSION GUIDE

1. Perception of the use of the m-Health strategy- mode of delivery & content of training resources, challenges they have encountered as well as recommendations on the way forward.
- Satisfaction (or not) with the skills and knowledge from the training modules
- Were the modules sufficient enough to prepare you to carry out these services (NCD management)

2. Implementation issues;
   - Integrating NCD management into routine activities
   - Role of additional resources in program strengthening- clinical guidelines/ desk guides,
   - treatment cards, basic diagnostic equipment, monthly support for telecommunications

? – how did the project impact/ change your
-workload
-Income
-working relationship with colleagues
- relationship with community

3. Adequacy of Supervision:
   - Clinical supervisory visits (onsite and remote)
   - Monitoring
4. Moving forward, do you think your facility can continue providing these services even after the project ends?
5. Lessons learned

For peer review only

Standards for Reporting Qualitative Research (SRQR)*

http://www.equator-network.org/reporting-guidelines/srqr/

Title and abstract

	Page/line no(s).
**Title** - Concise description of the nature and topic of the study Identifying the study as qualitative or indicating the approach (e.g., ethnography, grounded theory) or data collection methods (e.g., interview, focus group) is recommended	Page 1, line 2-3
**Abstract** - Summary of key elements of the study using the abstract format of the intended publication; typically includes background, purpose, methods, results, and conclusions	Page 2, line 33-66

Introduction

**Problem formulation** - Description and significance of the problem/phenomenon studied; review of relevant theory and empirical work; problem statement	Page 4, line 113-133
**Purpose or research question** - Purpose of the study and specific objectives or questions	Page 4, line 213-130

Methods

**Qualitative approach and research paradigm** - Qualitative approach (e.g., ethnography, grounded theory, case study, phenomenology, narrative research) and guiding theory if appropriate; identifying the research paradigm (e.g., postpositivist, constructivist/ interpretivist) is also recommended; rationale**	Page 6 and 7, Line197-221
**Researcher characteristics and reflexivity** - Researchers' characteristics that may influence the research, including personal attributes, qualifications/experience, relationship with participants, assumptions, and/or presuppositions; potential or actual interaction between researchers' characteristics and the research questions, approach, methods, results, and/or transferability	Page 6, line 205 -211
**Context** - Setting/site and salient contextual factors; rationale**	Page 4, line 133-145
**Sampling strategy** - How and why research participants, documents, or events were selected; criteria for deciding when no further sampling was necessary (e.g., sampling saturation); rationale**	Page 7, line 245 - 252
**Ethical issues pertaining to human subjects** - Documentation of approval by an appropriate ethics review board and participant consent, or explanation for lack thereof; other confidentiality and data security issues	Page 20, line 656-659
**Data collection methods** - Types of data collected; details of data collection procedures including (as appropriate) start and stop dates of data collection and analysis, iterative process, triangulation of sources/methods, and modification of procedures in response to evolving study findings; rationale**	Page 7, line 245-252

For peer review only

For peer review only - http://bmjopen.bmj.com/site/about/guidelines.xhtml

**Data collection instruments and technologies** - Description of instruments (e.g., interview guides, questionnaires) and devices (e.g., audio recorders) used for data collection; if/how the instrument(s) changed over the course of the study	Page 7, line 245-252
**Units of study** - Number and relevant characteristics of participants, documents, or events included in the study; level of participation (could be reported in results)	Page 12, line 322-325
**Data processing** - Methods for processing data prior to and during analysis, including transcription, data entry, data management and security, verification of data integrity, data coding, and anonymization/de-identification of excerpts	Page 12, line 322-325
**Data analysis** - Process by which inferences, themes, etc., were identified and developed, including the researchers involved in data analysis; usually references a specific paradigm or approach; rationale**	Page 12, line 322-325
**Techniques to enhance trustworthiness** - Techniques to enhance trustworthiness and credibility of data analysis (e.g., member checking, audit trail, triangulation); rationale**	Page 12, line 322-325

Results/findings

**Synthesis and interpretation** - Main findings (e.g., interpretations, inferences, and themes); might include development of a theory or model, or integration with prior research or theory	Page 12-16
**Links to empirical data** - Evidence (e.g., quotes, field notes, text excerpts, photographs) to substantiate analytic findings	Page 17, line 540-579

Discussion

**Integration with prior work, implications, transferability, and contribution(s) to the field -** Short summary of main findings; explanation of how findings and conclusions connect to, support, elaborate on, or challenge conclusions of earlier scholarship; discussion of scope of application/generalizability; identification of unique contribution(s) to scholarship in a discipline or field	Page 3, line 62-72
**Limitations** - Trustworthiness and limitations of findings	Page 19, line 551-559

Other

**Conflicts of interest** - Potential sources of influence or perceived influence on study conduct and conclusions; how these were managed	Page 20, line 654
**Funding** - Sources of funding and other support; role of funders in data collection, interpretation, and reporting	Page 20, line 647-653

*The authors created the SRQR by searching the literature to identify guidelines, reporting standards, and critical appraisal criteria for qualitative research; reviewing the reference lists of retrieved sources; and contacting experts to gain feedback. The SRQR aims to improve the transparency of all aspects of qualitative research by providing clear standards for reporting qualitative research.

For peer review only - http://bmjopen.bmj.com/site/about/guidelines.xhtml

**The rationale should briefly discuss the justification for choosing that theory, approach, method, or technique rather than other options available, the assumptions and limitations implicit in those choices, and how those choices influence study conclusions and transferability. As appropriate, the rationale for several items might be discussed together.

Reference:

O'Brien BC, Harris IB, Beckman TJ, Reed DA, Cook DA. **Standards for reporting qualitative research: a synthesis of recommendations.** *Academic Medicine*, Vol. 89, No. 9 / Sept 2014 DOI: 10.1097/ACM.0000000000000388

For peer review only

For peer review only - http://bmjopen.bmj.com/site/about/guidelines.xhtml

BMJ Open

mHealth guideline training for non-communicable diseases in primary care facilities in Nigeria: a mixed methods pilot study

Journal:	*BMJ Open*
Manuscript ID	bmjopen-2021-060304.R3
Article Type:	Original research
Date Submitted by the Author:	07-Jul-2022
Complete List of Authors:	Otu, Akaninyene; University of Calabar, Internal Medicine<br>Effa, Emmanuel; University of Calabar, Renal Unit, Department of Internal Medicine; University of Cape Town, Division of Nephrology and Hypertension<br>Onwusaka, Obiageli; University of Calabar<br>Omoyele, Chiamaka; Federal Ministry of Health<br>Arakelyan, Stella; Queen Margaret University Institute for International Health and Development<br>Okuzu, Okey; Instrat Global Health Solutions<br>Walley, John; University of Leeds, Nuffield Centre for International Health and Development, Leeds Institute of Health Sciences
**Primary Subject Heading**:	Public health
Secondary Subject Heading:	Global health, Public health
Keywords:	PUBLIC HEALTH, Health informatics < BIOTECHNOLOGY & BIOINFORMATICS, EDUCATION & TRAINING (see Medical Education & Training)

SCHOLARONE™
Manuscripts

I, the Submitting Author has the right to grant and does grant on behalf of all authors of the Work (as defined in the below author licence), an exclusive licence and/or a non-exclusive licence for contributions from authors who are: i) UK Crown employees; ii) where BMJ has agreed a CC-BY licence shall apply, and/or iii) in accordance with the terms applicable for US Federal Government officers or employees acting as part of their official duties; on a worldwide, perpetual, irrevocable, royalty-free basis to BMJ Publishing Group Ltd ("BMJ") its licensees and where the relevant Journal is co-owned by BMJ to the co-owners of the Journal, to publish the Work in this journal and any other BMJ products and to exploit all rights, as set out in our licence.

The Submitting Author accepts and understands that any supply made under these terms is made by BMJ to the Submitting Author unless you are acting as an employee on behalf of your employer or a postgraduate student of an affiliated institution which is paying any applicable article publishing charge ("APC") for Open Access articles. Where the Submitting Author wishes to make the Work available on an Open Access basis (and intends to pay the relevant APC), the terms of reuse of such Open Access shall be governed by a Creative Commons licence – details of these licences and which Creative Commons licence will apply to this Work are set out in our licence referred to above.

Other than as permitted in any relevant BMJ Author's Self Archiving Policies, I confirm this Work has not been accepted for publication elsewhere, is not being considered for publication elsewhere and does not duplicate material already published. I confirm all authors consent to publication of this Work and authorise the granting of this licence.

For peer review only

Title:

mHealth guideline training for non-communicable diseases in primary care facilities in Nigeria: a mixed-methods pilot study

Akaninyene Otu[1,2], Emmanuel Effa[1,2], Obiageli Onwusaka[2,3], Chiamaka Omoyele[4], Stella Arakelyan[5], Okey Okuzu[6], John Walley[7]

1. Department of Internal Medicine, College of Medical Sciences, University of Calabar, Calabar, Cross River State, Nigeria
2. Foundation for Healthcare Innovation and Development, Calabar, Cross River State, Nigeria.
3. Department of Public Health Faculty of Allied Medical Sciences, College of Medical Sciences, University of Calabar, Calabar, Nigeria.
4. Monitoring & Evaluation Branch, Non-Communicable Diseases Division, Federal Ministry of Health, Abuja, Nigeria
5. NIHR Research Unit on Health in Fragility, Institute for Global Health and Development, Queen Margaret University, Edinburgh, Scotland
6. InStrat LLC, Montclair, NJ USA
7. Nuffield Centre for International Health and Development, Leeds Institute of Health Sciences, University of Leeds, Leeds, UK

Corresponding Author:

Dr Akaninyene Otu

Department of Internal Medicine, College of Medical Sciences

University of Calabar, Calabar, Cross River State, Nigeria

E mail: akanotu@yahoo.com

For peer review only - http://bmjopen.bmj.com/site/about/guidelines.xhtml

Abstract

Objective: To pilot the use of a scalable innovative mobile health (mHealth) non-communicable diseases (NCDs) training application for nurses at the primary care level.

Design: Mixed methods pilot of mHealth training on NCD care for nurses at primary health care (PHC) facilities. We provide a descriptive analysis of mHealth training test scores, with trend analysis of blood pressure control using paired t-test for quantitative data and thematic analysis for qualitative data.

Setting: Primary Healthcare facilities in rural and urban communities in Cross River State, south eastern Nigeria. NCDs were not part of routine training previously. As in most low-and middle-income settings, funding for scale-up using conventional classroom in-service training for NCDs is not available in Nigeria, and onsite supervision poses challenges.

Participants: Twenty-three nurses in 19 PHC facilities.

Intervention: A self-paced mHealth training module on an NCD desk guide was adapted to be applicable within the Nigerian context in collaboration with the Federal Ministry of Health. The training focused on hypertension, diabetes and sickle cell disease was delivered via android tablet devices, supplemented by quarterly onsite supervision and group support via WhatsApp. The training was evaluated with pre/post-course tests structured observations and focus group discussions. This was an implementation pilot assessing the feasibility and potential effectiveness of mHealth training and NCD and primary care delivery.

Results: Nurses who received mHealth training recorded a statistically significant difference ($p<0.001$) in average pre-and post-test training scores of 65.2 (±12.2) and 86.5(±7.9) respectively. Recordings on treatment cards indicated appropriate diagnosis and follow-up of hypertensive patients with significant improvements in systolic blood pressure ($t=5.09$, $p<0.001$) and diastolic blood pressure ($t=5.07$, $p<0.001$). The mHealth nurse training and WhatsApp support groups were perceived as valuable experiences and obviated the need for face-to-face training. Increased workload, non-availability of medications, facility-level conflicts and poor task shifting were identified challenges.

Conclusions: This initiative provides evidence of the feasibility of implementing an NCD care package supported by mHealth training for health workers in PHCs, and the strong possibility of successful scale-up nationally.

Article Summary

Strengths and limitations of this study

- Pragmatic implementation of mHealth training and support model based on a country-specific (WHO PEN compatible) NCD package of care.

For peer review only - http://bmjopen.bmj.com/site/about/guidelines.xhtml

- Successful process evaluation of feasibility, implementation experience, challenges and patient satisfaction using quantitative and qualitative methods.
- A decline in patient numbers attending facilities imposed by the COVID-19 pandemic and the shortened pilot period led to a reduction in numbers screened, diagnosed, started on treatment and evaluated.
- Perception of the initiative predominantly as a non-governmental organisation (NGO) pilot rather than an obligatory health system strengthening initiative driven by the relevant authorities (State and Federal Ministries of Health).

Introduction

Non-Communicable Diseases (NCDs) are responsible for the deaths of 41 million people annually, with an estimated 15 million of these deaths occurring in people between 30 and 69 years of age.[1] Among NCDs, the four top conditions that together account for more than 80% of all these premature deaths include cardiovascular diseases (17.9 million deaths annually), cancers (9 million), respiratory diseases (3.9 million) and diabetes (1.6 million). Almost three-quarters of NCD-linked deaths occur in low-and middle-income countries (LMICs). Fragile health systems experience challenges in controlling both NCDs and infectious diseases such as HIV/AIDS and tuberculosis (TB). The rapid global epidemiologic transition and rising rates of NCDs are also having negative effects on the health of Nigerians.

In Nigeria, NCDs are estimated to account for 29% of all deaths,[2] with 20% dying prematurely.[3] Community-based surveys involving adults in Cross River State Nigeria have identified that approximately 7% of residents in Calabar had undiagnosed diabetes mellitus[4], 42% and 18% had hypertension and obesity respectively.[5] It is projected that by 2030, NCDs will be the leading cause of morbidity and mortality in Nigeria.[6,7] This rise in NCDs has been attributed to rapid population growth and urbanisation, limited access to healthy and nutritious food and physical inactivity.[8,9]

The fragile Nigerian public health system is not well equipped to effectively manage and control NCDs.[10] Until 2019, NCD care was not prioritised by policy-makers. For example, only 3.3% of the national health budget is devoted to mental health care with fewer than 150 mental health specialists serving Nigeria’s estimated 206 million population.[11] In 2019, Nigeria’s Federal Ministry of Health launched the National Multisectoral Action Plan for the Control and Prevention of NCDs[12], prioritising health system strengthening at all levels of care toward universal health coverage (UHC). A major thrust of the plan was the integration of NCDs management into primary healthcare (PHC) services. Despite this, the recently launched National task-shifting and task-sharing policy does not cover NCD prevention and control.[13] NCDs are not prioritised in health worker basic or post-basic training, as in many other countries. So far, the PHC facilities in Nigeria have not been involved in programmatic/systematic care for NCDs (or other chronic diseases except for TB and HIV).

For peer review only - http://bmjopen.bmj.com/site/about/guidelines.xhtml

The Communicable Disease Health Service Delivery (COMDIS-HSD) programme has developed care guideline processes to identify, diagnose and treat NCDs. These packages of care have been adapted and used at a provincial scale in Pakistan, Swaziland[14] and elsewhere with higher rates of diagnosis and NCD control in the primary care facilities.[15-20] Since 2017, this NCD package has been scaled-up across the outpatient departments of all public hospitals and many health centres in the Punjab Province, resulting in the diagnosis and treatment of 540,000 people.[16-20] Similarly, the COMDIS-HSD NCD package has been adapted and adopted in Sierra Leone[21] and in 71 rural health facilities in China, covering 2.5 million people.[22]

The COMDIS-HSD NCD package has been also adopted by the Nigerian Federal Ministry of Health (FMoH) NCD technical working group, with technical support from the Nuffield Leeds, United Kingdom (UK). The package is technically compatible with the WHO Package for Essential Noncommunicable (PEN) disease interventions and provides steps of medication and patient education (https://comdis-hsd.leeds.ac.uk/resources/ncd-care-package/). The PEN/COMDIS-HSD package has been piloted in its hard copy residential course format by the FMoH/NCD division in Abuja Federal Capital Territory. However, there is no funding for scaling-up these traditional in-class service courses for health workers at a national level.

By leveraging the high mobile phone penetration in Nigeria, we aimed to pilot the use of an innovative mobile health (mHealth) user-friendly view-through rate (VTR) mobile training application that can be used on any smart phone, tablet or computer both online and off-line. The guide and training modules were adapted for mHealth online delivery by Instrat Global Health Solutions (an indigenous technology company) with pre-post tests and award of continuing professional development certificates. We hypothesised that mHealth NCD training and a support group for nurses should lead to improved knowledge and improved competencies in the screening/diagnosis, treatment initiation and optimal blood pressure control in patients at PHC level. Our overarching goal was to develop a workable and sustainable approach to support health workers in managing NCDs at the PHC level which can be scaled-up across Nigeria.

Methods

Patient and Public Involvement: The development of the research question and outcome measures in this initiative were directly informed by Nigerian patients' priorities, experience, and preferences. These priorities were factored into the design of this study. Patients were not directly involved in the recruitment to and conduct of the study but were beneficiaries of the NCD package of care we provided in the PHCs. We aim to disseminate the results of this initiative by conducting interactive sessions with the patients who continue to receive care for their NCDs in the PHCs in Cross River State.

For peer review only - http://bmjopen.bmj.com/site/about/guidelines.xhtml

Implementation package

The NCD desk guide, training modules, and treatment card record package were adapted from the WHO PEN guidelines by our team (http://comdis-hsd.leeds.ac.uk) in collaboration with WHO Nigeria and the Federal Ministry of Health. In Nigeria, registered nurses, who are qualified health workers at PHC facilities, have not been trained to provide NCD prevention and treatment. The NCD package had been initially piloted in conventional face-to-face training at a venue in the Federal Capital Territory Abuja with WHO Nigeria funding. However, there was no funding for scaling-up this package across the states and thousands of PHC facilities. Online mHealth training and support have the potential for large-scale replication.

Research design

In this pilot, we adopted a mixed-methods approach to:

(i) Evaluate changes in NCD related knowledge through pre-post training assessments included with the online training.

(ii) Understand service delivery and implementation contextual factors through structured observations and focus group discussions involving the health workers following training.

(iii) Assess outcomes through record review of treatment cards for appropriateness of screening, diagnosis and initiation of treatment and changes in blood pressure etc.

The implementation of this pilot was guided by the proposed theory of change that recognises the various inputs and activities expected to generate patient-related outcomes and health systems impact driven by the policy change. The theory is underpinned by contextual factors such as the current organisation and resourcing and limitations of the health system; limitations placed on the scope of tasks performed by health workers at PHCs; availability of funding to train health workers and the opportunity to leverage mobile phone penetration. It was not possible to involve patients or the public in the design, conduct, reporting or dissemination plans of our research. Table 1 presents the logic model guiding the implementation of the pilot. The model captures the various inputs for this project including the case management and lifestyle counselling desk guidelines, the mHealth training, supervisory visits, treatment cards and relevant equipment. The model also reflects the actions undertaken in the course of this project, i.e., screening for NCDs, prescription of relevant drugs, counselling on a healthy lifestyle, focus group discussions and follow-up care. These actions are then linked to intended practice changes, outputs and health outcomes.

For peer review only - http://bmjopen.bmj.com/site/about/guidelines.xhtml

Intervention inputs	Intervention process & actions	Intended		
		Practice change	Outputs	Health outcome
• Case management desk guideline, including lifestyle counselling<br>• mHealth training of nurses on the deskguide package<br>• Nurse WhatsApp support group<br>• Supervision of care visits (quarterly)<br>• Digital BP apparatus, glucometer & strips<br>• Treatment card, used for clinical care [subsequently also as research data records]<br>• Monthly funds for health workers' telecommunication fees	• Screen/ diagnose<br>• Prescribe anti-hypertensive<br>• Identify hypertension and/ or diabetes and treat<br>• Counsel for lifestyle modification<br>• Follow-up care<br>• Focus group discussion with nurses, month 5	Provider practice:<br>• Screen, diagnose, treat, counsel, follow-up & report as per the case management desk guideline<br>Patients practice:<br>• Follow-up visits<br>• Treatment<br>• Lifestyle changes as counselled e.g. healthy eating, activity, smoking cessation	Patients are:<br>• Screened & diagnosed as per desk guide<br>• Prescribed right drug/ dose<br>• Counselled for life-style change<br>• Followed-up and treated – i.e., continuing care	• BP trend by visit [primary outcome].<br>• Hypertension and/or diabetes care recorded on the NCD card.

Setting and participants

This pilot was carried out in a total of 19 PHC facilities in Cross River State, South-Eastern Nigeria over 12 months between February 2020 and January 2021. It involved 23 nurses drawn from selected sites in rural areas and peri-urban areas in Cross River State. Purposive sampling was used to choose the 19 PHC facilities; this was done in collaboration with the Cross River State Primary Health Care Development Agency (CRSPHCDA). The CRSPHCDA is a government agency tasked with ensuring 70% of the population in CRS have access to affordable quality healthcare at the primary care level. The CRSPHCDA proposed 23 nurses

For peer review only - http://bmjopen.bmj.com/site/about/guidelines.xhtml

and one Community Health Extension Worker (CHEW) from the 19 PHC facilities for the NCD training based on the number of available nurses per facility. On the average, there is one nurse per PHC facility. It was spread across all the senatorial zones and local government areas with the aim of sampling from at least 10% of the just over 190 larger PHCs (staffing including at least one registered nurse) in Cross River State.

Process

A one-day face-to-face orientation was organised in Calabar, the capital of Cross River State, in February 2020. This introduced 24 health workers to the NCD training package, giving them hard copies of the desk guide, job aide, treatment cards and showed them how to access and use the mHealth training modules for self-study.

The mHealth modules were developed using the *MediXcel Lite* health technology platform which has been previously described.[23,24] The training modules consisting of video and text-based learning materials were created in English which was understood by all health workers. Several facilitation techniques were used by the trainers such as practical sessions, group discussions and case studies. All health workers were then expected to complete the eight NCD modules over a two-week period by accessing the NCD materials on their mobile phones and tablets. User logins were created for the health workers by the staff of InStrat to enable them to download the App onto their android mobile phones. This App also tracked their progress with the NCD modules automatically. The maximum possible score was 100% with the passing score set at 70% for each module. Continuing professional development certificates were awarded to health workers who completed the NCD modules.

Following this orientation and mHealth training, the health workers were expected to utilise the information contained in the modules to screen and identify patients presenting with NCDs to their various facilities. Treatment cards were distributed, and participants were instructed to upload monthly updates on the number of NCD cases seen in the various facilities via an electronic *Comm Care* App portal on mobile electronic devices. Relevant clinical materials such as sphygmomanometers, glucometers and test strips were provided to facilitate the clinical assessment of clients with NCDs.

The health workers were provided with funds monthly to cover telecommunications fees. A community of practice (CoP) WhatsApp group was created for the health workers to facilitate peer-to-peer interactions, real-time patient care support by clinicians within the research team and data management by IT personnel of the implementation team. The WhatsApp group also served as an avenue to provide clinical support and data recording support. The health workers who uploaded their facility data onto the *Comm Care App* on time were openly commended as a form of non-financial motivation and as a reminder to others to do the same.

Supportive supervision was provided to the health workers in the form of weekly discussions, WhatsApp phone calls and supplemented by quarterly facility clinician

For peer review only - http://bmjopen.bmj.com/site/about/guidelines.xhtml

supervisory visits. The facility visits involved first-hand assessments of the service delivery contextual factors. During these trips, clinical mentoring was provided, and the health workers were observed providing care to patients using the clinical desk guide and treatment cards. Feedback was obtained from the health workers during these sessions.

Responding to the early implementation issues identified by the implementation team, a new module on integrating NCD management into the daily activities of PHC facilities was developed and deployed on the training App.

Focus group discussions

In the 5th month from the start of implementation, focus group discussions (FGDs) were conducted as part of the research, with the health workers (see supplementary file). Short message service invitations were sent to 24 participants who completed the intervention to share their perceptions of the digital training and experiences in focus group discussions. Focus groups (FG)[25] were the chosen method as they are useful for exploring perspectives and encouraging reflections on experiences. Twenty-three participants accepted the invitation. We conducted four focus group sessions over 10 days, with an average of 6 participants in each group (FG1 n=8, FG2 n=4, FG3 n=5, FG4 n=6). There were only two male participants, and they were part of the fourth group.

Each FGD was facilitated by 2 researchers experienced in conducting FGDs and an assistant. The research assistant coordinated the session schedules, recording and served as the official note-taker. Sessions were 1 – 2 hours in length and conducted face-to-face in the place convenient for participants. Open-ended questions, formulated by the team who have formal training in qualitative research were used to gain the best perspective and scope of individual responses on (1) perceptions of the use of m-Health training strategy, (2) experiences with the implementation of clinical guidelines (3) reflections on the adequacy of received supervisions, (4) reflections on potential scale-up of the intervention. Facilitator notes ensured uniformity among FGDs and kept each discussion on target. At each FGD, an opportunity was given to withdraw consent for participation and recording. Responses were recorded, and participants were assured their comments would be kept confidential and anonymous.

No formal sample size was calculated for this pilot study.

Ethical approval for the study was received from the Cross River State Health Research Ethics Committee CRSMOH/RP/REC/2019/173. Informed written consent was obtained from eachhealth worker participant and oral consent from the patients.

Data analysis

Quantitative data

The screening, diagnosis, treatment and follow-up care are specified in the NCD guide, and this information is recorded on the treatment card. The record review was of all patients

For peer review only - http://bmjopen.bmj.com/site/about/guidelines.xhtml

identified and initiated on care. The assessment was of quality care according to these guidelines and the completeness of recording of key clinical measurements of blood pressure (BP), body mass index (BMI), fasting blood sugar (FBS) and urine analysis (as per guideline for that disease) were metrics used to assess the effectiveness of the pilot project. We assessed the effect of the NCD mHealth training through the pre/post-test results and assessed operational issues that may have contributed to the results. The overall assessment was the degree to which the NCD mHealth training and care was feasible, sustainable, replicable, and treatment initiation quality was (as per guideline). Phone interviews were conducted on a random sample of patients across all facilities using a brief guide that included questions on satisfaction.

Data were imported and analysed in SPSS v23.[26] Descriptive analysis of quantitative data was carried out using means, standard deviations, frequencies, and percentages. Group differences were established using chi-square analysis. Descriptive trend analysis for the follow up rates in the systolic blood pressure and diastolic blood pressure changes in the hypertensive patients was conducted on the monthly basis. The paired t-test was used to compare the average change in systolic blood pressure and diastolic blood pressure levels at baseline and three-month follow up among the hypertensive patients. Hypothesis testing was two-tailed, at the 5% level.

Qualitative data

We used qualitative thematic analysis to identify prominent themes and patterns in the data and included a combination of deductive and emergent strategies. The facilitators debriefed immediately after each FGD. Two researchers (AO and SA) independently reviewed the data and came together to discuss emerging ideas. After the discussion, the researchers returned to the data to further review and confirm coding. This iterative process resulted in grouping the codes into three major themes: (1) perceptions with the mHealth trainings, (2) experiences with the implementation of NCD care packages, (3) potential issues with the intervention scale-up. Qualitative data were analysed using Dedoose software.[27]

The development of the research question and outcome measures in this initiative were directly informed by Nigerian patients' priorities, experience and preferences. These priorities were factored into the design of this study. Patients were not directly involved in the recruitment to and conduct of the study but were beneficiaries of the NCD package of care we provided in the PHCs. We aim to disseminate the results of this initiative by conducting interactive sessions with the patients who continue to receive care for their NCDs in the PHCs in Cross River State.

Results

A total of 24 health workers from 19 primary health centres (PHCs) across the state were trained using the App. All the participating health workers achieved the passing score of

For peer review only - http://bmjopen.bmj.com/site/about/guidelines.xhtml

70% for each module, enabling them to progress to the next module. There was no limit on the number of attempts that could be made on the post-module test, and all the health workers were able to complete the modules in the given two weeks. The average pre-test and post-test scores were 65(±12) and 87(±8) respectively with a statistically significant difference between these scores following a two-tailed t-test ($p<0.001$).

Since the project initiation, 414 (72.7%) community members of the 569 patients seen were newly diagnosed cases of NCDs. The rest were patients who had previously been diagnosed with NCDs prior to the onset of the project. The average age of males diagnosed with an NCD was 53 years (SD= 15) and for females 49 years (SD=14). Over three-quarters (n=432, 76%) of all cases and over half (n=315, 55%) of newly diagnosed cases were hypertensive patients (newly diagnosed:110 male, 205 female, average age 52, SD=13) and 14% were diabetics (15 male, 35 female, average age 49, SD=14) (Table 2).

Fasting blood sugar checks were conducted on 215 of the community members with 47 (22%) of them falling within the prediabetes range of 5.6-6.9mmol/l and 82 (38%) falling above the diabetes threshold of >7.0mmol/l (Table 2). Proteinuria and/or glycosuria was identified in 14 of the 113 persons who had a urine analysis. In accordance with the NCD guideline, fasting blood glucose was only done if the person was overweight or had related symptoms, and urine analysis was only done as indicated.

Table 2. Characteristics of NCDs patients served by targeted PHC clinics n=569

**Characteristics**	**Male (n=206/36%)**	**Female (n=363/64%)**	**Total (n=569)**
Age (μ ± SD)	53.5±15.2	49.75±14.2	51.8 ±14.2
**NCD patients who attended facilities**			
Hypertension	155 (75.2%)	277 (76.3%)	432 (75.9%)
Diabetes	23 (11.2%)	47 (13.0%)	70 (12.3%)
Asthma/COPD	10 (4.9%)	17 (4.7%)	27 (4.7%)
Depression	1 (0.5%)	8 (2.2%)	9 (1.6%)
Sickle cell anaemia	6 (3.0%)	1(0.3%)	7 (1.2%)
Epilepsy	3 (1.5%)	3 (0.8%)	6 (1.1%)
Other NCD	8 (3.9%)	10 (2.8%)	18 (3.2%)
Total	206 (100%)	363 (100%)	569 (100%)
**New diagnosis of NCDs**			

For peer review only - http://bmjopen.bmj.com/site/about/guidelines.xhtml

Yes	145 (70.4%)	269 (74.2%)	414 (72.7%)
No	61 (29.6%)	94 (25.8%)	155 (27.3%)
Total	206 (100%)	363 (100%)	569 (100%)
**Check-up items**			
Height (μ ± SD)	1.56±0.14	1.56±0.11	1.56±0.12
Weight (μ ± SD)	67.4±12.6	68.5±12.4	68.1±12.5
**BMI**			
Normal 18.5-24	57 (27.7%)	114 (31.4%)	171 (30.0%)
Overweight 25-30	78 (37.9%)	124 (34.1%)	202 (35.5%)
Obese ≥30	62 (30.1%)	122 (33.6%)	184 (32.3%)
Total	206 (100%)	363 (100%)	569 (100%)
**Blood pressure (BP)**			
Normal BP 90/60mmHg-120/80mmHg	52 (25.2%)	73 (20.1%)	125 (22.7%)
High normal 130/85mmHg - 139/89mm Hg	21 (10.2%)	38 (10.5%)	59(10.4%)
High BP ≥140/90mmHg	133 (64.6%)	252(69.4%)	385 (67.7)
Total	206 (100%)	363 (100%)	569 (100%)
**Diagnostic tests**			
**Fasting blood sugar (FBS)**			
Normal reading <5.6mmol/l	30 (40.0%)	56 (40.0%)	86 (40.0%)
Prediabetes 5.6-6.9mmol/l	15 (20.0%)	32 (23%)	47 (21.9%)
Diabetes reading >7.0mmol/l	30 (40.0%)	52 (37.1%)	82 (38.1%)
Total	75(100%)	140 (100%)	215 (100%)
**Urine analysis**			
Normal	32(69.6%)	57(65.5%)	89 (66.9%)
Proteinuria and/or glycosuria[§]	14 (30.4%)	30(34.5%)	44 (33.1%)
Total recorded	46(100%)	87(100%)	133 (100%)

For peer review only - http://bmjopen.bmj.com/site/about/guidelines.xhtml

[§]Proteinuria- proteins in urine, glycosuria-sugar in urine. According to the NCD guideline, fasting blood glucose taken if overweight or have related symptoms, and urine analysis as indicated.

Gender distribution of newly diagnosed NCD cases (Figure 1) was not statistically different $\chi 2$ (6) =8.4, p=0.2. On average, 16 hypertensive and 3 diabetic patients were identified per facility (range 3-38 for hypertensive and 0-12 for diabetic patients).

Figure 1. Gender distribution of newly diagnosed NCD cases

All NCD patients (n=569) registered at targeted facilities received height (μ=1.56, SD=0.12) and weight measurements (μ=68.1, SD=12.47) at the first appointment. BMI was calculated and recorded for all of these patients (μ=28.1, SD=5.85). Over forty percent of NCD patients (n=234, 41%) received an appointment reminder following the screening appointment.

Healthy lifestyle advice

In all, 356 (62.6 %) patients were given lifestyle advice such as reducing/ stopping cigarette smoking and alcohol, reducing weight, reducing salt intake, increasing physical activity/ exercise, reducing stress, medication adherence, healthy eating, and healthy living. For these a motivational counselling strategy was used.

Primary outcome

Following the initial screening visit involving 420 patients, the patients were followed up with 230 of them making two such follow-up visits. A total of 98 patients made up to three follow-up visits (Table 3). Recordings on treatment cards indicated a positive significant improvement in blood pressure of hypertensive patients from visit 1 through to visit 3 (Figure 2). The systolic blood pressure for hypertensive patients who had three follow-up visits (after the initial screening visit) steadily decreased from the visit 1 average of 162 to a visit 3 average of 139 mmHg. The average systolic blood pressure decreased 23 mmHg between the screening to follow-up visit 3 (p<0.001). A similar trend was registered in a decrease of 12mmHg in diastolic blood pressure from the follow-up visit 1 average of 93.8mmHg to follow-up visit 3 average of 81.7mmHg (p<0.001).

Figure 2: Trends in blood pressure change among hypertensive patients (n=428)

Table 3: Trend on blood pressure from visit 1 to visit 3 based on the average of two measurements

Parameter	Value	All patients (screening visit)	patients with 2 visits		patients with 3 visits	
			Visit 1	Visit 2	Visit 1	Visit 3

For peer review only - http://bmjopen.bmj.com/site/about/guidelines.xhtml

Systolic BP (mmHg)	Valid values	420	230	230	98	98
	Mean (±SD)	159.9 (23.3)	160.3 (20.7)	143.2 (20.6)	162.1 (21.4)	139.3 (19.1)
	Range	90-270	90- 220	99-218	90- 220	80-180
	Values >140 (%)	77.6%	79.6	43%	83.7	32.3%
	Values >180 (%)	15.0%	14.8	3.9%	16.3	--
Diastolic BP (mmHg)	Valid values	420	230	230	98	98
	Mean (±SD)	96.0 (14.3)	95.3(13.5)	85.5 (12.3)	93.8 (15.3)	81.7 (11.1)
	Range	50-174	50- 152	80-130	50- 152	50-120
	Values >90 (%)	60.7%	59.1	22.6%	53.1	13.3%
	Values >110 (%)	10.2%	7.8	3.9%	9.2	1.0%

Compliance with medication

The hypertensive patients were prescribed antihypertensive drugs from the following classes: (1) Angiotensin-converting enzyme inhibitors (lisinopril, captopril), (2) Calcium channel blockers (nifedipine, amlodipine), (3) Diuretics (furosemide, hydrochlorothiazide) which were all in line with recommendations provided in the NCD deskguide. Where hydrochlorothiazide was unavailable, normoretic (i.e. amiloride hydrochloride 5mg and hydrochlorothiazide 50mg) was prescribed. The top three most often prescribed antihypertensive drugs were normoretic (50%), nifedipine (44%) and amlodipine (33%). Diabetic patients were predominantly prescribed metformin, glibenclamide or insulin injections.

Patient satisfaction

Patient satisfaction with nurses and clinical interaction was assessed based on patient exit interviews with 29 randomly selected NCD patients (17 hypertensive, 5 diabetic, 6 comorbid, 1 asthmatic) within two weeks of their last appointment. Twenty-three (79%) patients perceived they were being treated by nurses with respect. Three patients commented on long waiting hours and lack of staff in facilities. Nearly all interviewed patients (n=25, 86%) agreed that nurses did a good job with explaining concepts and providing information on lifestyle advice and medication adherence in a clear and

For peer review only - http://bmjopen.bmj.com/site/about/guidelines.xhtml

understandable manner. Satisfaction with the quality of care and treatment provision was also high (n=23, 79%). Most patients (n=24, 83%) expressed willingness to continue accessing facilities to receive NCD care; five patients, however, commented they would consider that only if the provision of care improves. When asked about challenges encountered during the period of clinic visits, 8 patients described no barriers, whereas two thirds of the patients (n=19, 66%) described at least one barrier. Lack of money to buy the prescribed medication was the most frequently cited barrier (Figure 3).

Figure 3. Challenges encountered by patients during the period of clinic visits

Focus group discussions

Participants

Focus groups included 21 women and 2 men, between 31 to 60 years of age. All participants had post-secondary training in nursing. Some of the participants had prior experience of attending digital training. However, NCDs had not been prioritised in the health worker basic or post basic training the participants had previously received.

Perceptions of m-health training

Content

The mHealth training was stated to be a valuable experience. Participants were very appreciative of the knowledge they acquired about prevention and control of NCDs. They talked about improved confidence and an increased sense of empowerment in their ability to provide counselling on lifestyle behaviours and medication adherence. This positively influenced their internal sense of importance, consistent with improved self-efficacy.

> *"Some of these cases … before now I didn't know how to handle them but now I am very confident in handling the issues. The way and manner I am able to regulate clients' blood pressure are making me feel important" [FG1].*

> *"I will begin by appreciating the NCD project and people. Before now, we actually didn't know how to handle these cases but now we are very confident. We know the particular drugs to prescribe and the dosage". [FG2]*

Functionality

Participants perceive the functionality of m-health technology and ease of use as important. Multiple benefits of online learning were noted including the ability to learn at your own pace, the opportunity to return to the material when needed, using smart phones to access on the VTR the NCD deskguide and modules.

For peer review only - http://bmjopen.bmj.com/site/about/guidelines.xhtml

"We are upgraded. It makes the provider more educated, because you read at your pace, you don't stress yourself, anytime you come back you read and understand. So.. I think it was great."[FG1].

"It was a nice one. Almost all the trainings we have attended have not been this type. It has made us to know how to use our phone in learning online and also searching for other things you would not have ordinarily done because of the workload where we find ourselves. It is a good one. Many times, we tell ourselves let's go online something is happening "[FG4].

For peer review only

Positive impact

Participants talked highly about the positive changes the integrated care delivery brought to the communities they served. Provision of BP and blood glucose diagnostic equipment meant targeted diagnostic and curative services delivery at a point of access.

"The NCDP [the project] has made us have the necessary equipment to carry out blood pressure, diabetes diagnosis in my facility. This is a great achievement really. People come in now to say I want to check my blood pressure and sugar." [FG4]

When people come to our ANC, we have some of the test kits to use and we started testing. We admitted some people. When people come to visit them, they go back to the community to tell others. Presently, we have increased client flow. It is helping us to detect these NCDs". [FG4]

The provision of PHC facility services dramatically improved relationships with communities and encouraged follow-up visits. During outreaches, health workers were able to use the donated sphygmomanometers to measure blood pressure within the communities and refer people to the PHC facility.

"The relationship with the community as a result of the NCD is marvellous wherever we meet with these clients they greet one with respect and are open to me" [FG1]

"Giving the test that was not existing in the community before really help to mobilize the people. They feel more relaxed and confident to come to the facility".[FG4]

Participants described a variety of community education strategies to raise awareness of the availability of NCD services. Some strategies involved monetary reward for spreading the word about services by the town crier, and also use of local worship places and/or community organisation to engage with communities.

For peer review only - http://bmjopen.bmj.com/site/about/guidelines.xhtml

> *"…I have a relationship with the town announcer. Although when people come and take the test, we collect a token from them, and I use that to pay the town announcer. I type what I want him to announce. He reads through it and announces it to the people. From there people started coming". [FG4]*

> *"This project has impacted on our knowledge, and we can confidently do health talk and create awareness about blood pressure, and other NCDs efficiently. My wish is.., I hope this project stays, honestly." [FG1]*

Challenges

Participants, however, also experienced, or perceived challenges to the implementation of care packages as extending across clinical, organisational and societal levels.

Challenges at the clinical level

Factors identified as challenges at the clinical level included increased workload associated with (a) ensuring the continuity of NCD care delivery, and (2) filling in the treatment cards. Participants noted the NCD initiatives' success meant the increased flow of patients to facilities for check-ups and follow-up consultations. Although this has generally been perceived as a positive dynamic, the increase in workload was mentioned.

> *"NCDs increased our work. Before now, we see them and they go away, we don't bother to follow them up or they don't come back to us to maintain their BP. We need to maintain them to get their drugs or make sure their blood sugar level got controlled. Some of us, our facilities are easy to get to, so they can easily come. Even if you are resting, they want you to come and check them. When they heard that someone in the community died due to high blood pressure, that period a lot of people came for checking [FG4].*

Some participants also noted that filling the treatment cards and registers was time-consuming and often discouraged their use. Content reduction was a proposed solution to address this.

> *"Ordinarily we have few questions to capture from clients but the NCD has widened a lot of information that needs to be captured, looking at this with other registers in the facility too has made the workload high, and again there are series of tests that also needs to be run on an individual basis." [FG1].*

Technical glitches in the application and internet connection were also noted resulting in issues with uploading data into the central system. This resulted in double entry of data which was perceived as a double burden on staff. Some participants also recognised that

For peer review only - http://bmjopen.bmj.com/site/about/guidelines.xhtml

supervision and monitoring provided by staff of the initiative helped them navigate many challenges along the way.

Challenges at the organisational level

The presence of professional support or opposition was highlighted as a considerable factor for the successful integration of the care packages into daily routine. Participants talked at length about conflict of interest between the focal person responsible for NCD care and PHC facility in-charge. Prior to the training, there was no focal person responsible for NCD in these facilities. Following the NCD training, the 23 nurses were encouraged to assume the role of NCD focal person in their respective facilities. The PHC facility in-charges were typically registered nurses who also doubled as the administrative head of the health facilities. Examples of interference from *person-in-charge* into modes of care delivery, equipment storage and use as well as generated income distribution were noted.

> *"The thing is really disturbing because the in-charge kept telling you that the equipment is not for you and should be kept in the laboratory, giving unnecessary orders before you can make use of it. It pissed me off a lot." [FG1].*

Some participants, on the other hand, talked positively about PHC facility in-charge and the support they received. They also acknowledged how some managers actively "champion" the awareness-raising activities amongst communities, and how this considerably increased the flow of patients to facilities.

> *"The new in charge has leadership skills. He went to the community heads and introduced himself, he went to churches. He will encourage sick people to always come and test. A lady died and it was discovered her blood pressure was high. So, they announced in all the churches that people should go and check their blood pressure and sugar level. This really helped us. [FG4]*

Although many participants talked in terms of conflict, in general, negotiation, avoiding confrontations and/ or for managers undergoing training on NCD care alongside junior staff were preferred approaches to resolving the problems.

> *"I believe they should train them. Since we have this knowledge, they know some of the drugs, but they don't know the right time to administer. If they are exposed to this training and they are told the stages and when to prescribe, they will know what to do. For example, in my facility my in-charge prescribed stage II drugs instead of stage I, I had to correct him. If they know, they will do better." [FG4]*

For peer review only - http://bmjopen.bmj.com/site/about/guidelines.xhtml

Participants also noted negative changes in the spirit of collegiality and the need to ensure that *“the focal persons”* have the expertise to manage NCD cases. This point reflected the need for such training to be stepped down to other staff who may not have attended the initial face-to-face orientation meeting.

Challenges at societal level

The restrictions to social interaction that followed the emergence of COVID-19 substantially limited the number of individuals attending the pilot PHCs for care. This had a negative impact on the number of persons who were eventually screened for NCDs. Also, some civil unrest occurred during the course of the project as part of the nationwide protests which resulted in vandalisation of some of the pilot PHCs. This halted services in the affected PHCs for several weeks and scaled back their recruitment rates after they reopened.

Potential issues with the intervention scale-up

Participants talked about pressing issues in a healthcare system that potentially can have a negative impact on the scale-up of the intervention. The most often mentioned challenges identified were health personnel shortage (especially nurses), lack of knowledge on NCD management and control, lack of essential diagnostic equipment and medication necessary for effective NCD care delivery.

Potential key solutions for ensuring the sustainability and potential scale-up of care packages at a national level were (a) targeted training of other staff on NCD prevention and management, and (b) task-shifting activities for NCD care. Participants also noted that staff at primary care level lacked basic knowledge on NCD care and if left at their own disposal many essential diagnostic tests will be missed. Therefore, they will be willing to provide training to junior staff to ensure effective delivery of services.

> *“Yes…, because some things, if left for the facility to carry on, will not be effective, but with a focal person she will ensure things are in order, since they know they will be held responsible” [FG1].*

> *“Yes, like in my facility I am the only staff I will appreciate if another staff is sent here so that we can have more hands, and I will give the person on the job training or some kind of step-down training to enable whoever is coming to render the services. It will be bad if this NCDP [the NCD project] stops, before now we have had cases where somebody will just fall while walking. The next thing you hear is people will be saying he is a ghost, not knowing is the effect of high blood pressure. So,…. I really don’t want this NCD thing to just fade like that, I don’t know how to put it really” [FGD2].*

For peer review only - http://bmjopen.bmj.com/site/about/guidelines.xhtml

Discussion

Main findings

In this mixed-methods pragmatic implementation of health worker training using a mHealth platform complemented by clinical supervision visits and a community of practice forum, we have demonstrated the feasibility of delivering training remotely leading to improvement in knowledge and skills in NCD screening, diagnosis, and treatment. In addition, we have documented nurses' experiences, perspectives, challenges the feasibility of task-shifting to nurses as well as patient satisfaction and challenges experienced with the care provided. This cohort of patients was found to have improved trends on average control of blood pressure. There was a statistically significant reduction on both systolic and diastolic blood pressures between the first and third visits among hypertensive patients. Nonetheless, the results do not prove a causal relationship between the mHealth training intervention and the resultant trend in blood pressure. An experimental study with a control arm where health workers not involved in the study offered routine PHC level care at separate facilities would have provided necessary data for comparison. This was not possible in this study. Diuretics and calcium channel blockers (nifedipine and amlodipine) were the most commonly prescribed antihypertensives with metformin and glibenclamide being the most commonly prescribed therapies for diabetes mellitus. These prescribing practices were all in line with the recommendations within the NCD guide.

Health education/counselling was given to about two-thirds of patients on all visits. The process indicators demonstrate the ability of nurses in PHC clinics to successfully deliver the key components of NCD care. The NCD guide was designed for qualified staff at the level of a 3-year trained registered nurse. The deployment of an mHealth platform rather than a face-to-face classroom style model to deliver the training has been shown to be feasible and effective. This is seen in the improved knowledge scores upon completion of the modules and assessment of skills seen in ability to initiate care for patients with NCDs.

The mHealth NCD training was perceived as a valuable experience with the health workers identifying multiple benefits of online learning and the integrated NCD care delivery package. This is important as there is not funding sufficient for face-to-face training (including per diems and venue cost) for nation-wide scale up of NCD training and supervision across Nigeria, as in other low-middle income countries there is no 'Global Fund for NCDs, as there is for AIDS, TB and malaria. We provided a one-day face-to-face orientation session to introduce the health workers to the mHealth platform and later supported them remotely to complete the modules online. This hybrid form of training appears to be suitable for the Nigerian context where face-to-face training of large numbers of health workers might not be feasible.

Challenges to the smooth rollout of this NCD package were the increased workload it generated, the technical problems with the internet connection and difficulty in securing the support of health centre in-charges for the project. Remote and periodic onsite

For peer review only - http://bmjopen.bmj.com/site/about/guidelines.xhtml

supervision by local doctors and the provision of the materials (i.e., BP equipment, glucometres) that were lacking at the facilities were additional strategies adopted to support this initiative. The availability of these materials along with the face-to-face orientation, provision of monthly funds for health workers' telecommunication fees and hard copies of the training materials contributed to the overall success of the pilot. Therefore, such extra provisions will need to be taken into consideration when scaling-up this NCD package. Considerations such as appropriate diagnosis, quality of care and linkage to continuing care will need to be further addressed using a health systems framework that deals with these challenges more comprehensively.[28]

While the pilot appears to be successful, the sustainability and replicability of this model of training and care initiation will depend on addressing several health system barriers such as appropriate staffing (not all facilities have a nurse), provision of medicines and diagnostic equipment, improving on the governance system at the facility level and formally task shifting to nurses the NCD care and prescribing. In addition, the reference to the initiative as a 'project' by nurses implies a risk to sustainability, if the NCD initiative is not wholly adopted by the state health management. Despite its implementation through the routine system by a local medical NGO, the perception of it as a 'project' may undermine the long-term sustainability at the facility level. This mixed-methods research adds to the evidence base of similar NCD interventions in resource-limited settings. Using a similar shared care guide for management of diabetes and hypertension care in Eswatini (southern Africa), nurses were able to successfully deliver diabetes and hypertension care with improvements in physiological parameters, though continued training and supervision were required to consolidate change.[14] A similar NCD guideline and training COMDIS-HSD partner initiative in Pakistan was found to be effective in trials and was scaled across the Punjab Province of 110 million population.[16-20] This Nigeria pilot adds evidence for the potential in a West African setting and for mHealth as an alternative to expensive residential courses, which is a major constraint to NCD care scale-up.

Limitations

The emergence of COVID-19 and imposed public health restrictions substantially limited the number of individuals attending the pilot PHCs to be screened and diagnosed over an equally short period of ten months. Community mobilization was conducted by the trained nurses to help overcome some social barriers to accessing care that came in the wake of the COVID-19 pandemic. Some of the health facilities were vandalised during the nationwide protests that engulfed Nigeria in October 2020, and this also had a negative impact on the NCD care services. In addition, the pragmatic nature of our initiative precluded the provision of medicines, which may have led to an increase in NCD patients observed. Furthermore, given that many NGOs are operating in fragile health environments, pilot initiatives may not be subsequently implemented by ministries of health.

For peer review only - http://bmjopen.bmj.com/site/about/guidelines.xhtml

The observational nature of our study precludes us from drawing decisive conclusions as to whether care under the new NCD care model is better or worse than the alternative conventional residential training.

Conclusion

This pilot initiative provides evidence of the feasibility of implementing an NCD care package supported by mHealth training. The complimentary support provided for health workers in the form of supervisory visits, a community of practice forum and ancillary equipment facilitated their ability to screen for NCDs in the PHCs. The improvement in BP measurements in patients at follow up visits to the PHCs may be a reflection of the potential utility of this approach. These results support the implementation of such a package of care at scale within the Nigerian context and similar settings.

Acknowledgements: We appreciate the NCD division of the FMOH, Nigeria, Cross River State Ministry of Health and Primary Healthcare Development Agency as well as nurses and facility heads who participated in the pilot. We are grateful to all the patients who accepted to be managed using the NCD guides in the various health facilities in Cross River State Nigeria. We are thankful to Mr Tijesu Ojumu of the Nigeria Centre for Disease Control for designing the conceptual framework. Our sincere gratitude goes to the NIHR RUHF project of QMU Edinburgh for funding this initiative.

Contributorship statement: AO, EE, ObO and JW conceived the study. AO, EE, ObO, OO and CO were involved in the implementation of the programme. EE, ObO, OO and CO handled the data entry. SA, AO, ObO, JW and OO did the data cleaning and analysis. AO, SA, EE, JW and ObO wrote the first draft. All authors were involved in the reviewing and editing of this article and gave final approval of the version to be published. AO is the guarantor of the article.

Competing interests: None declared.

Funding: This study/project is funded by the UK National Institute for Health Research (NIHR) [NIHR Global Health Research programme (project reference 16/136/100)/ NIHR Research Unit on Health in Situations of Fragility]. The views expressed are those of the author(s) and not necessarily those of the NIHR or the Department of Health and Social Care. The funder played no role in the study design, data collection, analysis, interpretation, or in writing the manuscript.

Data sharing statement: Data generated by your research that supports your article will be made openly and publicly available upon publication of your article.

For peer review only - http://bmjopen.bmj.com/site/about/guidelines.xhtml

Patient consent for publication: not required

Ethics approval: This study was performed in line with the principles of the Declaration of Helsinki. The study received the approval of the Health Research and Ethics Committee of the Cross River State Ministry of Health Nigeria (Number/ID: CRSMOH/RP/REC/2019/173). Participation was voluntary.

Supplemental material: None supplied.

References

1. World Health Organization 2021. Noncommunicable diseases – key facts. Available: https://www.who.int/news-room/fact-sheets/detail/noncommunicable-diseases
2. World Health Organization - Noncommunicable Diseases (NCD) Country Profiles. Available: https://www.who.int/nmh/countries/nga_en.pdf?ua=1
3. World Health Organization [WHO]. Global health observatory data 2018: Premature NCD deaths. Available: https://www.who.int/gho/ncd/mortality_morbidity/ncd_premature_text/en/
4. Enang OE, Otu AA, Essien OE, *et al*. Prevalence of dysglycemia in Calabar: a cross-sectional observational study among residents of Calabar, Nigeria. *BMJ Open Diabetes Res Care* 2014; 2(1):e000032. doi: 10.1136/bmjdrc-2014-000032
5. Effa E, Onwusaka, O, Ekrikpo U, *et al*. Community based screening for risk factors of chronic kidney disease in Cross River State. *Kidney International Reports* 2020; 5(3):S58-S59.
6. Maiyaki M, Garbati M. The burden of non-communicable diseases in Nigeria; In the context of globalization. *Ann Afr Med* 2014;13:1–10.
7. World Health Organization. Noncommunicable diseases: the slow motion disaster. Geneva: WHO; 2017.
8. Crush J, Frayne B, McLachlan M. "Rapid Urbanization and the Nutrition Transition in Southern African." Urban Food Security Series No. 7. Queen's University and AFSUN: Kingston and Cape Town. 2011.
9. Maher D, Sekajugo J. Research on health transition in Africa: time for action. *Health Res Policy Syst* 2011; 9:1–4.
10. Green A. Country in focus: the growing burden of NCDs in Nigeria. *The Lancet Diabetes and Endocrinology* 2016; 4(1): 23-24
11. Ajayi T. What is Mental Health to an African? Available: https://www.linkedin.com/pulse/what-mental-health-african-tomiwa-ajayi
12. National multi-sectoral action plan for the prevention and control of non-communicable diseases (2019 – 2025). Available: https://www.iccp-portal.org/system/files/plans/NCDs_Multisectoral_Action_Plan.pdf

For peer review only

For peer review only - http://bmjopen.bmj.com/site/about/guidelines.xhtml

13. Federal Ministry of Health Nigeria 2014. Task-shifting and task-sharing policy for essential health care services in Nigeria. Available: file:///C:/Users/akaninyene.otu/Downloads/TSTS.pdf
14. Sharp A, Riches N, Mims A, *et al*. Decentralising NCD management in rural southern Africa: evaluation of a pilot implementation study. *BMC Public Health* 2020*;* 20:44
15. Walley J, Khan MA, Witter S, et al. Embedded health service development and research: why and how to do it (a ten-stage guide). *H Research Policy* & *Systems*. 2018; D-17-*00256R1*
16. Khan MA, Khan N, Walley JD, *et al*. Enhanced hypertension care through private clinics in Pakistan: a cluster randomised trial. *BJGP Open*. 2019; 3(1):bjgpopen18X101617.
17. Khan MA, Walley JD, Khan N, *et al*. Effectiveness of an integrated diabetes care package at primary healthcare facilities: a cluster randomised trial in Pakistan. *BJGP Open.* 2018;2(4):bjgpopen18X101618. doi:10.3399/bjgpopen18X101618
18. Khan N, Khan M, Walley J, et al. A process evaluation of integrated diabetes management at primary health care facilities in Pakistan. *BJGP Open*. 2018;2(4):bjgpopen18X101612
19. Khan MA, Khan N, Walley J, *et al*. How effective is it to deliver integrated COPD care at public healthcare facilities: a cluster randomised trial in Pakistan? BJGP Open 2019; 3 (1). bjgpopen18X101634. ISSN 2398-3795
20. Khan MA, Khan MA, Walley J, *et al*. Feasibility to deliver integrated COPD-Asthma care at primary and secondary level public healthcare facilities in Pakistan: a process evaluation. BJGP Open 2019; DOI: 10.3399/bjgpopen18X101632
21. Witter S, Zou G, Diaconu, K. et al. Opportunities and challenges for delivering non-communicable disease management and services in fragile and post-conflict settings: perceptions of policy-makers and health providers in Sierra Leone*. Confl Health* 2020; 14, 3. https://doi.org/10.1186/s13031-019-0248-3
22. Wei X, Walley JD, Zhang Z, et al. Implementation of a comprehensive intervention for patients at high risk of cardiovascular disease in rural China: A pragmatic cluster randomized controlled trial. *PloS One* 2017; 12 (8). e0183169.
23. Otu, A, Okuzu, O, Ebenso, B, et al. Introduction of mobile health tools to support COVID-19 training and surveillance in Ogun State Nigeria. *Front Sustain Cities* 2021; 3: 10.
24. Otu A, Okuzu O, Effa E, et al. Training health workers at scale in Nigeria to fight COVID-19 using the InStrat COVID-19 tutorial app: an e-health interventional study. *Therapeutic Advances in Infectious Disease* 2021. doi:10.1177/20499361211040704
25. Willis K, Green J, Daly J, *et al*. Perils and possibilities: achieving best evidence from focus groups in public health research. *Aust N Z J Public Health.* 2009;33(2):131–6
26. IBM Corp. Released 2015. IBM SPSS Statistics for Windows, Version 23.0. Armonk, NY: IBM Corp.

For peer review only - http://bmjopen.bmj.com/site/about/guidelines.xhtml

27. Dedoose Version 9.0.17, web application for managing, analyzing, and presenting qualitative and mixed method research data (2021). Los Angeles, CA: SocioCultural Research Consultants, LLC www.dedoose.com.
28. Bloomfield GS, Vedanthan R, Vasudevan L et al. Mobile health for non-communicable diseases in Sub-Saharan Africa: a systematic review of the literature and strategic framework for research. Global Health. 2014 Jun 13;10:49. doi: 10.1186/1744-8603-10-49.

For peer review only

For peer review only - http://bmjopen.bmj.com/site/about/guidelines.xhtml

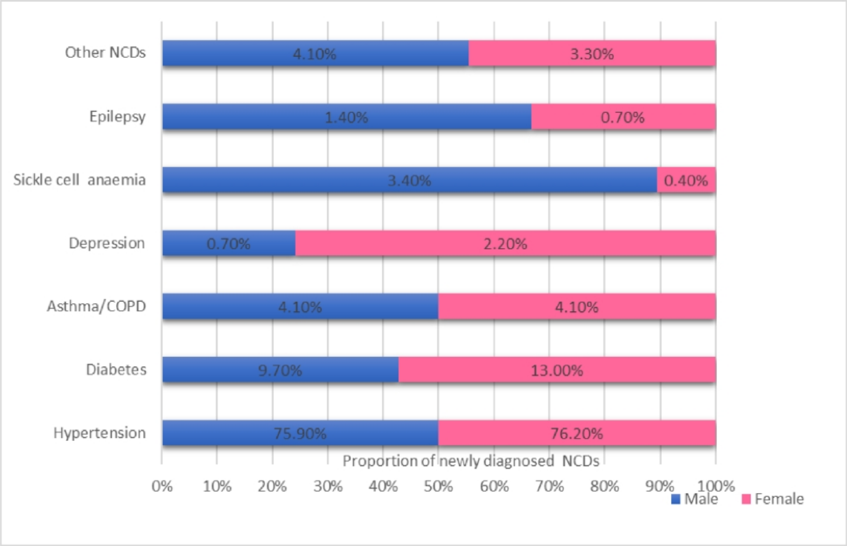

Figure 1. Gender distribution of newly diagnosed NCD cases

338x190mm (200 x 200 DPI)

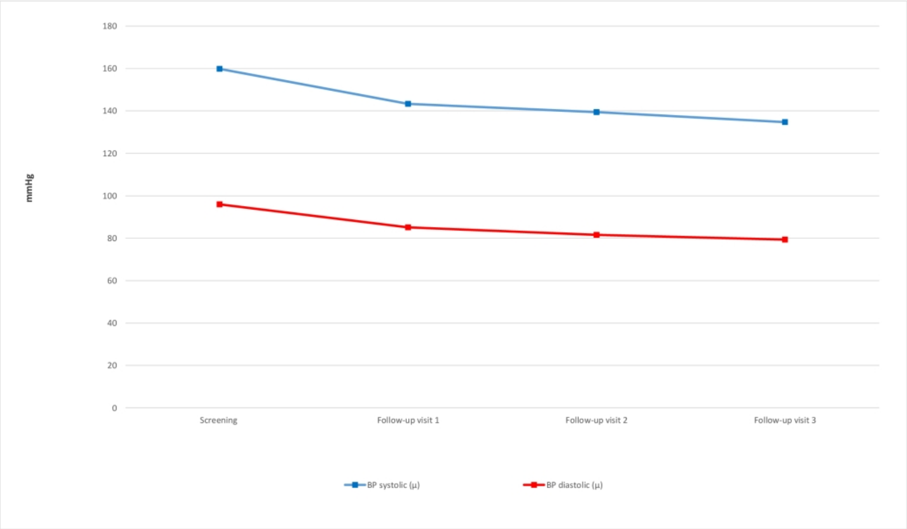

Figure 2. Trends in blood pressure change among hypertensive patients (n=428)

338x190mm (200 x 200 DPI)

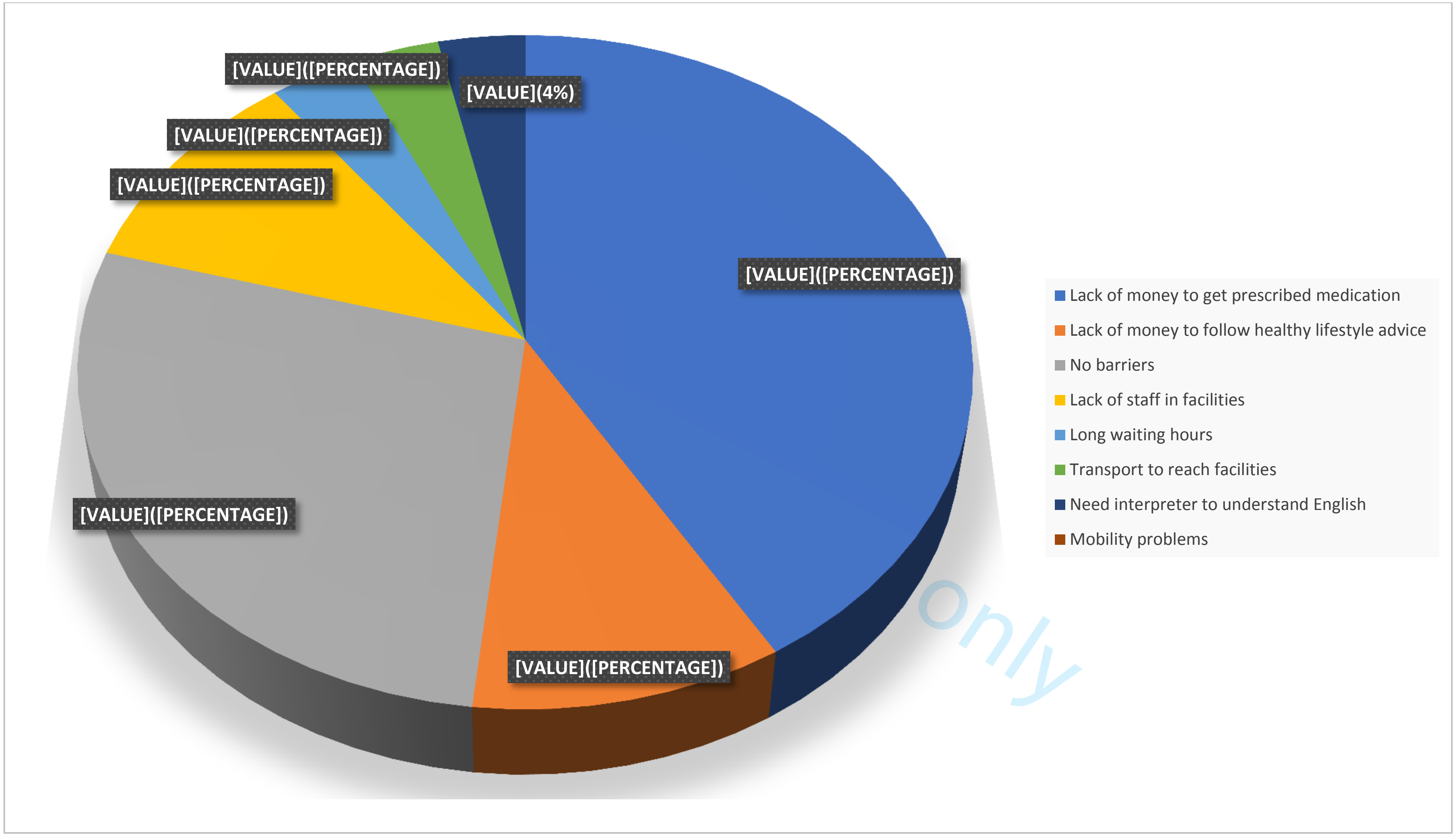

**Count shows times each barrier was mentioned*

Tackling non-communicable diseases in primary care facilities Nigeria through clinical guidelines and m-Health training strategies.

FOCUS GROUP DISCUSSION GUIDE

1. Perception of the use of the m-Health strategy- mode of delivery & content of training resources, challenges they have encountered as well as recommendations on the way forward.
- Satisfaction (or not) with the skills and knowledge from the training modules
- Were the modules sufficient enough to prepare you to carry out these services (NCD management)

2. Implementation issues;
    - Integrating NCD management into routine activities
    - Role of additional resources in program strengthening- clinical guidelines/ desk guides,
    - treatment cards, basic diagnostic equipment, monthly support for telecommunications

? – how did the project impact/ change your
-workload
-Income
-working relationship with colleagues
- relationship with community

3. Adequacy of Supervision:
    - Clinical supervisory visits (onsite and remote)
    - Monitoring
4. Moving forward, do you think your facility can continue providing these services even after the project ends?
5. Lessons learned

For peer review only

Standards for Reporting Qualitative Research (SRQR)*

http://www.equator-network.org/reporting-guidelines/srqr/

Title and abstract

	Page/line no(s).
**Title** - Concise description of the nature and topic of the study Identifying the study as qualitative or indicating the approach (e.g., ethnography, grounded theory) or data collection methods (e.g., interview, focus group) is recommended	Page 1, line 2-3
**Abstract** - Summary of key elements of the study using the abstract format of the intended publication; typically includes background, purpose, methods, results, and conclusions	Page 2, line 33-66

Introduction

**Problem formulation** - Description and significance of the problem/phenomenon studied; review of relevant theory and empirical work; problem statement	Page 4, line 113-133
**Purpose or research question** - Purpose of the study and specific objectives or questions	Page 4, line 213-130

Methods

**Qualitative approach and research paradigm** - Qualitative approach (e.g., ethnography, grounded theory, case study, phenomenology, narrative research) and guiding theory if appropriate; identifying the research paradigm (e.g., postpositivist, constructivist/ interpretivist) is also recommended; rationale**	Page 6 and 7, Line197-221
**Researcher characteristics and reflexivity** - Researchers' characteristics that may influence the research, including personal attributes, qualifications/experience, relationship with participants, assumptions, and/or presuppositions; potential or actual interaction between researchers' characteristics and the research questions, approach, methods, results, and/or transferability	Page 6, line 205 -211
**Context** - Setting/site and salient contextual factors; rationale**	Page 4, line 133-145
**Sampling strategy** - How and why research participants, documents, or events were selected; criteria for deciding when no further sampling was necessary (e.g., sampling saturation); rationale**	Page 7, line 245 - 252
**Ethical issues pertaining to human subjects** - Documentation of approval by an appropriate ethics review board and participant consent, or explanation for lack thereof; other confidentiality and data security issues	Page 20, line 656-659
**Data collection methods** - Types of data collected; details of data collection procedures including (as appropriate) start and stop dates of data collection and analysis, iterative process, triangulation of sources/methods, and modification of procedures in response to evolving study findings; rationale**	Page 7, line 245-252

For peer review only

For peer review only - http://bmjopen.bmj.com/site/about/guidelines.xhtml

**Data collection instruments and technologies** - Description of instruments (e.g., interview guides, questionnaires) and devices (e.g., audio recorders) used for data collection; if/how the instrument(s) changed over the course of the study	Page 7, line 245-252
**Units of study** - Number and relevant characteristics of participants, documents, or events included in the study; level of participation (could be reported in results)	Page 12, line 322-325
**Data processing** - Methods for processing data prior to and during analysis, including transcription, data entry, data management and security, verification of data integrity, data coding, and anonymization/de-identification of excerpts	Page 12, line 322-325
**Data analysis** - Process by which inferences, themes, etc., were identified and developed, including the researchers involved in data analysis; usually references a specific paradigm or approach; rationale**	Page 12, line 322-325
**Techniques to enhance trustworthiness** - Techniques to enhance trustworthiness and credibility of data analysis (e.g., member checking, audit trail, triangulation); rationale**	Page 12, line 322-325

Results/findings

**Synthesis and interpretation** - Main findings (e.g., interpretations, inferences, and themes); might include development of a theory or model, or integration with prior research or theory	Page 12-16
**Links to empirical data** - Evidence (e.g., quotes, field notes, text excerpts, photographs) to substantiate analytic findings	Page 17, line 540-579

Discussion

**Integration with prior work, implications, transferability, and contribution(s) to the field** - Short summary of main findings; explanation of how findings and conclusions connect to, support, elaborate on, or challenge conclusions of earlier scholarship; discussion of scope of application/generalizability; identification of unique contribution(s) to scholarship in a discipline or field	Page 3, line 62-72
**Limitations** - Trustworthiness and limitations of findings	Page 19, line 551-559

Other

**Conflicts of interest** - Potential sources of influence or perceived influence on study conduct and conclusions; how these were managed	Page 20, line 654
**Funding** - Sources of funding and other support; role of funders in data collection, interpretation, and reporting	Page 20, line 647-653

*The authors created the SRQR by searching the literature to identify guidelines, reporting standards, and critical appraisal criteria for qualitative research; reviewing the reference lists of retrieved sources; and contacting experts to gain feedback. The SRQR aims to improve the transparency of all aspects of qualitative research by providing clear standards for reporting qualitative research.

For peer review only

For peer review only - http://bmjopen.bmj.com/site/about/guidelines.xhtml

**The rationale should briefly discuss the justification for choosing that theory, approach, method, or technique rather than other options available, the assumptions and limitations implicit in those choices, and how those choices influence study conclusions and transferability. As appropriate, the rationale for several items might be discussed together.

Reference:

O'Brien BC, Harris IB, Beckman TJ, Reed DA, Cook DA. **Standards for reporting qualitative research: a synthesis of recommendations.** *Academic Medicine*, Vol. 89, No. 9 / Sept 2014 DOI: 10.1097/ACM.0000000000000388

For peer review only
